# Outcomes after corrective surgery for congenital dextro-transposition of the arteries using the arterial switch technique: a scoping systematic review

**DOI:** 10.1186/s13643-020-01487-3

**Published:** 2020-10-07

**Authors:** Frederick Morfaw, Alvin Leenus, Lawrence Mbuagbaw, Laura N. Anderson, Rejane Dillenburg, Lehana Thabane

**Affiliations:** 1 Department of Health Research Methods, Evidence and Impact, McMaster University, Hamilton, ON Canada; 2 Department of Obstetrics and Gynecology, Faculty of Medicines and Biomedical Sciences, University of Yaoundé 1, Yaoundé, Cameroon; 3 Faculty of Health Sciences, University of Bamenda, Bamenda, Cameroon; 4 Biostatistics Unit/FSORC, St Joseph Healthcare—Hamilton, 50 Charlton Avenue East, 3rd Floor Martha Wing, Room H321, Hamilton, ON L8N 4A6 Canada; 5 Centre for Development of Best Practices in Health, Yaoundé Central Hospital, Yaoundé, Cameroon; 6 Departments of Pediatrics and Anesthesia, McMaster University, Hamilton, Ontario Canada

**Keywords:** Transposition of great arteries, Jatene procedure, Arterial switch operation, Survival, Outcomes, Systematic review registration: PROSPERO CRD42014007590

## Abstract

**Background:**

Dextro-transposition of the great arteries (d-TGA) is the most frequent cyanotic congenital heart pathology in neonates. Surgical correction of this condition is possible using the arterial switch operation (ASO) which was first performed by Jatene in 1975.

**Objectives:**

The aim of this study was to summarise the evidence on short- (less than 1 year), medium- (1–20 years), and long-term (more than 20 years) outcomes of children with d-TGA treated with the ASO. The primary outcome was survival. Secondary outcomes were freedom from cardiac reoperations, occurrence of aortic insufficiency, pulmonary stenosis, coronary artery anomalies, neuropsychological development problems and quality of life.

**Methods:**

We searched MEDLINE, EMBASE, CINAHL, LILACS, and reference lists of included articles for studies reporting outcomes after ASO for d-TGA. Screening, data extraction and risk of bias assessment were done independently by two reviewers. We pooled data using a random-effects meta-analysis of proportions and, where not possible, outcomes were synthesized narratively. We used the Grading of Recommendations Assessment, Development and Evaluation (GRADE) system to assess the certainty of the evidence for each outcome.

**Main results:**

Following ASO for TGA, short-term survival was 92.0% (95% CI 91.0–93.0%; *I*^2^ = 85.8%, 151 studies, 30,186 participants; moderate certainty evidence). Medium-term survival was 90.0% (95% CI 89.0–91.0%; *I*^2^ = 84.3%, 133 studies; 23,686 participants, moderate certainty evidence), while long-term survival was 87.0% (95% CI 80.0–92.0 %; *I*^2^ = 84.5%, 4 studies, 933 participants, very low certainty evidence). Evaluation of the different secondary outcomes also showed satisfactory results in the short, medium and long term. Subgroup analysis suggests slightly higher survival following ASO for TGA in the second surgical era (1998 to 2018) than in the first surgical era (1975 to 1997) in the short and medium term [93.0% (95% CI 92.0–94.0) vs 90.0% (95% CI 89.0–92.0) and 93.0% (95% CI 91.0–94.0) vs 88.0% (87.0–90.0%) respectively] but not in the long term [81.0% (95% CI 76.0–86.0%) vs 89.0% (80.0–95.0%)].

**Conclusions:**

Pooled data from many sources suggests that the ASO for d-TGA leads to high rates of survival in the short, medium, and long term.

## Background

### Description of the condition

Dextro-transposition of the great arteries (d-TGA) also known as congenital complete transposition of the great arteries, or simply transposition of the great arteries (TGA) refers to a clinical condition in which the two main arteries leaving the heart (the pulmonary artery and the aorta) are transposed, with the aorta arising from the right ventricle, and the pulmonary artery originating from the left ventricle (ventriculoarterial discordance) [1]. It accounts for 3.0–10.0% of all congenital heart diseases in children [2]. The reported prevalence varies from 5.0 in 10,000 deliveries in the United States [3] to 1.6 per 10,000 children presenting to the hospital in Nigeria [4]. It is distinct from the levo-transposition of the great arteries (l-TGA) or congenitally corrected TGA which combines both an atrioventricular discordance and a ventriculoarterial discordance, with circulation continuing in the appropriate direction but via the “wrong” ventricles [5].

Unlike l-TGA whose patients may present for the first time in adulthood [6], d-TGA is the most frequent cyanotic congenital heart pathology in neonates and may be rapidly fatal at birth [7]. This is because, in the presence of d-TGA, the systemic and pulmonary circulations function in parallel rather than in series [8]. Consequently, deoxygenated blood from the right ventricle is pumped into the general systemic circulation through the aorta and returns back to the right ventricle; meanwhile, oxygenated blood from the left ventricle is pumped towards the lungs through the pulmonary artery and back to the left ventricle of the heart. Without some connection between the two systems, blood flow is ineffective and the condition will be fatal shortly after birth [8].

Even though physiological connections usually exist between the systemic and pulmonary systems in the fetus (atrial septum, ventricular septum, ductus arteriosus), these usually close up as the fetus approaches maturity. The persistence of these physiological connections ensuring the mixing of blood in the two systems is essential for survival, otherwise, these children become severely cyanosed and are at risk of death [1].

### The intervention

Surgical correction of this condition thereby ensuring the survival of these infants is possible, and the technique has evolved over time. The predominant surgical technique used today is the arterial switch operation (ASO) which was first performed by Jatene in 1975 [9]. The ASO involves repositioning of the great arteries of the heart as well as the coronary arteries, thereby separating the systemic from the pulmonary circulation, and restoring the role of the systemic ventricle to the left ventricle [9].

Conceptually, the ASO better mimics the natural anatomical and physiological circulations of the human circulatory system than the prior atrial switch operations pioneered by Mustard and Senning [10]. However, it is not without complications, which may include left ventricular failure, myocardial ischemia, pulmonary hypertension or supravalvar pulmonary stenosis [9].

### Why is it important to do this review?

With the refinement of the ASO technique over time, there has been a significant improvement in the outcomes of neonates affected by d-TGA. There is however very limited data summarising the long-term outcomes of neonates treated with ASO, and most of these were conducted in single centres. For example, Pretre et al. reported a 10-year survival probability of 93.7% (95% confidence interval [CI] 91.4–96.0%) for neonates undergoing ASO for d-TGA in France [11]. Horer et al. reported 10- and 20-year survival rates of 92.2% ± 1.3% and 90.4% ± 1.6% respectively amongst neonates undergoing ASO for d-TGA in Germany [12]. Khairy et al. reported 25-year survival rates of 96.7 ± 1.8% amongst neonates undergoing ASO in Boston USA [13]. More recently, Raissadati et al. reported 25-year survival rates of 97.0 % (95% CI 95.0–100.0 %) among patients undergoing ASO in Finland [14]. While these isolated reports provide a picture of outcomes related to the use of ASO, they are limited by their sample sizes and time frames. Even though ASO has been described as a “great success story”, the life-long consequences of this revolutionary surgical technique is purely speculative and needs time [15]. With close to 45 years of use, there is a wealth of information that has accumulated over a reasonable time frame to better inform us of just how successful ASO has been.

In this review, we will provide information on survival and other outcomes after treatment with ASO as outlined in the protocol [16].

## Objectives

The aim of this scoping review was to summarise the evidence on outcomes of children with d-TGA treated with ASO in order to inform clinicians, surgeons, researchers and policymakers on the impact of this technique. Specifically, we wish to determine the short-term (less than 1 year), medium-term (1–20 years) and long-term (more than 20 years) outcomes of children managed by ASO.

## Methods

This review was registered with the International prospective register of systematic reviews (PROSPERO), registration number CRD42014007590 [16]. The methods of this study are published elsewhere [16] but outlined here in brief. The review is reported according to the Preferred Reporting Items for Systematic reviews and Meta-Analyses extension for Scoping Reviews (PRISMA-ScR) (see Appendix 6) [17].

### Criteria for considering studies for this review

#### Types of studies

We included experimental and observational studies which evaluated survival, reoperations, mortality, aortic insufficiency, pulmonary stenosis, residual coronary abnormalities neuropsychological development and quality of life of neonates with d-TGA treated with ASO. For randomised trials, we used only data from the ASO arm. In cases where different technical aspects of the ASO were being compared, we used both arms. We included all studies eligible by the date of the literature search. We excluded commentaries and case reports on patients with d-TGA treated by ASO.

#### Types of participants

Our study population included all children born with classic d-TGA diagnosed before or at birth, who underwent ASO. Any studies with only a subset of the relevant participants were included and data extracted only for this subset of participants. We excluded all children undergoing ASO to correct other congenital cardiac anomalies. We equally excluded studies on children with “congenitally corrected” TGA or l-TGA, double outlet right ventricle with sub pulmonary stenosis, visceral heterotaxy and ambiguous atrial situs, right or left atrial isomerism, dextrocardia and situs inversus totalis and superoinferior ventricles.

#### Type of interventions

The intervention was ASO with or without the Lecompte manoeuvre (avoidance of the use of a prosthetic conduit in the reconstruction of the pulmonary outflow tract [18]), with or without ventricular septal defect closure, atrial septal defect or patent foramen ovale closure and ductus arteriosus ligation. We excluded studies in which ASO was used as corrective therapy for prior operations, atrial switch operations (Mustard and Senning) [19], Rastelli operation [20], and complex d-TGA repairs (reparation a l'etage ventriculaire or Nikaidoh procedures) [21].

#### Comparisons

No comparative data were used.

#### Outcomes

Our primary outcome was a survival from the moment of surgery. Secondary outcomes included freedom from cardiac reoperations, occurrence of aortic insufficiency, pulmonary stenosis, coronary artery anomalies, neuropsychological development problems and quality of life measures.

### Electronic searches

Using the OVID search platform, we searched MEDLINE from 1946 to October 2018, and EMBASE from inception (1974) to October 2018. We also searched the Cumulative Index to Nursing and Allied Health Literature (CINAHL) from inception (1981) till October 2018 using the EBSCOhost platform. Finally, we searched the Literature in the Health Sciences in Latin America and the Caribbean (LILACS) database using the Virtual Health Library regional portal from inception (1982) till October 2018. We set no limitations on language or the publication status of the studies. The last search in each of the databases was conducted on the 2nd of October 2018. (See appendix 1 for the MEDLINE search strategy).

### Searching other resources

We hand-searched the reference list of relevant studies and previous reviews as supplemental sources for studies that may have been missed in searches. We searched for grey literature such as unpublished conference proceedings from conference websites such as the Annual Meetings of the Spanish Society of Cardiology and the National Turkish Cardiology Conference in order to identify ongoing studies or completed but unpublished studies. Finally, we contacted experts in the field by email for any ongoing studies or relevant but unpublished studies.

### Data collection and analysis

#### Study selection

Two review authors (FM and AL) independently screened the titles and abstracts of all studies identified through the electronic searches in order to identify possible articles for inclusion. Following this screening, the full texts of eligible articles were obtained and assessed based on our inclusion criteria cited above.

#### Data extraction and management

We extracted data using a pre-designed and pre-tested data extraction form created using the web-based systematic review software DistillerSR [22]. The data extraction was done in duplicate by two authors (FM and AL) working independently. We extracted data on the study period, the country of conduct of the study, the language, the design, the sample size, the participant characteristics, and the different outcomes of interest as appropriate. In cases where data were reported solely as graphs, we contacted review authors by email for the raw data and, where not possible, we extrapolated directly from the graphs using the online tool WebPlotDigitizer [23]. In cases of incomplete data, missing data or uncertainty, we contacted the authors of the principal studies by email for clarifications. We extracted data on the various outcomes according to three-time frames, short term (less than 1 year), medium term (1–20 years) and long term (more than 20 years).

#### Assessment of risk of bias in included studies

We used the risk of bias tool for prevalence studies developed by Hoy et al. [24] to assess the risk of bias in studies included in the review. The tool has 10 items with the first 4 items assessing the external validity of the study (domains of selection and non-response bias). The remaining six items assess internal validity (measurement bias and bias related to analysis). According to the tool, individual items are rated as either at low or high risk of bias. In cases where there was not enough information to make a judgment for a given item, the study was rated as being at a high risk of bias for that item. The overall risk of bias was graded as either low, moderate or high [24]. Risks of bias assessments were done independently by two authors (FM and AL). We resolved any discrepancies in data handling by discussion or by consultation with a third author (LM).

#### Outcome measures

Statistical analyses were done using Stata statistical software version 13 [25]. The unit of analysis was the individual. For the primary outcome of survival, we extracted and assessed data on survival or mortality within the short, medium and long term. Secondary outcomes measures included: freedom from reoperation, aortic insufficiency, pulmonary stenosis, coronary artery anomalies, neuropsychological and quality of life outcomes of the infants. Similar to the primary outcome, we extracted and assessed data on these outcomes in the short, medium and long term following ASO as reported in the different studies.

For neuropsychological outcomes, the Bayley Scales for Infant Development version II (BSID-II) is one of the most widely used standardised tests to evaluate developmental retardation [26]. The Mental Development Index (MDI) from the BSID-II is a scale that measures mental abilities, such as memory and communication. The Physical Development Index (PDI) from the BSID-II measures motor abilities, such as body control and dynamic movement [26]. Neuropsychological outcomes we, therefore, extracted in these two domains.

For the quality of life measures, we considered reports which evaluated the quality of life of patients with d-TGA operated by the ASO, irrespective of the scale used to evaluate the quality of life. We extracted data on the general perception of health specifically.

#### Assessment of heterogeneity

We assessed statistical heterogeneity using visual inspection of the confidence interval of the pooled studies for any overlap, and also the chi^2^ test of homogeneity (statistical significance threshold *P* < 0.10) and the *I*^2^ statistic. We rated statistical heterogeneity based on the *I*^2^ statistic as described in the Cochrane Handbook of Systematic Reviews [27]. According to this guide, an *I*^2^ between 0.0 and 40.0% implies heterogeneity might not be important, an *I*^2^ of between 30.0 and 60.0% represents moderate heterogeneity, an *I*^2^ of between 50.0 and 90.0% represents substantial heterogeneity, while an *I*^2^ of between 75.0 and 100.0% represents considerable heterogeneity [27].

#### Data synthesis

We pooled the prevalence of reported outcomes across studies using the *metaprop* command in Stata software [28]. Given the risk of variance instability and undue overestimation of weights in meta-analysis in cases of extreme prevalences (values tending towards 0.0% or 100.0%), we pooled our results with prevalence estimates transformed using the Freeman-Tukey double arcsine transformation method [28]. The pooled estimate was then back-transformed to a summary estimate with its associated 95% confidence interval. In cases of outcomes measured on different scales, we standardised the scores to a single scale for meta-analysis. When this was not possible, we converted the values into a *Z* score which describes outcomes in standard deviation units. The result was a summary *Z* score which informed us of how many standard deviations the pooled estimate for patients with ASO for TGA differed from the population mean. We used a random-effects model to account for heterogeneity in all the analyses. For outcomes which could not be pooled together, a narrative synthesis of the outcome was conducted.

#### Subgroup analysis

We did a subgroup analysis according to the surgical era of operation. This was because we judged that with refinements in diagnostic criteria and the surgical technique, there might be variations in different surgical outcomes depending on the surgical area in which the procedure was conducted. Since the first ASO was performed in 1975 and we ran our study search till 2018, this gave a 43-years span. We, therefore, defined the first surgical area as running from 1975 to 1997 inclusive (first 22 years of ASO). We included in this category all studies in whom most of the patients were judged to have been operated within this timeframe. We defined the second surgical era as the period extending from 1998 until 2018 inclusive and included in this category all studies in whom most of the patients were judged to have been operated within this timeframe.

#### Sensitivity analysis

We conducted a sensitivity analysis by pooling the estimates using the untransformed data in order to determine the robustness of our conclusions. For outcomes synthesised by pooling estimates across the *Z* scores, a sensitivity analysis was not possible as Freeman-Tukey transformations could no longer be done.

#### Quality of the evidence

We evaluated the overall quality of the evidence for each of the key outcomes using the Grading of Recommendations Assessment, Development and Evaluation (GRADE) system for grading evidence described by Guyatt et al. [29]. We used the online GRADEpro Guideline Development Tool (GDT) [30] to construct a “Summary of findings” table (Appendix 4).

## Results

### Description of the search outcome

**Fig. 1:**
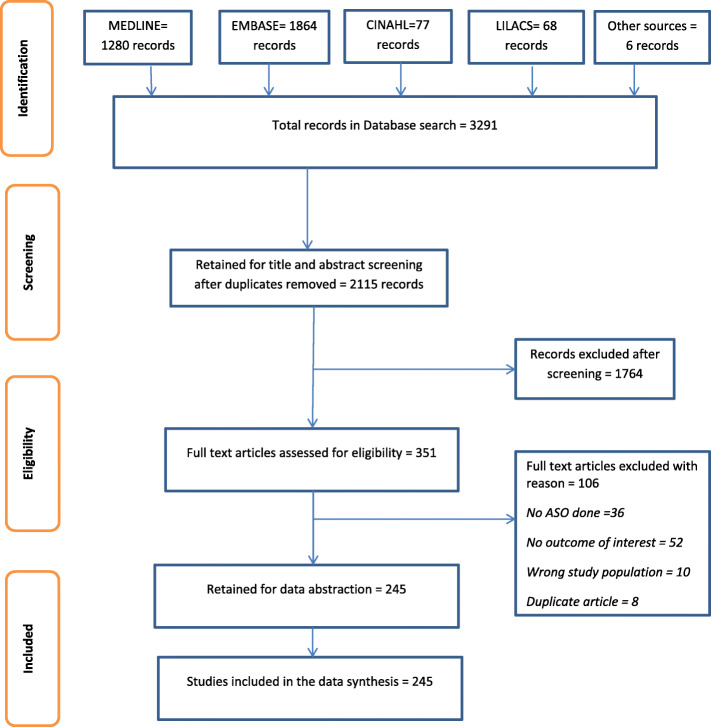
PRISMA flow diagram

The electronic databases and other resource search gave us a total of 2115 records after the removal of duplicates. The PRISMA diagram (Fig. 1) summarises the process of screening and selecting studies for inclusion in the review, and the number of studies retained at each stage. A total of 245 studies were retained for data extraction and included in the data synthesis.

### Included studies

Most of the included studies were retrospective cohort studies with sample sizes ranging from 9 to over 1200 patients, with follow-up times ranging from 1 to 32 years. Appendix 2 (characteristics of included studies) summarises the key features of the included studies.

### Excluded studies

A total of 104 studies were excluded after full-text review. Studies were mostly excluded either because they had the wrong intervention, the wrong study population, they involved the wrong procedure or did not report any outcomes of interest. Appendix 3 (characteristics of excluded studies) summarises the key features of the excluded studies.

### Risk of bias in included studies

We included an assessment of the risk of bias of each of the individual studies in the characteristics of included studies table (Appendix 2). About 30.0% of the studies were considered to be at high risk of bias for at least one of the outcomes.

### Study outcomes

**Fig. 2:**
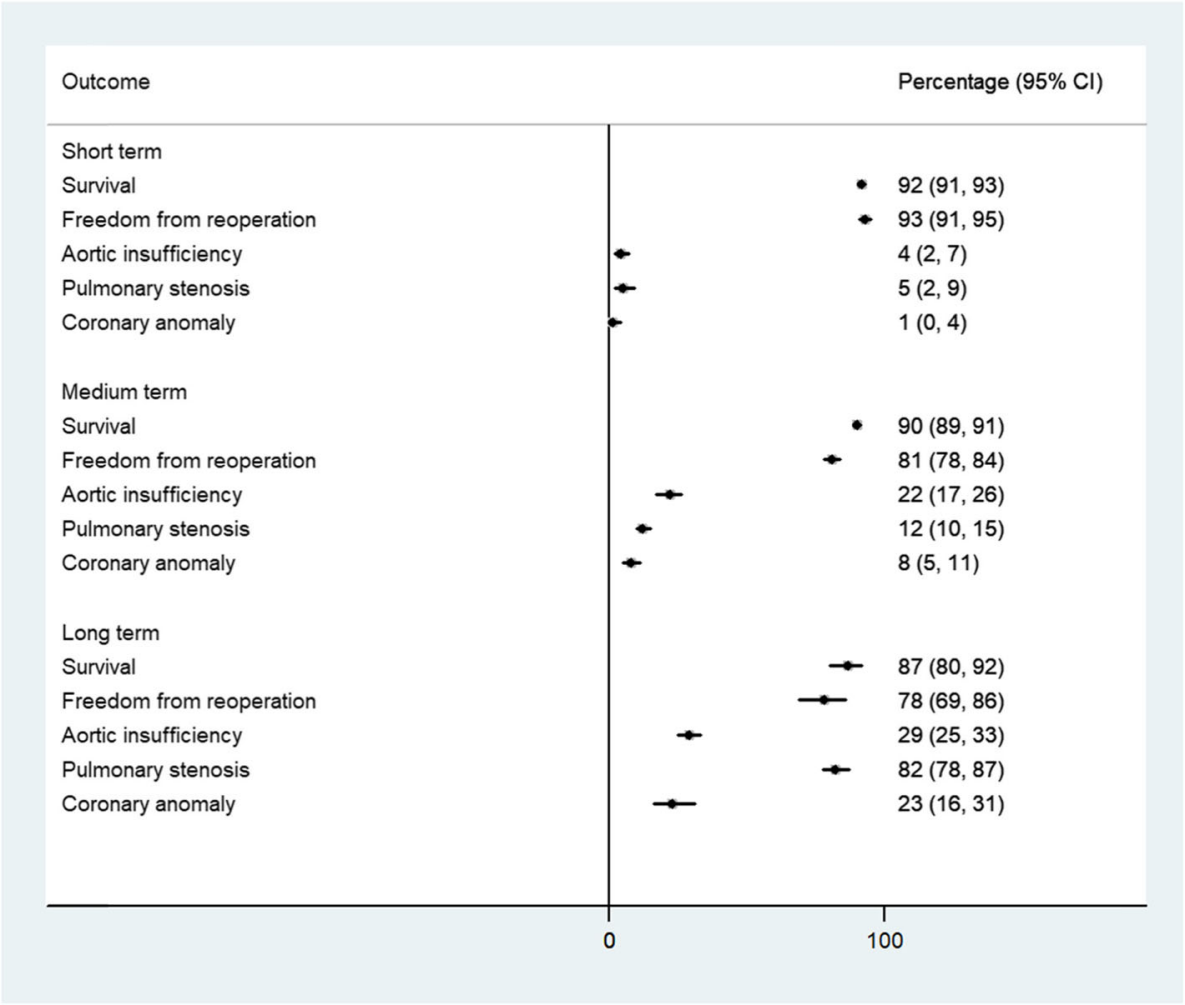
Summary of different study outcomes in the short term, medium and long term

**Fig. 3:**
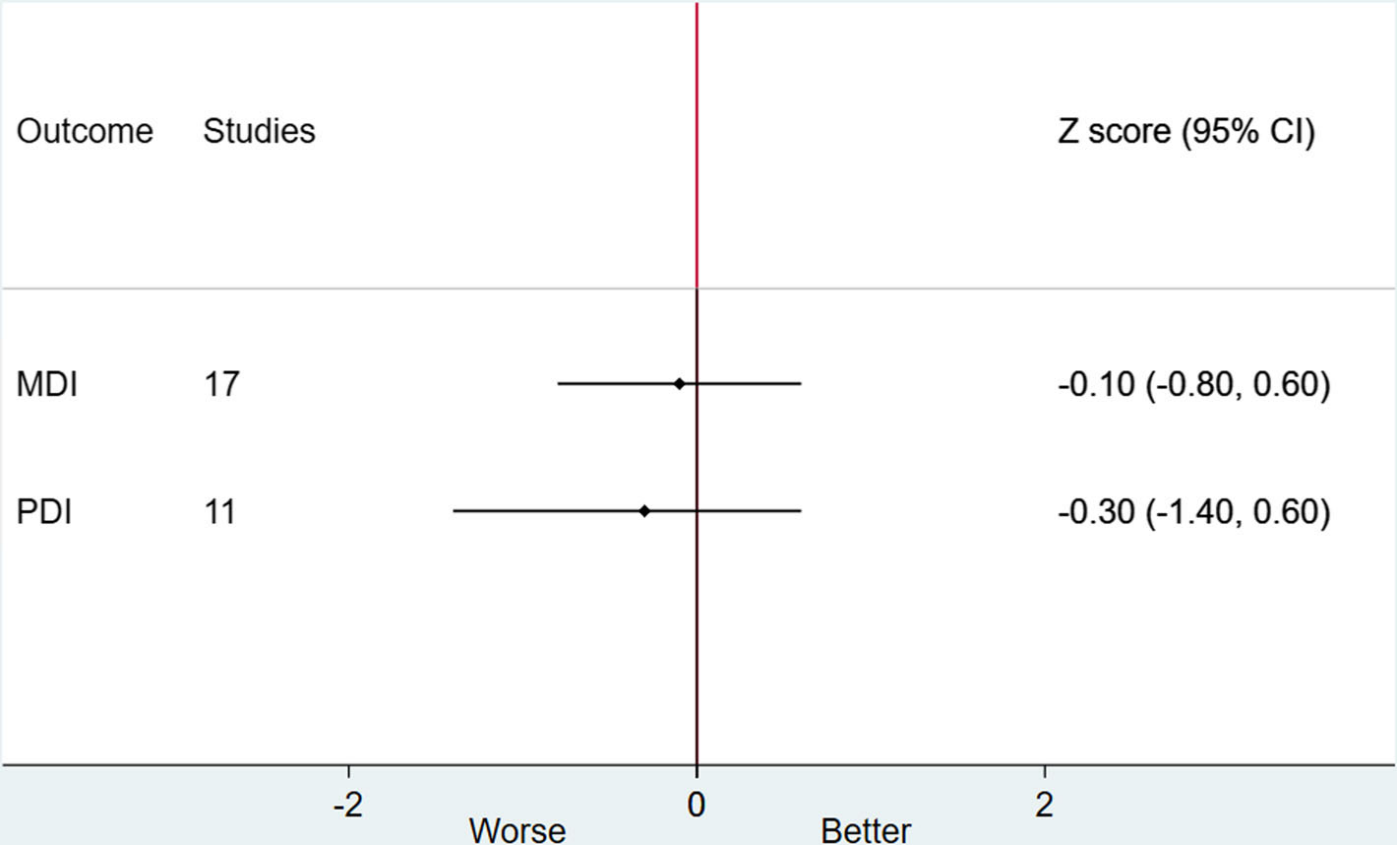
Summary of neuropsychological outcomes *z* scores for d-TGA patients at medium term. MDI = Mental Development Index. PDI = Physical Development Index

Table 1 summarises the main study outcomes overall and according to the surgical era (subgroup analysis). A “summary of findings” table for the different outcomes can be found in Appendix 4. Figure 2 summarises the different study outcomes in the short, medium and long term, while Fig. 3 specifically summarises neuropsychological outcomes reported only at medium term.

**Table 1:** Summary of main study outcomes

	Follow–up					
Outcome	Short-term (0–1 year)		Medium-term (1–20 years)		Long-term (>20 years)	
	% (95% CI)	Studies (*I*^2^ %)	% (95% CI)	Studies (*I*^2^ %)	% (95% CI)	Studies (*I*^2^ %)
**Survival**	92.0 (91.0**–**93.0)	151 (85.8)	90.0 (89.0–91.0)	133 (84.1)	87.0 (80.0–92.0)	4 (84.5)
Survival in the first era	90.0 (89.0**–**92.0)	62 (82.6)	88.0 (87.0–90.0)	70 (86.9)	89.0 (80.0–95.0)	3 (N/A)
Survival in the second era	93.0 (92.0**–**94.0)	89 (86.3)	93.0 (91.0–94.0	63 (78.1)	81.0 (76.0–86.0)	1 (N/A)
**Freedom from reoperation**	93.0 (91.0**–**95.0)	43 (92.3)	81.0 (78.0–84.0)	110 (95.6)	78.0 (69.0–86.0)	6 (95.5)
Freedom from reoperation in the first era	96.0 (94.0**–**97.0)	22 (89.4)	83.0 (79.0–86.0)	62 (95.6)	75.0 (63.0–85.0)	4 (94.9)
Freedom from reoperation in the second era	90.0 (85.0**–**94.0)	21 (92.3)	79.0 (74.0–84.0)	48 (95.9)	85.0 (82.0–87.0)	2 (N/A)
**Aortic insufficiency**	4.0 (2.0**–**7.0)	19 (96.2)	22.0 (17.0–26.0)	65 (96.9)	29.0 (25.0–33.0)	2 (N/A)
Aortic insufficiency in the first era	1.0 (0.0**–**5.0)	11 (95.8)	18.0 (12.0–25.0)	30 (97.6)	41.0 (35.0–48.0)	1 (N/A)
Aortic insufficiency in the second era	9.0 (2.0**–**15.0)	8 (94.2)	25.0 (18.0–32.0)	35 (95.9)	19.0 (14.0–24.0)	1 (N/A)
**Pulmonary stenosis**	5.0 (2.0**–**9.0)	9(85.5)	12.0 (10.0–15.0)	54 (92.8)	82.0 (77.0–86.0)	1 (N/A)
Pulmonary stenosis in the first era	8.0 (3.0**–**15.0)	2 (N/A)	12.0 (9.0–16.0)	28 (93.2)	—*	0 (N/A)
Pulmonary stenosis in the second era	4.0 (1.0**–**8.0)	7 (86.5)	12.0 (8.0–17.0)	26 (92.3)	82.0 (77.0–86.0)	1 (N/A)
**Coronary anomaly**	1.0 (0.0**–**4.0)	5 (59.7)	8.0 (5.0–11.0)	37 (93.3)	23.0 (16.0–31.0)	2 (N/A)
Coronary anomaly in the first era	1.0 (0.0**–**3.0)	1 (N/A)	4.0 (2.0–6.0)	16 (87.6)	--*	0 (N/A)
Coronary anomaly in the second era	1.0 (0.0**–**5.0)	4 (53.9)	12.0 (7.0–18.0)	21 (94.0)	23.0 (16.0–31.0)	2 (N/A)
**Neuropsychological outcome MDI** ***z*** score **	*	—*	−0.1 (−0.8–0.6)	17 (0.0)	—*	—*
Neuropsychological outcome MDI *z* score** in the first era	—*	—*	−0.0 (−0.9–0.8)	10 (0.0)	—*	—*
Neuropsychological outcome MDI *z* score** in the second era	—*	—*	−0.3 (−1.9–1.2)	7 (0.0)	—*	—*
**Neuropsychological outcome PDI** ***z*** score**	—*	—*	−0.3 (−1.3–0.7)	11 (0.0)	—*	—*
Neuropsychological outcome PDI *z* score** in the first era	—*	—*	−0.4 (−1.6–0.9)	5 (0.0)	—*	—*
Neuropsychological outcome PDI *z* score** in the second era	—*	—*	−0.3 (−1.9–1.3)	6 (0.0)	—*	—*
**Quality of life**	—*	—*	Assessed with 7 studies. Three studies found no difference in Health-Related Quality of Life (HRQoL) of ASO patients relative to the general population; two studies found that ASO patients had better HRQoL, while two others found they had lower HRQoL.		—*	—*

*N/A* not applicable (in these cases, the outcome was not computed by the statistical software), *MDI* Mental Development Index, *PDI* Physical Development Index, *HRQoL* Health-related quality of life, *ASO* Arterial switch operation.

*No studies reported on this outcome during the specified time frame.

**The *Z* score compares the number of standard deviations the score of the d-TGA patients differs from that of the general population, with zero being the score of no difference, lower scores being poor compared with the general population, and higher scores being better

#### Survival

- Short-term survival (0–1 year): The overall short-term survival rate was 92.0% (95% CIs 91.0–93.0%) with a high level of heterogeneity (*I*^2^ = 85.8%, 151 studies, 30,186 participants, moderate quality evidence). The pooled short-term survival rate in the first era was 90.0% (95% CI 89.0–92.0%) (*I*^2^ 82.6%, 62 studies, 12,875 participants). The pooled short-term survival in the second era was 93.0% (95% CI 92.0–94.0%) (*I*^2^ 86.3%, 89 studies, 17,311 participants).
- Medium-term survival (1–20 years): The overall medium-term survival rate was 90.0% (95% CIs 89.0–91.0%) with a high level of heterogeneity (*I*^2^ = 84.1%, 133 studies, 23,686 participants, moderate quality evidence). The pooled medium-term survival rate in the first era was 88.0% (87.0–90.0%) (*I*^2^ 86.9, 70 studies, 15,403 participants). The pooled medium-term survival in the second era was 93.0% (91.0–94.0%) (*I*^2^ 78.1%, 63 studies, 8283 participants).
- Long-term survival (>20 years): The pooled long-term survival rate was 87.0% (95% CIs 80.0–92.0%) with a high level of heterogeneity (*I*^2^ = 84.5%, 4 studies, 933 participants, very low-quality evidence). The pooled long-term survival rate in the first era was 89.0% (80.0–95.0) (*I*^2^ unavailable, 3 studies, 692 participants). The pooled long-term survival rate in the second era was 81.0% (76.0–86.0%) (*I*^2^ not estimable, 1 study, 241 participants).

#### Freedom from reoperation

- Short-term freedom from reoperation (0–1 years): The pooled prevalence for short-term freedom from reoperation after ASO for TGA was 93.0% (95% CIs 91.0–95.0%) with a high level of heterogeneity (*I*^2^ = 92.3%, 43 studies, 8391 participants, moderate-quality evidence).
- Medium-term freedom from reoperation (1–20 years): The pooled prevalence for medium-term freedom from reoperation after ASO for TGA was 81.0% (95% CIs 78.0–84.0%) with a high level of heterogeneity (*I*^2^ = 95.8%, 110 studies, 21,269 participants, moderate-quality evidence).
- Long-term freedom from reoperation (>20 years): The pooled prevalence for long-term freedom from reoperation after ASO for TGA was 78.0% (95% CIs 69.0–86.0%) with a high level of heterogeneity (*I*^2^ = 95.5%, 6 studies, 2359 participants, very low-quality evidence).

#### Aortic insufficiency

- Short-term occurrence of aortic insufficiency (0–1 year): The pooled prevalence for short-term occurrence of aortic insufficiency after ASO for TGA was 4.0% (95% CIs 2.0–7.0%) with a high level of heterogeneity (*I*^2^ = 96.2%, 19 studies, 5354 participants, low-quality evidence).
- Medium-term occurrence of aortic insufficiency (1–20 years): The pooled prevalence for medium-term aortic insufficiency after ASO for TGA was 22.0% (95% CIs 17.0–26.0%) with a high level of heterogeneity (*I*^2^ = 96.9%, 65 studies, 10,705 participants, moderate-quality evidence).
- Long-term occurrence of aortic insufficiency (>20 years): The pooled prevalence for long-term aortic insufficiency after ASO for TGA was 29.0% (95% CIs 25.0–33.0%) (*I*^2^ unavailable, 2 studies, 461 participants, very low-quality evidence).

#### Pulmonary stenosis

- Short-term occurrence of pulmonary stenosis (0–1 years): The pooled prevalence for short-term occurrence of pulmonary stenosis after ASO for TGA was 5.0% (95% CIs 2.0–9.0%) with a high level of heterogeneity (*I*^2^ = 85.5%, 9 studies, 1150 participants, moderate-quality evidence).
- Medium-term occurrence of pulmonary stenosis (1–20 years): The pooled prevalence for medium-term aortic insufficiency after ASO for TGA was 12.0% (95% CIs 10.0–15.0%) with a high level of heterogeneity (*I*^2^ = 92.8%, 54 studies, 8607 participants, moderate-quality evidence).
- Long-term occurrence of pulmonary stenosis (>20 years): Only one study [31] reported on this outcome. The prevalence of long-term pulmonary stenosis was 82.3% (95% CIs 77.5–87.1%) (1 study, 241 participants, very low certainty evidence). A sensitivity analysis was not conducted for this outcome.

#### Coronary anomaly

- Short term occurrence of any coronary anomaly (0-1 years): The pooled prevalence for short term occurrence of any coronary anomaly after ASO for TGA was 1.0 % (95% CIs: 0.0 – 4.0 %) with a moderate level of heterogeneity (I^2^ = 59.7%, 5 studies, 611 participants, low certainty evidence).
- Medium-term occurrence of any coronary anomaly (1–20 years): The pooled prevalence for medium-term occurrence of any coronary anomaly after ASO for TGA was 8.0% (95% CIs 5.0–11.0%) with a high level of heterogeneity (*I*^2^ = 93.3%, 37 studies, 6542 participants, low-certainty evidence).
- Long-term occurrence of any coronary anomaly (>20 years): The pooled prevalence for long-term occurrence of any coronary anomaly after ASO for TGA was 23.0% (95% CIs 16.0–31.0%) (*I*^2^ unavailable, 2 studies, 134 participants).

#### Neuropsychological outcomes (mental development and physical development) at medium-term (1–20 years)

- Mental development index: The pooled weighted *Z* score for the mental development index after ASO for TGA was −0.1 (95% CIs −0.8–0.6) with a little to no heterogeneity (*I*^2^ = 0.0 %, 17 studies, 1419 participants, moderate-certainty evidence).
- Physical development index: The pooled weighted *Z* score for the physical development index after ASO for TGA was −0.3 (95% CIs −1.3–0.6) with a little to no heterogeneity (*I*^2^ = 0.0 %, 17 studies, 1419 participants, moderate certainty evidence).

### Quality of life outcomes

Seven studies evaluated the quality of life of ASO survivors [32–38]. They used different scales and reported on different aspects, hence a meta-analysis of the results was not possible, and the results were narratively summarised (see Table 2). The general tendency was that there was no substantial difference in the quality of life between ASO patients and the comparison groups.

**Table 2:** Summary of quality of life outcomes

Study: Main feature and scale used for quality of life assessment	Outcome
Fricke et al [32]: Compared the quality of life of 107 ASO patients with that of an age-matched Australian population using Short Form 6-Dimension (SF6D).	No statistically significant difference in the mean SF6D scores between ASO patients and the general Australian population (18–24 age group: [0.769 for ASO patients vs 0.772 for Australian population, *P* = 0.85]; 25–34 age group [0.795 for ASO patients vs 0.780 for Australian population, *P* = 0.33]).
Kalfa et al [33]: Evaluated the cognitive and psychological outcomes of 67 ASO adults (18.0–31.0 years) compared to 43 matched controls using the Short Form 36 (SF-36).	ASO patients had a statistically significant lower physical component summary compared to controls (52.1 ± 7.5 vs 55.4 ± 5.9, *P* = 0.01). No statistically significant difference in the mental component summary (47.2 ± 10.3 vs 44.5 ± 11.5).
Ruys et al [34]: Reported the quality of life of 18 ASO patients (22.0–25.0 years), and compared them with healthy controls and patients who underwent the Mustard procedure for TGA using the Short Form 36.	They reported that ASO patients scored significantly better than the normal Dutch population on domains of physical functioning, vitality and role limitations due to emotional problems (*P* < 0.01) [34].
Gorler et al [35]: Compared the quality of life of 98 ASO adults survivors against those of atrial repair patients and the general population and reported the Z scores from the Short Form 36.	They did not find any statistically significant difference in quality of life between ASO patients and the comparison groups. They however noted a tendency for better results in the categories of “general health” and “role emotional” for the ASO patients [35].
De Koning et al [36]: Compared the quality of life of 31 ASO child survivors age 8.0 -15.0 years against children of the same age from the general Dutch population using the TNO-AZL child quality of life (TACQOL) questionnaire.	They noted poorer health related quality of life (HRQOL) in motor functioning and positive emotional functioning among ASO children, with no further differences on other TACQOL scales [36].
Hovels-Gurich et al [37]: Compared the quality of life of 60 ASO child survivors (mean age 10.5 years) against children of the same age from the general German population using the Inventory for the Assessment of the Quality of Life in Children and Adolescents (IQLC).	They did not find any statistically significant difference in total quality of life between ASO children and healthy controls (mean score [standard deviation SD] 1.62 [0.66] vs 1.64 [0.59] *P* > 0.20) [37].
Dunbar-Masterson et al [38]: Compared the quality of life of 155 ASO child survivors (median age 8.1 years) against a normative sample of children of the same age from the general American population using the Child Health Questionnaire, Parent Form-50.	They found similar Physical Health Summary (mean score [SD] 54.0 [6.1] vs 53.0 [8.8]) and Psychosocial Summary scores (mean score [SD] 49.7 [9.9] vs 51.2 [9]) between ASO children and healthy controls [38].

*ASO* Arterial switch operation, *TGA* Transposition of the great arteries, *SF6D* Short Form 6-Dimension, *SF-36* Short Form 36, *TACQOL* TNO-AZL child quality of life questionnaire, *HRQOL* Health-related quality of life, *IQLC* Inventory for the Assessment of the Quality of Life in Children and Adolescents

### Sensitivity analysis

A sensitivity analysis using the untransformed prevalence estimates showed similar results for the different outcomes and time frames (see Appendix 5).

## Discussion

### Summary of main results

Following ASO for TGA, short-term survival was 92.0% (95% CI 91.0–93.0 %; *I*^2^ = 85.8%, 151 studies, 30,186 participants; moderate-certainty evidence). Medium-term survival was 90.0% (95% CI 89.0–91.0%; *I*^2^ = 84.3%, 133 studies; 23,686 participants, moderate-certainty evidence), while long-term survival was 87.0% (95% CI 80.0–92.0%; *I*^2^ = 84.5%, 4 studies, 933 participants, very low-certainty evidence). Evaluation of the different secondary outcomes also showed satisfactory results in the short, medium and long-term. Sensitivity analysis showed similar results. Subgroup analysis suggests slightly higher survival following ASO for TGA in the second surgical era (1998 to 2018) than in the first surgical era (1975 to 1997) in the short and medium term [93.0% (95% CI 92.0–94.0) vs 90.0% (95% CI 89.0–92.0) and 93.0% (95% CI 91.0–94.0%) vs 88.0% (87.0–90.0%) respectively] but not in the long term [81.0% (95% CI 76.0–86.0%) vs 89.0% (80.0–95.0%)].

### Agreement and disagreement with other reviews

We identified several review articles which had attempted to summarise different outcomes following arterial switch operation [39–46]. However, most of these synthesized the data narratively, and none presented a complete overview of the different outcomes over the different timeframes evaluated in this review.

Our estimates for survival in the short, medium and long term were similar to those from other studies which reported short-term survival rates of about 95.0% [39, 40], medium-term survival rates of about 88.0–97.0% [39–42], and long-term survival rates of about 90.0–96.7% [39, 42]. Many factors have been reported as being predictors of early survival following ASO for TGA. These include complex anatomy, coronary anomalies, an extended coronary artery bypass time, low APGAR-score, preterm birth, the need for additional procedures, and surgeries performed either too soon or too late [47, 48]. Our study did not account for these and other predictors of survival. However, our findings suggest improved survival in the second surgical era relative to the first surgical era. It has been hypothesized that improvements in surgical technique, intensive care unit treatment and the better clinical condition of patients presenting in the second era may account for these results [48].

Staying free from cardiac surgical reoperation after ASO for d-TGA may be as important as surviving over the years. Yet few reviews have attempted to gather the evidence on freedom from cardiac surgical reoperation following ASO for TGA. Based on fewer studies than in our report, Tabitha et al. reported a 10-year freedom from reoperation rate at 82.0% [42], a finding similar to the 81.0% (78.0–84.0%) medium-term freedom from reoperation in our study. There is limited evidence on long-term freedom from reoperation for these patients.

The most frequent complication of ASO for TGA is supravalvular pulmonary stenosis [43]. Our estimates for pulmonary stenosis in the short and medium term are similar to those from other studies which reported short-term pulmonary stenosis rates of 4.0–11.0% [40] and medium-term pulmonary stenosis rates between 2.0 and 30.0% [39, 43]. Considering the potential morbidity associated with pulmonary stenosis and the need for reintervention in many cases, an improvement in the ASO technique to help minimize this complication is important.

Reports of the prevalence of aortic insufficiency varied across different reviews. Raja et al. reported a midterm prevalence of aortic insufficiency of about 35.0% [40], a value much higher than the <7.0% prevalence rate of severe aortic insufficiency reported by Vargo et al. in their narrative review [44]. Our point estimate of a 22.0% prevalence of aortic insufficiency does not concur with either of these values. Our value however falls within the wider estimates for aortic insufficiency varying from 0.0–50.0% reported by Blume et al. [43]. Blume et al. noted that this variation is mainly related to the different diagnostic methods used [43].

The risk of coronary artery occlusion following ASO which can compromise myocardial perfusion is real [44]. Our estimates for coronary artery occlusion in the medium term are similar to those from other studies which reported coronary artery obstructive lesions to range between 2.0 and 11.0% in the medium term [40, 42, 44]. Our medium-term estimate of coronary artery anomaly of 8.0% is however higher than the 2.0–5.0% prevalence rate reported by Blume et al. earlier on [43]. This difference is probably due to the fact that Blume et al. used a narrative review to describe this outcome and fewer studies than those included in our meta-analysis.

Few reviews have reported on child development outcomes following d-TGA. A narrative review by Kasmi et al. reported scores on the Bayley Scales of Infant Development (BSID) to be 0.5–1.0 standard deviation (SD) below the expected mean values, suggestive of mild to moderate delays in cognitive, motor and language functions [46]. These contrast with our findings which do not show any statistically significant difference between mean values on the BSID reported by TGA patients post-ASO and their normal counterparts. The summary estimate provided by Kasmi et al. [46] is based on a narrative synthesis of four studies, all of which were included in our meta-analysis, in addition to 13 other studies. Our findings suggest that these patients could have a normal neuropsychological life, at least in the medium term.

In a systematic review assessing the quality of life of adult congenital heart disease patients, Fteropoulli et al. [49] highlighted that methodological limitations of defining and measuring quality of life in different studies hindered valid conclusions to be made in this domain in general. We agree with this conclusion as the studies included in our review used varied tools and tended to measure different aspects of quality of life, making it hard to provide a general conclusion on the quality of life of these patients.

### Strengths of this review

The strength of this review lies in its breadth, providing a concise summary evidence for different outcomes in children treated with ASO for d-TGA. Our findings also find strength in the number of included studies and participants. We employed a comprehensive literature search of pertinent databases without any language restriction. Screening, data extraction and risk of bias assessments were done independently by two reviewers, minimising biases in the data extraction and the evidence synthesis.

### Potential limitations in the review

Given that this review summarises evidence from mostly observational studies with varying methodologies and varying methods of outcome ascertainment and reporting, the overall risk of bias remains pertinent. Furthermore, our study does not adjust for potential risk factors which may lead to variations in the reported outcomes in the different studies. Moreover, advancements in surgical technique and surgical expertise over time suggest that outcomes for this procedure are bound to vary with time. Our review does not capture a time distinction which may mark an evolutionary advancement in the ASO expertise, beyond which patient outcomes may differ from initial cohorts. This is because even though we evaluated outcomes according to the surgical era, this distinction was at best approximative. We did not have access to individual patient data from all included studies to precisely categorize patients according to surgical era. Follow-up periods most likely overlapped across our boundaries for the surgical era, and as such the results of the subgroup surgical era analyses need to be interpreted with caution.

### Overall completeness and applicability of the evidence

We employed a comprehensive and broad search strategy and are confident that the right participants, intervention (ASO), and outcomes have been explored with this evidence. We included a total of 245 studies, making this the most complete body of evidence on outcomes after ASO for d-TGA to date. Most of the studies included in this review were conducted in high- and middle-income countries, and this may limit the applicability of the findings especially in underdeveloped settings where we hardly found evidence for the conduct of ASO. This may reflect publication bias which was detected (result not shown) in the assessment of certain outcomes, thus limiting the overall completeness of our evidence.

#### Quality of the evidence

Overall, our judgment of the quality of evidence for the outcomes in this review is moderate to very low (Appendix 4). This is primarily due to the study design of the majority of the included studies, as well as the inconsistencies of results and poor quality of reporting for certain outcomes.

#### Implications for clinical practice

Improvements in the diagnostic techniques for TGA are important if these children are to subsequently benefit from the successes of ASO. There is a need for dissemination of the diagnostic and surgical technique such that it may reach all those affected especially in low-income countries. There is also a need for the continued advancement of the surgical technique and comprehension of the potential complications.

#### Implications for policy

Babies undergoing ASO for d-TGA remain a unique group of individuals who need to be closely monitored throughout their lives. The inconsistency and poor quality of reporting on outcomes of these patients seen in different studies suggest a need for improved health policy on surveillance and documentation of outcomes for these patients.

#### Implications for research

Our research identified some evidence of publication bias in reporting certain outcomes of ASO patients. It is important for researchers to publish all results, be they good or bad, if we are to have a true picture of the impact of an intervention. Our study highlights a research gap on the limited amount of research done on ASO for TGA in the long term (beyond 20 years). Despite the fact that this procedure has been around for 40 years and above, very few studies have done long term follow-up on different outcomes of these patients beyond 20 years.

## Conclusions

The ASO for TGA leads to high survival rates and few morbidities. More long-term research is encouraged to monitor outcomes throughout the duration of life for children who receive the ASO.

## Supplementary information

**Additional file 1.** Appendix 1 MEdline search strategy 2 October 2018

**Additional file 2.** Appendix 2 Characteristics of included studies.

**Additional file 3.** Appendix 3 Characteristics of excluded studies with reasons

**Additional file 4.** Appendix 4 Summary of findings table: Arterial switch operation for transposition of the great arteries.

**Additional file 5.** Appendix 5 Sensitivity analysis for the main and secondary outcomes .

**Additional file 6.** Appendix 6 Preferred Reporting Items for Systematic reviews and Meta-Analyses extension for Scoping Reviews (PRISMA-ScR) Checklist.

## Data Availability

Data sharing is not applicable to this article as no datasets were generated or analysed during the current study. A list of additional material is found in the appendix, and references made to them within the manuscript.
