# Supplementary material for: Outcomes after corrective surgery for congenital dextro-transposition of the arteries using the arterial switch technique: a scoping systematic review
Source: Syst Rev. 2020 Oct 7;9:231. doi: 10.1186/s13643-020-01487-3 (PMC7542944; doi:10.1186/s13643-020-01487-3)
Supplement: Supplementary file 1 — Additional file 1. Appendix 1 MEdline search strategy 2 October 2018 [file 13643_2020_1487_MOESM1_ESM.docx]

**Appendix 1: MEdline search strategy 2 October 2018**

Database(s): OVID Medline Epub Ahead of Print, In-Process & Other Non-Indexed Citations, Ovid MEDLINE(R) Daily and Ovid MEDLINE(R) 1946 to Present 
Search Strategy:

| **#** | **Searches** | **Results** |
| --- | --- | --- |
| 1 | arterial switch operation.mp. | 1213 |
| 2 | arterial switch procedure.mp. | 175 |
| 3 | jatene.mp. | 117 |
| 4 | exp "Transposition of Great Vessels"/ | 7340 |
| 5 | transposition of the great arteries.mp. | 4795 |
| 6 | Complete transposition.mp. | 839 |
| 7 | simple transposition.mp. | 218 |
| 8 | dextro-transposition.mp. | 114 |
| 9 | d-transposition.mp. | 630 |
| 10 | ventriculoarterial discordance.mp. | 93 |
| 11 | discordant ventriculoarterial connection.mp. | 28 |
| 12 | intact ventricular septum.mp. | 1258 |
| 13 | exp Heart Septal Defects, Ventricular/ | 11749 |
| 14 | lecompte.mp. | 103 |
| 15 | outcomes.mp. | 768895 |
| 16 | survival.mp. | 1096085 |
| 17 | 1 or 2 or 3 | 1418 |
| 18 | 4 or 5 or 6 or 7 or 8 or 9 or 10 or 11 or 12 or 13 or 14 or 15 or 16 | 1770816 |
| 19 | 17 and 18 | 1280 |
