# Supplementary material for: Outcomes after corrective surgery for congenital dextro-transposition of the arteries using the arterial switch technique: a scoping systematic review
Source: Syst Rev. 2020 Oct 7;9:231. doi: 10.1186/s13643-020-01487-3 (PMC7542944; doi:10.1186/s13643-020-01487-3)
Supplement: Supplementary file 2 — Additional file 2. Appendix 2 Characteristics of included studies. [file 13643_2020_1487_MOESM2_ESM.docx]

**Appendix 2: Characteristics of included studies**

| **Study** | **Study Period** | **City, Country** | **Language** | **Design** | **Aim** | **Inclusion Criteria** | **n/m** | **Key message** |
| --- | --- | --- | --- | --- | --- | --- | --- | --- |
| Quaegebeur 1986 (1) | 1977-1985 |  | English | Cohort study | To describe experiences with ASO for TGA | ASO for TGA | 56/66 | Comparison with the results of the atrial switch repair indicates that the arterial switch repair is superior. |
| Sidi 1987 (2) | 1984-1986 | Paris, France | English | Cohort study | To describe outcomes of treatment of TGA by anatomic correction | ASO for TGA | 50/50 | Anatomic correction can be applied successfully in the first few days of life in newborns with simple transposition of the great arteries, regardless of coronary distribution. |
| Wernovsky 1988 (3) | 1983-1987 | USA | English | Cohort study | To evaluate the theoretical advantages of ASO | ASO for TGA | 100/100 | Arterial switch operation is the procedure of choice for neonates with TGA and intact intraventricular septum. |
| Donato 1989 (4) | 1983-1987 | USA | English | Cohort study | To describe experiences with ASO for TGA and double outlet ventricle | ASO for TGA | 56/62 | The low hospital mortality and encouraging early follow-up results support ASO as the procedure of choice for children who have TGA with ventricular septal defect or double outlet ventricle |
| Backer 1989 (5) | 1968-1988 | Chicago, USA | English | Cohort study | To compare the results of the Mustard procedure versus ASO for TGA | ASO for TGA | 37/60 | ASO has early and late mortality comparable with those of the Mustard operation but is superior in terms of significantly fewer arrhythmias and preservation of ventricular function, thus making it the procedure of choice for infants with TGA. |
| Yamaguchi 1990 (6) | 1982-1986 | Japan | English | Retrospective chart review | To describe experiences with ASO for TGA | ASO for TGA | 246/267 | The overall mortality rate was 35% in the first 3 years and 12% in the more recent 3 years suggestive of improvement of results with time era. |
| Pozzi 1990 (7) | 1984-1989 | Italy | Italian | Retrospective chart review | To describe experiences with ASO for TGA | ASO for TGA | 45/50 | They reported a 10% early mortality rate overall. |
| Wollenek 1991 (8) | 1984-1991 | Vienna/Austria | English | Cohort study | To describe experiences with ASO for TGA | ASO for TGA | 38/38 | In simple TGA, ASO can be performed with good short-term clinical results. The long-term benefit remains a matter of concern. |
| Castaneda 1991 (9) | 1983-1991 | Boston/USA | English | Retrospective chart review | To describe experiences with ASO for TGA | ASO for TGA | 505/505 | The ASO, as a primary operation, is our treatment of choice for neonates with TGA/IVS and TGA/VSD |
| Bellinger 1991 (10) | 1989 | USA | English | Observational/Cross sectional study | To explore cognitive development of children following early repair of TGA | ASO for TGA | 28/28 | Intraoperative events during corrective surgery for congenital heart disease may be associated with subsequent cognitive development. |
| Krian 1991(11) | 1985-1990 | Germany | English | Cohort study | To describe experiences with ASO for TGA | ASO for TGA | 60/87 | The early phase mortality risk after ASO is not significantly higher than after atrial corrective procedure |
| Mendoza 1991 (12) | 1983-1988 | USA | English | Cohort study | To determine the outcome in the first 33 infants who underwent ASO for TGA and to describe its effects on the growth and neurodevelopment of the survivors. | ASO for TGA | 33/33 | The majority of patients who undergo ASO demonstrate normal growth and development and no impairment of cardiovascular function. However, neurodevelopmental problems may occur. |
| Jatene 1992 (13) | 1975-1991 | Brazil | English | Cohort study | To evaluate the long-term follow-up of our patients submitted to the Jatene operation | ASO for TGA | 116/116 | The majority of the patients surviving 50 to 182 months are in good clinical condition and if dysfunctions are present these show no progression or severe hemodynamic alterations. |
| Kramer 1992 (14) | 1985-1990 | Germany | English | Cohort study | To describe experiences with ASO for TGA | ASO for TGA | 56/56 | ASO is the treatment of choice at present, for neonates with simple transposition. |
| Kirklin 1992 (15) | 1985-1989 | USA | English | Cohort study | To describe experiences with ASO for TGA | ASO for TGA | 513 | Good early and intermediate-term clinical outcomes can be obtained in neonates with simple transposition and transposition and ventricular septal defect by use of the ASO. |
| Tashiro 1992 (16) |  | Japan | Japanese | Cohort study | To describe ASO for TGA without use of prosthetic material | ASO for TGA | 5/5 | Results suggest excellent growth of the pulmonary artery after surgery. |
| Day 1992 (17) | 1987-1991 | USA | English | Observational/Cross sectional study | To determine whether coronary anatomy influences the outcome of the neonatal arterial switch operation | ASO for TGA | 70/70 | Coronary anatomy may influence surgical management and the postoperative course of newborn infants with transposition. |
| Binet 1992 (18) | 1984-1992 |  | French | Cohort study | To describe development of a technique for the complete correction of transposition of great vessels | ASO for TGA | 426/426 | Actuarial survival rates were: 89% for TGA-IVS at 5 years, 90% for TGA-VSD, 85.3% for TGA-VSD and coarctation at 3 years. |
| Castaneda 1993 (19) | 1983 - 1991 | USA | English | Cohort study | To describe experiences with ASO for TGA | ASO for TGA | 470/470 | The favorable early and midterm results of the ASO as a primary operation continue to make it the preferred approach for the neonate with TGA/IVS and TGA/VSD whenever possible. |
| Davis 1993 (20) | 1985-1990 | Australia | English | Case control study | To describe experiences with ASO for TGA in patients 21 days or older | ASO for TGA | 18/18 | The ASO can be safely carried out as a primary procedure for patients up to the age of 1 month and probably up to the age of 2 months |
| Serraf 1993 (21) | 1984-1992 | France | English | Retrospective chart review | To assess the results of the neonatal anatomic repair of transposition of the great arteries by a single institution | ASO for TGA | 432/432 | The arterial switch operation is feasible in almost all forms of transposition of the great arteries in neonates as primary and definitive repair |
| Ortega 1994 (22) | 1988-1992 | Spain | Spanish | Retrospective chart review | To assess the morbi-mortality in 12 patients undergoing Jatene's arterial repair for transposition of the great vessels | ASO for TGA | 12/12 | Jatene's technique is the best alternative for repair of transposition of the great vessels when there is no hypoplasia of the right cavity |
| Weindling 1994 (23) | 1983-1986 | USA | English | Observational/Cross sectional study | To determine the prevalence of myocardial perfusion abnormalities at rest and exercise and to assess exercise capacity in children after the arterial switch operation | ASO for TGA | 23/23 | Myocardial perfusion scan abnormalities assessed by technetium-99m sestamibi are common after an arterial switch operation |
| Kado 1994 (24) | 1993 | Japan | Japanese | Retrospective chart review | To assess reoperation rates after ASO for TGA | ASO for TGA | 162/162 | Actuarial survival and freedom from reoperation at 9 years were 90% and 69%, respectively. |
| Elkins 1994 (25) | 1985-1993 | USA | English | Retrospective chart review | To clarify the left ventricular performance and late functional results after AS0 for TGA | ASO for TGA | 53/53 | The arterial switch operation is associated with decreasing operative risk and excellent preservation of systemic ventricular function over the medium-term follow-up period |
| Garcia Hernandez 1995 (26) | 1988-1993 | Spain | Spanish | Retrospective chart review | To describe experiences with ASO for TGA | ASO for TGA | 21/21 | Our results encouraged to us to use the arterial switch operation in all children with simple transposition of the great arteries |
| Wernovsky 1995 (27) | 1983-1992 | USA | English | Retrospective chart review | To assess factors influencing early and late outcome of ASO for TGA | ASO for TGA | 470/470 | Both the mortality and reintervention risks are lower in patients with less complex anatomy. |
| Spiegelenberg 1995 (28) | 1977-1992 | Utrecht, Netherlands | English | Retrospective chart review | To assess the incidence and surgical treatment of postoperative pulmonary stenosis following ASO for TGA | ASO for TGA | 76/76 | The incidence of PS was not influenced by the type of reconstruction or the use of LeCompte’s maneuver |
| Bellinger 1995 (29) | 1988-1992 | USA | English | RCT | To compare the developmental and neurologic sequelae of two support strategies for ASO for TGA. | ASO for TGA | 151/151 | Heart surgery performed with circulatory arrest as the predominant support strategy is associated with a higher risk of delayed motor development and neurologic abnormalities at the age of one year than is surgery with low-flow bypass as the predominant support strategy |
| Serraf 1995 (30) | 1983-1994 | France | English | Retrospective chart review | To assess the causes and the results of reoperations following ASO for TGA, and to determine the existence of any risk factors. | ASO for TGA | 753/753 | Most late reoperations can be prevented by primary neonatal repair of almost all forms of transposition of the great arteries. |
| Luciani 1996 (31) | 1992-1995 |  | English | Cohort study | Preliminary clinical experience with neonates presenting with TGA/IVS | ASO for TGA | 45/45 | Our early results with 45 neonates undergoing this operative procedure continue to support this treatment modality |
| Bonnet 1996 (32) | 1991-1995 | France | English | Cohort study | To determine the prevalence of obstructions of the translocated coronary arteries following ASO for TGA | ASO for TGA | 78/78 | The prevalence of the late coronary artery complications after an arterial switch operation was low in this series |
| Bonhoeffer 1997 (33) | 1984-1993 | France | English | Cohort study | To describe a large series of coronary artery obstructions after the ASO for TGA and to discuss their clinical implications | ASO for TGA | 165/165 | Selective coronary angiography is the most accurate means to assess coronary artery obstruction after the arterial switch operation |
| Conte 1997 (34) | 1984-1994 |  | English | Retrospective chart review | To describe experiences with ASO for TGA | ASO for TGA | 707/707 | Results confirm ASO as the primary and definitive procedure in neonates with TGA. |
| Hovels-Gurich 1997 (35) | 1986-1992 | Germany | English | Observational/Cross sectional study | To evaluate the cardiological and general health status 3-9 years after neonatal ASO for TGA | ASO for TGA | 77/77 | The study confirms good midterm results after neonatal arterial switch operation for transposition with or without ventricular septal defect |
| Terada 1997 (36) | 1987-1997 | Japan | Japanese | Retrospective chart review | To review the surgical results for congenital cardiovascular anomalies in neonates | ASO for TGA | 157/234 | The survival rates through the arterial switch operation for d-TGA in 30 days is satisfactory (92%) |
| Tamisier 1997 (37) | 1987-1995 | France | French | Retrospective chart review | To determine the incidence of coronary events following neonatal arterial switch and to identify potential risk factors for death and coronary events | ASO for TGA | 236/236 | In most neonates, arterial switch operation carries a low operative risk and provides excellent mid-term results |
| Hovels-Gurich 1997 (38) | 1986-1992 | Germany | French | Observational/Cross sectional study | To assess the developmental status of children beyond 3 years of age after the neonatal ASO | ASO for TGA | 77/77 | The neonatal arterial switch operation with combined circulatory arrest and low-flow bypass is associated with neurologic as well as fine and gross motor impairment but appears to be well tolerated concerning cognitive functions |
| Foran 1998 (39) | 1990-1993 | England | English | Retrospective chart review | To assess the surgical outcome of the primary arterial switch operation (ASO) in infants 3 weeks to 2 months old | ASO for TGA | 193/193 | Primary ASO may be appropriate treatment for infants with TGA/IVS < or = 2 months old, regardless of preoperative echocardiographic variables. |
| Nogi 1998 (40) | 1986-1995 | Canada | English | Retrospective chart review | To determine the incidence, risk factors, and outcomes of acquired stenosis of the neopulmonary valve after the neonatal arterial switch operation. | ASO for TGA | 136/136 | Neopulmonary valve stenosis after the arterial switch operation is not uncommon and is associated with growth failure of the valve anulus often associated with supravalvular pulmonary stenosis |
| Aseervatham 1998 (41) | 1973-1994 |  | English | Retrospective chart review | A clinical comparison of arterial and atrial repairs for transposition of the great arteries | ASO for TGA | 51/103 | These early and midterm results suggest that the arterial switch operation has comparable overall mortality to, and less morbidity than, the atrial repairs for TGA. |
| Helvind 1998 (42) | 1993-1997 | Denmark | English | Retrospective chart review | To retrospectively examine a 4-year policy of restoring the morphologically left ventricle to the systemic circuit in patients presenting after 3 months of age with ventriculo-arterial discordance with or without associated atrio-ventricular discordance | ASO for TGA | 3/29 | Late anatomic correction of ventriculo-arterial discordance with or without atrio-ventricular discordance can be performed at a relatively low risk |
| Rappaport 1998 (43) | 1988-1992 | USA | English | RCT | To assess the relation of seizures after cardiac surgery in early infancy to neurodevelopmental outcome | ASO for TGA | 171/171 | n infants undergoing the arterial switch operation for correction of D-transposition of the great arteries, transient postoperative clinical and EEG seizures were associated with worse neurodevelopmental outcomes at ages 1 and 2 1/2 years as well as neurological and MRI abnormalities at 1 year of age |
| Haas 1999 (44) | 1983-1997 | Germany | English | Retrospective chart review | To assess long term results of ASO for TGA | ASO for TGA | 171/285 | The favorable long-term results demonstrate that the ASO can be considered as the optimal approach for patients with TGA |
| Pretre 1999 (45) |  | Switzerland | Other | Retrospective chart review | To describe experiences with ASO for TGA | ASO for TGA | 25/25 | The arterial switch operation is considered the procedure of choice for correction of transposition of the great arteries. The operation involves acceptable mortality and morbidity. |
| Bernuth 2000 (46) | 1983-1993 | Germany | English | Retrospective chart review | To evaluate the mid-term results after ASO for TGA | ASO for TGA | 181/188 | Results are encouraging, but prospective studies over a much longer follow-up time are necessary before definite conclusions can be drawn. |
| Armishaw 2000 (47) | 1995-1995 | New Zealand | English | Retrospective chart review | To assess the operative outcome, cardiac and neurodevelopmental sequelae in infants with TGA undergoing the ASO. | ASO for TGA | 48/48 | ASO is a relatively safe procedure with excellent cardiac and neurodevelopmental outcome in the majority of infants |
| Daebritz 2000 (48) | 1982-1997 | Germany | English | Retrospective chart review | To describe anatomical risk factors for mortality and cardiac morbidity after arterial switch operation | ASO for TGA | 299/312 | Malformations associated with complex transposition of the great arteries influence early and late mortality |
| Hutter 2001 (49) | 1977-2000 | Netherlands | Other | Cohort study | To examine the results of ASO for TGA | ASO for TGA | 195/198 | The long-term clinical outcome of the arterial switch operation was good |
| Pretre 2001 (50) | 1987-1999 | France |  | Retrospective chart review | To assess the value of analysing a large series of unselected cases of ASO fir TGA and formulate a realistic prognosis | ASO for TGA | 432/432 | The arterial switch operation in neonates achieves excellent results mid-term. These results allow anticipation of a favourable long-term prognosis. |
| Wetter 2001 (51) | 1984-2002 | Frankfurt, Germany | English | Retrospective chart review | To identify potential risk factors influencing early and late outcome ASO for TGA associated with ventricular septal defect including double-outlet right or left ventricle. | ASO for TGA | 77/105 | ASO associated with patch closure of ventricular septal defect can be performed early in life with a low risk of mortality (<5%), low incidence of reintervention (<15%) and promising long-term outcome |
| Hovels-Gurich 2001 (52) | 1994-1995 | Aachen, Germany | English | Cohort study | To assess neurodevelopmental outcome related to cerebral risk factors in children after neonatal arterial switch operation. | ASO for TGA | 33/65 | Neonatal arterial switch operation with combined circulatory arrest and low flow bypass is associated with neurological impairment, but not with reduced development. |
| Dunbaar-Masterson 2001 (53) | 1988-1992 | Boston USA | English | RCT | To study the long-term impact on general health status of D-transposition of the great arteries (D-TGA) after the arterial switch operation (ASO) during infancy | ASO for TGA | 155/155 | At age 8 years, children with D-TGA after ASO have an overall physical and psychosocial health status similar to that of the general population |
| Tsunezuka 2001 (54) | 1977-2000 | Utrecht, Netherlands | English | Retrospective chart review | To determine growth of the neo-aortic valve and the aortic anastomosis after ASO and the prevalence of insufficiency or stenosis | Other | 173/189 | After ASO the neo-aortic valve and sinus are larger than normal, |
| Kuroczynski 2001 (55) | 2001 | Mainz, Germany | German | Observational/Cross sectional study | To evaluate the incidence of postoperative pulmonary supravalvular stenosis in patients with d-TGA and to assess the rate of success or failure of balloon angioplasty | ASO for TGA | 67/70 | Balloon dilatation should be the first treatment in patients with pulmonary stenosis after ASO in TGA owing to the low complication rate and the potential benefit of this procedure |
| Brown 2001 (56) | 1986-1999 | Indiana USA | English | Retrospective chart review | Arterial switch operation: factors impacting survival in the current era. | ASO for TGA | 179/201 | The arterial switch operation for TGA has early low and late mortality and reoperation rates. Technical modifications in coronary reimplantation have minimized coronary artery pattern-related risks. |
| Losay 2001 (57) | 1982-1999 | Le Plessis-Robinson, France | English | Retrospective chart review | To describe late outcome after arterial switch operation for transposition of the great arteries. | ASO for TGA | 1121/1200 | Fifteen years after ASO, late mortality was low, with no deaths after 5 years; reoperation, mainly owing to pulmonary stenosis, occurred throughout the follow-up |
| Sharma 2002 (58) | 1991-2001 | New Delhi India | English | Retrospective chart review | To describe experiences with ASO for TGA | ASO for TGA | 271/299 | Excellent long-term results are obtained in operative survivors following the arterial switch operation. However. operative mortality remains a concern. |
| Al Qethamy 2002 (59) | 1995-2000 | Riyadh, Saudi Arabia | English | Retrospective chart review | To describe experiences with two stage arterial switch operation. | ASO for TGA | 49/49 | Late anatomic correction (> 6 months) after the preliminary procedure can be performed with an acceptable mortality and morbidity. |
| Sharma 2002 (60) | 1991-2001 | New Delhi, India | English | Retrospective chart review | To describe late outcome after arterial switch operation for complete transposition of great arteries with left ventricular outflow tract obstruction | Other | 23/299 | Presence of preoperative anatomical left ventricular outflow tract obstruction in patients undergoing arterial switch operation predicts high incidence of postoperative neoaortic regurgitation. |
| Gandhi 2002 (61) | 1985-2002 | Pittsburgh USA | English | Retrospective chart review | To highlight the frequency and nature of late invasive reintervention following ASO for TGA. | ASO for TGA | 144/144 | Invasive reintervention after the arterial switch operation is occasionally required. The most common indication is pulmonary stenosis. |
| Hovels-Gurich 2002 (62) | 1986-1992 | Aachen, Germany | English | Cohort study | To describe the neurodevelopmental status of children between 8 and 14 years of age after neonatal arterial switch operation for transposition of the great arteries | ASO for TGA | 96/96 | The neonatal arterial switch operation with combined circulatory arrest and low-flow bypass is associated increasingly with age, with reduced neurodevelopmental outcome but not with cognitive dysfunction. |
| Scheule 2002 (63) | 1983-1992 | Boston USA | English | Retrospective chart review | To evaluate the impact of coronary pattern on survival and reintervention in patients who underwent the arterial switch operation with a single coronary artery | ASO for TGA | 53/53 | The arterial switch operation with a single coronary artery can be performed safely irrespective of the coronary anatomy. |
| Hovels-Gurich 2002 (64) | 1986-1992 | Aachen Germany | English | Observational/Cross sectional study | To evaluate behavioural outcome and quality of life in children aged 8-14 years after neonatal arterial switch operation for transposition of the great arteries. | ASO for TGA | 60/60 | The neonatal arterial switch operation with combined circulatory arrest and low flow bypass is associated with parent reported long term behavioural impairment, but not with self-reported general reduction in quality of life. |
| Prifti 2002 (65) | 1990-2001 | Massa Italy | English | Retrospective chart review | To study the early and late outcome in terms of mortality, freedom from reoperation, predictors for late pulmonary stenosis and insufficiency of the neo-aortic valve in patients with TGA undergoing ASO. | ASO for TGA | 134/134 | ASO remains the procedure of choice for the treatment of various forms of TGA with acceptable early and later outcome in terms of overall survival and free reoperation |
| Hutter 2002 (66) | 1977-2000 | Utrecht, Netherlands | English | Retrospective chart review | This study examines the long-term outcomes of the arterial switch operation. | ASO for TGA | 195/195 | Long-term clinical outcome of the arterial switch operation is good, and perioperative mortality is now low |
| Tomizawa 2003 (67) | 1968-1999 | Japan | Japanese | Retrospective chart review | The aim of this study was to evaluate the late results of left ventricular aneurysm repair. |  | 1/86 | Patients who underwent revascularization, and were without major arrhythmias preoperatively, had better long-term survival |
| Legendre 2003 (68) | 1982-2001 | Le Plessis-Robinson, France | French | Retrospective chart review | To assess the prevalence of coronary lesions following ASO for TGA, and to evaluate the diagnostic methods to prevent their consequences | ASO for TGA | 1304/1304 | The prevalence of coronary lesions after ASO for TGA was 6.8% (95% CI 5 – 10). After the arterial switch procedure non-invasive investigations are not sensitive enough to diagnose coronary lesions and systematic coronary angiography and aortography should be performed. |
| Bellinger 2003 (69) | 1988-1992 | Boston USA | English | RCT | To determine which of the two major methods of vital organ support used in infant cardiac surgery, total circulatory arrest and low-flow cardiopulmonary bypass, results in better neurodevelopmental outcomes at school age. | ASO for TGA | 155/155 | Use of total circulatory arrest to support vital organs during heart surgery in infancy is generally associated with greater functional deficits than is use of low-flow cardiopulmonary bypass |
| Wiliam 2003 (70) | 1985-1989 | Toronto Canada | English | Cohort study | Too examine the intermediate outcomes and their associated risk factors in neonates undergoing ASO for TGA | ASO for TGA | 516/829 | Survival 15 years after TGA repair is good with most children functioning well, and results are best after an arterial switch operation |
| Rehnstrom 2003 (71) | 1982-2000 | Gothenburg, Sweden | English | Retrospective chart review | To determine medium-term outcome after arterial switch operation for complete transposition in a population-based cohort | ASO for TGA | 86/86 | medium-term survival is excellent, and the rate of reoperation and sequelae is low. |
| Hovels-Gurich 2003 (72) | 1986-1992 | Aachen Germany | English | Observational/Cross sectional study | To assess cardiac and general health status 8 to 14 years after neonatal arterial switch operation for transposition of the great arteries. | ASO for TGA | 96/96 | Good cardiac results persist 10 years after neonatal arterial switch operation for transposition of the great arteries. |
| Legendre 2003 (73) | 1982-2001 | Le Plessis-Robinson, France | English | Retrospective chart review | To assess the incidence and risk factors of coronary events after ASO | ASO for TGA | 1304/1304 | After ASO, coronary events are not rare, occurring most often early and are an important cause of death. |
| Formigari 2003 (74) | 1987-2001 | Rome, Italy | English | Retrospective chart review | To evaluate the prevalence and predictive factors associated with neoaortic valvular regurgitation in children who underwent ASO for TGA. | ASO for TGA | 173/173 | After arterial switch operation, there is an increasing frequency of neoaortic regurgitation, which may lead to significant valvular dysfunction later in life |
| Qing-yu Wu 2003 (75) | 2000-2002 | China | Chinese | Cohort study | To investigate the clinical efficacy of arterial switch operation on transposition of great artery (TGA) and Tausing-Bing anomaly. | ASO for TGA | 26/30 | The arterial switch procedure has a satisfying effect on TGA for patients older than 1 month with severe pulmonary hypertension |
| Bartlett 2004 (76) | 1988-2000 |  | English | Observational/Cross sectional study | To explore the impact of prenatal diagnosis on perinatal and perioperative variables and on outcomes at 1 year of age in patients with TGA | ASO for TGA | 346/364 | Infants with D-transposition of the great arteries with and without prenatal diagnosis differed with respect to perinatal and perioperative variables, but their development at 1 year of age was similar. |
| Mludzik 2004 (77) | 1991-1996 | Poland | Other | Observational/Cross sectional study | To analyse the long-term outcomes of ASO for TGA. | ASO for TGA | 100 | The most of patients after ASO for simple and complex forms of TGA, are asymptomatic Late complications are rare |
| Murthy 2004 (78) | 1998-2002 | Chennai, India | English | Cohort study | To describe a new technique to manage TGA with single coronary artery pattern. | ASO for TGA | 5/5 | This new coronary reallocation technique avoids problems related to coronary translocation such as traction and kinking. |
| Zhi-Wei Xu 2004 (79) | 2000-2003 | China | Chinese | Retrospective chart review | To summarize the clinical experience on the arterial switch operation for complex congenital heart disease in recent 3 years | ASO for TGA | 45/61 | The effect of arterial switch operation on the treatment of TGA was well accepted in this study |
| Kun-Lang Wu 2004 (80) | 2000-2002 | Taiwan | Chinese | Retrospective chart review | To describe the clinical outcome of patients undergoing ASO at our institution during the past 3 years |  | 44/44 | The ASO can be performed in infants with satisfactory results |
| Dibardino 2004 (81) | 1995-2003 | Houston Texas USA | English | Cohort study | To assess recent outcomes after the ASO in babies presenting with TGA and Taussig-Bing anomaly (TBA). | ASO for TGA | 116/125 | ASO can be performed safely and with a low incidence of need for reoperation on intermediate follow-up |
| Abid 2004 (82) | 1990-2003 | Tunisia | French | Retrospective chart review | To describe experiences with ASO for TGA |  | 62/62 | Results of anatomic repair are now excellent |
| Murthy 2004 (83) | 1995-1998 | Hyderabad India | English | Retrospective chart review | To describe midterm results of ASO without coronary translocation for TGA |  | 41/41 | This technique is a better alternative for surgeons who are not well versed with coronary translocation of conventional arterial switch operation and with difficult coronary anatomy. |
| Lafuente 2005 (84) | 1992-2003 | Argentina | Portuguese | Cohort study | To establish the short- and medium-term evolution of patients with TGA undergoing ASO | ASO for TGA | 113/122 | Clinical evolution in the medium term after anatomical correction (arterial switch) of transposition of the great vessels was excellent with 99% survival up to 11 years postoperative follow-up |
| Jia 2005 (85) | 2000-2003 | Shanghai, China | Chinese | Retrospective chart review | To report the surgical outcome of ASO for TGA with ventricular septal defect. | ASO for TGA | 6/6 | Arterial switch operation is of the first choice for transposition of the great arteries associated with ventricular septal defect |
| Pocar 2005 (86) | 1990-1998 | Paris, france | English | Retrospective chart review | To evaluate perioperative and late results after primary, single-stage arterial switch operation (ASO) associated with aortic arch obstruction repair | ASO for TGA | 38/38 | Infants with ventriculoarterial discordance and aortic arch obstruction represent a high-risk subgroup of candidates for an ASO |
| Freed 2006 (87) | 1996-2004 | Edmonton Canada | English | Cohort study | To report the surgical outcome of ASO for TGA |  | 88/88 | Transposition of great arteries, including complex types, can be corrected with low surgical risk and good intermediate survival; however, neurodevelopmental outcome is a concern. |
| Hwang 2006 (88) | 1991-2003 | Barcelona, Spain | English | Retrospective chart review | To determine the outcome of the neoaortic valve after the arterial switch operation for transposition of the great arteries | ASO for TGA | 140/140 | Neoaortic valve regurgitation progressed after the arterial switch operation. |
| Park 2006 (89) | 2003 | Seoul Korea | English | Observational/Cross sectional study | To evaluate the cerebral metabolism of TGA infants at birth and before ASO and neurodevelopment 1 year after ASO | ASO for TGA | 10/10 | Cerebral metabolism of infants with TGA was altered. The results at 1 year showed delayed mental and psychomotor development. |
| Marino 2006 (90) | 1984-1997 | Philadelphia USA | English | Cohort study | To assess the prevalence and progression, during childhood and adolescence of certain complications after the arterial switch operation. | ASO for TGA | 82/82 | At mid-term follow-up, significant neo-aortic valve regurgitation is present in 3.7%, and trivial to mild regurgitation in 81.4%; of patients |
| Prandstetter 2007 (91) | 1995-2005 | Linz, Austria | English | Retrospective chart review | To report the surgical outcome of ASO for TGA | ASO for TGA | 114/114 | The ASO can be performed safely and with low mortality and morbidity even in patients with complex TGA. |
| Roussin 2007 (92) | 1990-2003 | Le Plessis Robinson, France | English | Retrospective chart review | To evaluate early and midterm results in infants with TGA weighing less than 2,000 grams who underwent surgical procedure in the neonatal period. | Other | 25/25 | delaying repair in low birth weight neonates with simple or complex TGA does not confer any benefit and is associated with higher morbidity |
| Raisky 2007 (93) | 1987-2006 | Paris, France | English | Retrospective chart review | To evaluate the results of surgical revascularization in children with coronary artery lesions following neonatal ASO | ASO for TGA | 755/775 | Following ASO, coronary lesions are not uncommon, and they are progressive |
| Qamar 2007 (94) | 1999-2005 | Michigan USA | English | Retrospective chart review | To report the surgical outcome of ASO for TGA | ASO for TGA | 168/168 | The arterial switch operation can be performed with low mortality regardless of diagnosis or coronary pattern |
| Aoki 2008 (95) | 1991-2007 | Japan | Japanese | Retrospective chart review | To report the surgical outcome of ASO for TGA | ASO for TGA | 75/75 | Actuarial survival was 97%, and event-free rate was 83% at 16 years. |
| Nakano 2008 (96) | 1984-1997 | Japan | Japanese | Retrospective chart review | To report the surgical outcome of ASO for TGA | ASO for TGA | 202/202 | Actuarial survival was 90.6% at 10 years and 90.0% at 20 years |
| Angeli 2008 (97) | 1987-2007 | Paris, France | English | Retrospective chart review | To describe late reoperations after neonatal ASO for TGA | ASO for TGA | 803/803 | Late after neonatal ASO, the outcome in terms of survival and functional status is very satisfactory |
| Yamazaki 2008 (98) | 1975-1991 | Tokyo Japan | English | Retrospective chart review | To assess the cardiac outcome and risk factors for mortality of infants following the arterial switch operation (ASO). | ASO for TGA | 244 | Low early and late morbidity and mortality can be obtained in infants with TGA or double outlet right ventricle by definitive repair utilising the ASO |
| Wong 2008 (99) | 1984-1999 | Auckland, New Zealand | English | Retrospective chart review | To assess the cardiac outcome and risk factors for mortality of infants following the arterial switch operation (ASO). | ASO for TGA | 244/244 | Low early and late morbidity and mortality can be obtained in infants with TGA or double outlet right ventricle by definitive repair utilising the ASO. |
| Khan 2008 (100) | 1985-2001 | Riyadh, Saudi Arabia | English | Cohort study | To identify the prevalence of bicuspid pulmonary valve among patients with transposition of the great arteries undergoing the arterial switch operation | Other | 24/24 | Encountering a bicuspid pulmonary valve at the time of an arterial switch operation is not uncommon. |
| Jatene 2008 (101) | 1975-2000 | Sao Paulo Brazil | English | Cohort study | To study and analyze the prevalence of stenosis after ASO for TGA, | ASO for TGA | 553/553 | supravalvular pulmonary stenosis, post-Jatene operation for Transposition of Great Arteries, had a prevalence of 20.9% |
| Bove 2008 (102) | 1993-2006 | Ghent Belgium | English | Cohort study | Midterm assessment of the reconstructed arteries after the arterial switch operation. | Other | 93/93 | After arterial switch operation, the neoaortic root is usually enlarged, but with a growth pattern comparable to that of a normal population. |
| De Koning 2008 (103) | 1990-1995 | Rotterdam Netherlands | English | Observational/Cross sectional study | To investigate the cardiological health status and health-related quality of life after ASO for TGA | Other | 49/49 | At mid- to long-term follow-up after ASO, the occurrence of major events and re-intervention rate (6%) is low. |
| Nishino 2008 (104) | 1994-2007 | Japan | Japanese | Retrospective chart review | We describe the risk factors of coronary event after ASO for TGA | ASO for TGA | 44/44 | The risk factors of CE and the operative procedures are discussed |
| Rastan 2008 (105) | 1998-2006 | Leipzig, Germany | English | Cohort study | To evaluate the impact of moderate versus deep perioperative hypothermia on postoperative morbidity in patients receiving the arterial switch operation (ASO). | ASO for TGA | 100/100 | The ASO under full-flow moderate compared to deep hypothermia was advantageous regarding length of procedure and primary chest closure rate. |
| Shiraishi 2008 (106) | 1997 | Japan | Japanese | Retrospective chart review | To analyze the operative maneuver and long-term outcome of the arterial switch operation (ASO) for congenitally corrected TGA or double inlet left ventricle | Other | 8/9 | There was no early death, and 1 patient died 1 year after the operation due to chronic heart failure |
| Lange 2008 (107) | 1983-2006 | Munich Germany | English | Retrospective chart review | To describe the incidence and risk factors for aortic insufficiency and aortic valve replacement after ASO | ASO for TGA | 512/512 | The incidence of trivial or mild AI after the ASO is considerable and a progression over time is evident |
| Neufeld 2008 (108) | 1996-2003 | Edmonton Canada | English | Observational/Cross sectional study | To assess the 5-year neurocognition and health of an interprovincial inception cohort undergoing the arterial switch operation for transposition of the great arteries. | ASO for TGA | 65/65 | Most preschool children do well after surgical correction for transposition of the great arteries, including complex forms. |
| Moll 2009 (109) | 1991 |  | English | Retrospective chart review | To present our results ASO for TGA treatment in the long-term follow-up period (10–18 years) | ASO for TGA | 509 | ASO performed in newborns ensures favourable postoperative results and good child development |
| Vandekerckhove 2009 (110) | 1977-1989 | Leiden, Netherlands | English | Retrospective chart review | To analyze the size and function of aortic root and left ventricle as well as quality of life in patients 20 years after arterial switch procedure. | ASO for TGA | 39/39 | Clinical outcome is good 20 years after arterial switch operation and aortic valve function remains preserved in most patients. |
| Co 2009 (111) | 1984-2007 | Milwaukee USA | English | Retrospective chart review | To determine the prevalence and progression of neoaortic root dilation and neoaortic valve regurgitation in patients with TGA repaired with the ASO | ASO for TGA | 124/124 | Progressive neoaortic root dilation is common in patients with TGA after the ASO. Continued surveillance of this population is required. |
| Metton 2009 (112) | 1987-2008 | Paris, France | English | Retrospective chart review | To evaluate the impact of coronary patterns with intramural arteries on the outcome of ASO in neonates with TGA) | Other | 46/46 | Coronary patterns with intramural arteries remain associated with high coronary mortality and morbidity following neonatal ASO |
| Angeli 2009 (113) | 1991-2007 | Bologna, Italy | English | Retrospective chart review | To analyse the long-term patency of coronary arteries after neonatal arterial switch operation (ASO) | ASO for TGA | 119/119 | The late outcome in terms of survival and functional status after ASO is excellent. |
| Horer 2009 (114) | 1974-2006 | Munich Germany | English | Retrospective chart review | To compare survival, freedom from reoperation, and functional status between atrial switch and arterial switch operations for transposition of the great arteries. | ASO for TGA | 217/512 | Change from atrial to arterial switch led to improved long-term survival after hospital discharge but not to lower incidence of reoperation |
| Bohuta 2010 (115) | 2006-2008 | Kiev, Ukraine | English | Retrospective chart review | To evaluate our recent experience with ASO for TGA. | ASO for TGA | 188/188 | In the current era ASO can be safely performed for both TGA-IVS and TGA-VSD |
| Walter 2010 (116) | 1987-2008 | Berlin, Germany | English | Retrospective chart review | We evaluated the long-term outcome of aortic valve after ASO | ASO for TGA | 324/324 | Aortic regurgitation is not rare after ASO, but it is stable without progressive intensity |
| Furlanetto 2010 (117) | 1993-2008 | Developing Country | English | Retrospective chart review | We examined the immediate results of 176 consecutive arterial switch during last 15 years | ASO for TGA | 114/114 | After 173 consecutive arterial switch operations, improvement in the surgical result was possible. |
| Jhang 2010 (118) | 1997-2008 | Korea | English | Retrospective chart review | To evaluate the fate of neo aorta and the neo-aortic valve function | ASO for TGA | 216/239 | The prevalence of AI had an increasing tendency over time but significant AI and the need for reoperation are rare |
| Raja 2010 (119) | 2000-2009 | London, United Kingdom | English | Retrospective chart review | We report the mid-term follow-up of patients, who underwent arterial ASO for TGA | Other | 13 | Mid-term outcomes of ASO for a highly selected group of patients remain satisfactory, with a need for long-term follow-up. |
| Gorler 2010 (120) | 1973-2000 | Hannover, Germany | English | Retrospective chart review | We reviewed all patients that underwent surgical repair of TGA at our institution and compared long-term results after atrial and arterial switch operation. | Other | 80/316 | The arterial switch repair was associated with a higher early mortality whereas long-term survival was comparable between both groups. |
| Ismail 2010 (121) | 2002-2008 | Riyadh, Saudi Arabia | English | Retrospective chart review | To assess the early postoperative course and outcome for children with TGA/IVS and still conditioned left ventricle presenting for late primary ASO. | ASO for TGA | 91/91 | Provided the LV still is conditioned, age should not be a limitation for arterial switch operation. |
| Tobler 2010 (122) | 2000-2009 | Toronto Canada and Massachusetts USA | English | Retrospective chart review | We sought to describe cardiac outcomes during pregnancy in women with TGA who had undergone an ASO in childhood. | Other | 74/74 | young women with TGA from this early cohort repaired with ASO are reaching child-bearing age. A significant proportion have residua and/or sequelae that can confer risk for adverse cardiac events in pregnancy. |
| Park 2010 (123) | 1996-2008 | Seoul, Republic of Korea | English | Retrospective chart review | The aim of this study is to evaluate the significance of pulmonary annulus size with the outcome after the arterial switch operation. | ASO for TGA | 9/9 | It was possible to extend the indication for the arterial switch operation with acceptable outcome to the patient with a Z-score of about -3 of the pulmonary annulus despite bicuspid pulmonary valve. |
| Liu 2010 (124) | 2000-2008 | China | English | Retrospective chart review | Midterm results of arterial switch operation in older patients with severe pulmonary hypertension. | Other | 86/86 | Midterm results of the arterial switch operation for patients older than 6 months are excellent in the current era. |
| Choi 2010 (125) | 1987-2004 | Seoul, Korea Republic | English | Retrospective chart review | Our study aimed to examine long-term outcomes after ASO for simple complete transposition of the great arteries (TGA). | ASO for TGA | 108/108 | Although most patients showed normal physical growth and development after successful ASO, meticulous long-term follow-up is necessary because of progressive AI and coronary complications. |
| ying Long 2010 (126) | 2000-2008 | Beijing, China | English | Retrospective chart review | Safety and efficacy of arterial switch operation in previously inoperable patients. | Other | 86/86 | ASO is safe and effective in these selected subset patients. |
| Tobler 2010 (127) | 1991 | Toronto, Canada | English | Retrospective chart review | We sought to determine cardiac outcomes in young adults with complete transposition of the great arteries (TGA) after the arterial switch operation (ASO). | ASO for TGA | 132/132 | Although most adults after ASO are well, and few have residual defects, there are subgroups, particularly those who needed further cardiac intervention in childhood, who are at higher risk for ventricular and valve dysfunction and arrhythmias. |
| Ismail 2010 (128) | 2002-2008 | Riyadh, Saudi Arabia | English | Retrospective chart review | This study aimed to assess the early postoperative course and outcome for children with TGA/IVS and still conditioned LV presenting for late primary ASO | ASO for TGA | 91/91 | For patients with TGA/IVS, ASO still can be tolerated beyond the first month of life in selected cases. |
| El-Segaier 2010 (129) | 1980-2007 | Lund, Sweden | English | Observational/Cross sectional study | To report the late coronary complications and their treatment after arterial switch operation | ASO for TGA | 279/279 | Asymptomatic patients with an uneventful course after ASO may have coronary obstruction. |
| Metton 2010 (130) | 1987-2008 | Paris. France | English | Retrospective chart review | To evaluate the impact of coronary patterns with intramural arteries on the outcome of arterial switch operation (ASO) in neonates with transposition of the great arteries (TGA) | Other | 46/46 | Coronary patterns with intramural arteries remain associated with high coronary mortality and morbidity following neonatal ASO, even in the current era. |
| Chiu 2010 (131) | 1998-2008 | Taipei, Taiwan | English | Retrospective chart review | We aim to restore the natural spiral great arteries and compare such ASO with conventional ASO with the lecompte manoeuvre to clarify the functional implications of the spiral great arteries. | ASO for TGA | 130/130 | Intermediate-term results of spiral ASO were satisfactory. |
| Michalak 2010 (132) | 1991-2008 |  | English | Retrospective chart review | Neoaortic valve function 10 to 18 years after arterial switch operation. | ASO for TGA | 161/161 | Neoaortic regurgitation occurrence increases constantly over time after ASO. |
| Nakano 2011 (133) | 1984-2010 | Japan | English | Retrospective chart review | We reviewed our 28 years of experience of arterial switch operation (ASO) for transposition of the great arteries to investigate late sequelae of this procedure. | ASO for TGA | 387/387 | ASO was performed with satisfactory results in the overall survival and functional status. |
| Kaldararova 2011 (134) | Bratislava, Slovakia |  | English | Observational/Cross sectional study | The aim of the study was to compare long-term neo-aortic growth/function in these patients. | ASO for TGA | 85/133 | Proximal neo-aortic dilatation is significantly greater  in patients after ROSS compared to ASO. |
| Michalak 2011 (135) |  | Lodz, Poland | English | Retrospective chart review | This study was aimed at assessing aortic root diameters in long term observation after arterial switch | Other | 172/172 | There were no significant changes in the aortic root diameters between the groups compared |
| Akdeniz 2011 (136) | 2007-2010 | Turkey | Turkish | Retrospective chart review | Follow-up data of patients with simple transposition of great arteries (TGA) and TGA with ventricular septal defect (VSD), who had arterial switch operation (ASO) are compared. | ASO for TGA | 76/76 | Arterial switch operation provides anatomical correction in TGA |
| Raja 2011 (137) | 2000-2009 | London, UK | English | Retrospective chart review | We report the mid-term follow-up of patients, who underwent arterial switch operation (ASO) for transposition of the great arteries | Other | 13/13 | Mid-term outcomes of ASO for a highly selected group of patients remain satisfactory, with a need for long-term follow-up. |
| Calderon 2011 (138) | 2003-2005 | Paris, France | English | Cohort study | To assess the effect of prenatal diagnosis of congenital heart disease on neurocognitive outcomes in children with d-transposition of the great arteries (TGA) after surgical correction | ASO for TGA | 90/90 | Prenatal diagnosis of TGA is associated with better neurocognitive outcomes. |
| Mussa 2011 (139) | 1988-2010 | Birmingham, UK | English | Cohort study | This study sought to investigate the impact of surgical timing on early postoperative morbidity. | ASO for TGA | 241/241 | Timing of surgical repair does not seem to influence the rate of these complications. |
| Sterrett 2011 (140) | 1977-2007 | Leiden, Netherlands | English | Retrospective chart review | The purpose of our study is to examine mid- to-late-term myocardial perfusion at peak exercise in SWITCH patients | ASO for TGA | 42/84 | This study demonstrates that a small percentage (5%) of patients an average of 12 years after SWITCH have abnormal myocardial perfusion at peak exercise. |
| Ahmed 2011 (141) | 1990-2010 | Belfast Ireland | English | Retrospective chart review | We have reviewed our experience with anatomic correction of TGA, with direct pulmonary artery anastomosis | ASO for TGA | 101/101 | The arterial switch repair by direct anastomosis, retaining a normal position of the pulmonary bifurcation is feasible with an acceptable mortality and a low incidence of significant pulmonary artery |
| Gorler 2011 (142) | 1973-2000 | Hannover Germany | English | Retrospective chart review | Long-term morbidity and quality of life after surgical repair of transposition of the great arteries: atrial versus arterial switch operation | ASO for TGA | 80/302 | Although long-term survival was not significantly different after atrial and arterial switch repair, we observed an increased morbidity in the atrial switch group during late follow-up. |
| Edwin 2011 (143) | 2006-2009 | Johannesburg, South Africa | English | Retrospective chart review | Late primary arterial switch for transposition of the great arteries with intact ventricular septum in an African population. | ASO for TGA | 22/22 | The age limit for the primary ASO can be extended to at least 10 weeks; temporary mechanical circulatory support may be required as a rescue. |
| Jacobs 2011 (144) | 2005-2009 | Florida USA | English | Retrospective chart review | Patterns of Practice and Outcomes from the Congenital Heart Surgery Database of the Society of Thoracic Surgeons | ASO for TGA | 1669/3258 | Although surgery for TGA is often complex and may be associated with morbidity, most patients survive without major complications. |
| Kim 2011 (145) | 1994-2010 | Busan, South Korea | English | Retrospective chart review | We reviewed the morphological characteristics and evaluated the early and mid-term results of ASO for patients with an intramural coronary artery. | ASO for TGA | 144/158 | The arterial switch operation in Transposition of Great Arteries or Double Outlet Right Ventricle patients with intramural coronary can be performed with low mortality; however, there is a high incidence of intraoperative or postoperative coronary problems. |
| Leon-Wyss 2011 (146) | 1997-2009 | Antigua, Guatemala | English | Retrospective chart review | In this study, we reviewed our 13-year experience with TGA repair | ASO for TGA | 51/79 | During this 13-year period, a low volume of surgery for transposition of the great arteries (TGA) was performed at our institution with a relatively high surgical mortality |
| Delmo Walter 2011 (147) | 1987-2010 | Berlin, Germany | English | Retrospective chart review | We investigated the onset, incidence, and outcome of pulmonary stenosis after arterial switch operation in neonates with transposition of the great arteries and intact ventricular septum. | ASO for TGA | 174 | Over time, pulmonary stenosis developed after arterial switch operation. |
| Rudra 2011 (148) | 1983-2007 | Chicago USA | English | Retrospective chart review | This study investigates overall mortality and factors for neopulmonary artery, neoaortic, and coronary artery surgical reintervention. | ASO for TGA | 258/258 | Arterial switch operation mortality has decreased. |
| Moll 2012 (149) | 1992-2009 | Lodz, Poland | English | Retrospective chart review | This research was to assess the efficacy of ASO based on the incidence of postprocedural supravalvular neopulmonary stenosis | ASO for TGA | 346/346 | Direct neopulmonary artery anastomosis during arterial switch is an interesting alternative to patch reconstructions and ensures a good postoperative result with low rates of complications |
| Camarda 2012 (150) | 1990-2011 |  | English | Retrospective chart review | Anatomic mitral valve anomalies in d-transposition of the great arteries | ASO for TGA | 218/218 | Mitral valve anomalies associated with d-TGA are rare but present with remarkably consistent anatomic features and a higher risk of coarctation. |
| Michalak 2012 (151) | 1992-2011 | Lodz Poland | English | Retrospective chart review | To assess the long-term outcomes after switch operation performed in neonatal period. | ASO for TGA | 611/611 | Arterial switch operation performed in neonatal period ensures good postoperative effect |
| Baruteau 2012 (152) | 1982-2011 | Paris, France | English | Cohort study | This prospective study evaluates the status of survivors of ASO for TGA at adult age | Other | 103/103 | Close to 20 years after ASO for complex TGA, late outcome was encouraging with no death after the first year of life. |
| Li, 2012 (153) | 1999-2011 | Peking, China | English | Retrospective chart review | We reviewed our 12 years ‘experience of arterial switch operation (ASO) for transposition of the great arteries to investigate short-term result of this procedure | ASO for TGA | 423 | Simple ASO was performed with satisfied results in overall survival and functional status. |
| Stoica 2012 (154) | 2003-2011 | Bristol UK | English | Retrospective chart review | We examined morbidity, which remains significant, particularly for complex ASO. | ASO for TGA | 101 | In this consecutive series without operative mortality, morbidity was significantly higher in complex ASO |
| Huang 2012 (155) | 2000-2008 | Guangxi Zhuang China | English | Cohort study | The goal of the study was to evaluate the quality of life of children after the older corrective arterial switch operation (ASO) | ASO for TGA | 86/86 | Patients who underwent a corrective ASO at an older age showed acceptable scores for all scales, and they were all comparable with those of a healthy population |
| Fricke 2012 (156) | 1983-2009 | Melbourne Australia | English | Retrospective chart review | To determine the long-term outcomes for patients after ASO performed at a single institution over a 25-year period. | ASO for TGA | 618/618 | The ASO can be performed with good long-term results. |
| Popov 2012 (157) | 1991-1999 | Gottingen, Germany | English | Retrospective chart review | To evaluate operative risk and midterm outcome in patients who underwent ASO for TGA at our centre. | ASO for TGA | 52/52 | ASO remains the procedure of choice for TGA with acceptable early and late outcome in terms of overall survival and freedom of reoperation. |
| Andropoulos 2012 (158) | 2006-2010 | Houston Texas USA | English | Cohort study | To describes neurodevelopmental outcomes at age 12 months after neonatal ASO | ASO for TGA | 30/30 | At 12 months after ASO, neurodevelopmental outcome means were within normal population ranges. |
| Oda 2012 (159) | 1984-2010 | Fukuoka, Japan | English | Retrospective chart review | We reviewed our 28 years of experience of arterial switch operation (ASO) for transposition of the great arteries to investigate late sequelae of this procedure. | ASO for TGA | 387/387 | ASO was performed with satisfactory results in the overall survival and functional status. |
| Ma 2012 (160) | 1999-2011 |  | English | Retrospective chart review | To evaluate the early and midterm outcomes of arterial switch operation in transposition of the great arteries, unrestrictive ventricular septal defect, and pulmonary arterial hypertension. | ASO for TGA | 84/84 | The arterial switch operation significantly improved the quality of life and possibly life expectancy in patients with transposition of the great arteries, unrestrictive ventricular septal defect, and pulmonary arterial hypertension. |
| Angelli 2012 (161) | 1987-2010 | Paris, France | English | Retrospective chart review | Long-term evaluation of the impact of bicuspid pulmonary valve on neoaortic valve regurgitation and aortic root dilatation after ASO for TGA | Other | 40/40 | ASO is a safe option for TGA associated with a well-functioning bicuspid pulmonary valve with low morbidity and mortality. |
| Becker 2013 (162) | 1992-2012 | Santiago, Chile | Spanish | Retrospective chart review | To report the results of the arterial Switch operation in patients with D-transposition of the great vessels (D-TGA) and to evaluate their late course. | ASO for TGA | 108/108 | Surgical mortality for the arterial switch operation in patients with D-TGA has significantly decreased along a 20-year period. |
| Ruys 2013 (163) | 1985-2009 | Rotterdam, Netherlands | English | Cohort study | To describe the long-term cardiological and psychological results of our first surgical cohort of arterial switch operation (ASO) patients and compare the results with our earlier series of Mustard patients | ASO for TGA | 30/30 | The progression made in surgical treatment for transposition of the great arteries from Mustard to ASO has had a positive impact on survival, cardiac function, exercise capacity, and also self-reported quality of life and somatic complaints. |
| Boukhris 2013 (164) | 2013 | Tunis, Tunisia | English | Observational/Cross sectional study | To assess the long-term results of ASO in Tunisian children with TGA and to identify potential factors affecting these results. | Other | 44/44 | The TGA, including complex types, can be corrected with good long-term outcomes by ASO. |
| Van Der Bom 2014 (165) | 1977-1994 | Amsterdam Netherlands | English | Retrospective chart review | To assess the change in neo-aortic dimensions, prevalence of neo-aortic dilation >40 mm and long-term outcome in adults who underwent ASO in childhood. | ASO for TGA | 116/116 | In early adulthood, neo-aortic growth was on average linear and did not stabilise over time. |
| A, Ng 2013 (166) |  | Melbourne Australia | English | Retrospective chart review | To determine the cardiac outcomes in adults with complete transposition of the great arteries (TGA) after arterial switch operation (ASO) | ASO for TGA | 49/49 | Majority of young adults post ASO are functionally well with a small subset requiring intervention in adulthood |
| Driessen 2013 (167) | 2013 | Utrecht, Netherlands | English | Cohort study | This study evaluates the presence of coronary obstruction and myocardial infarction or left ventricular (LV) dysfunction using multimodality imaging in patients decades after ASO. | ASO for TGA | 32/32 | the current study found no coronary pathology using computed tomography angiography in patients up to 33 years after ASO. |
| Arcieri 2013 (168) | 2000-2013 | Italy | Italian | Retrospective chart review |  | ASO for TGA | 140 |  |
| Khairy 2013 (169) | 1983-1999 | Boston, USA | English | Retrospective chart review | To evaluate Cardiovascular outcomes after the arterial switch operation for D-transposition of the great arteries | ASO for TGA | 400 | Long-term and arrhythmia-free survival is excellent after arterial switch operation. |
| Xing Zhang 2013 (170) | 2008 | Beijing, China | English | Retrospective chart review |  | ASO for TGA | 180 |  |
| Lim 2013 (171) | 1987-2011 | Seoul, Korea Republic | English | Retrospective chart review | To assess Long-term results of the arterial switch operation for ventriculo-arterial discordance. | ASO for TGA | 241 | The survival and functional outcomes of the ASO were excellent in the long-term. |
| Kalfa 2013 (172) | 1986-2011 | Le Plesis Robinson, France | English | Retrospective chart review | To determine long-term outcomes and prognostic factors in this specific population with left outflow tract obstruction | Other | 55/55 | Long-term outcomes of ASO for patients with TGA and anatomic left ventricular outflow tract obstruction are satisfactory in selected patients. |
| Walsh 2013 (173) | 1999-2011 | New York, USA | English | Retrospective chart review | To evaluate the long-term outcomes of d-TGA patients with a bicuspid pulmonary valve who underwent ASO. | Other | 27/343 | d-TGA patients with bicuspid pulmonary valve who undergo ASO are at low risk for morbidity related to these abnormal bicuspid pulmonary valve functioning in the neo-aortic position. |
| Starling 2013 (174) | 2005-2011 | London, UK | English |  | To determine rates of pulmonary artery (PA) reintervention following arterial switch operation (ASO) for transposition of the great arteries (TGA) and to identify factors associated with reintervention | ASO for TGA | 126/126 | Pulmonary artery stenosis requires reintervention in 9% of patients following ASO, most frequently within the first postoperative year |
| Michalak 2013 (175) | 1992-2011 | Lodz, Poland | English | Retrospective chart review | To assess the neoaortic root diameter and relative proportion in children with TGA after surgical correction and to identify possible correlations with the development of neoaortic insufficiency. | Other | 172/611 | The neoaortic root in children after the arterial switch procedure develops differently from that in healthy children, but this is not evidently related to Neo aortic root development or associated heart defects. |
| Liu 2013 (176) | 2010-2011 | Beijing, China | English | Retrospective chart review | To describe ASO surgery in children older than three years old. | ASO for TGA | 41 | Children older than three years old can still undergo the ASO procedure, but residual pulmonary hypertension is present. |
| Amoozgar 2013 (177) | 2005-2011 | Shiraz, Iran | English | Retrospective chart review | To evaluate the myocardial and valvular function at midterm postoperative follow-up in patients with ASO for TGA. | ASO for TGA | 33/33 | This study showed good left ventricular function, but some abnormalities in lateral tricuspid tissue Doppler velocities. |
| Cain 2013 (178) | 2000-2011 | Milwaukee, USA |  | Retrospective chart review | This study evaluates the relationship of morbidity and resource utilization with the timing of early neonatal repair of transposition of the great arteries and intact ventricular septum | Other | 70/70 | Earlier repair of transposition of the great arteries and intact ventricular septum was associated with decreased resource utilization and no detriment to clinical outcomes. |
| Michalak 2014 (179) | 1991-2013 | Lodz, Poland | English | Retrospective chart review | To establish the frequency of significant supra ventricular pulmonary stenosis in patients with TGA after arterial switch procedure, | ASO for TGA | 665/655 | Although the risk of significant supra ventricular pulmonary stenosis after arterial switch operation is relatively low, relief of supravalvular pulmonary stenosis represents about half of indications for postoperative reinterventions. |
| Bisoi 2014 (180) | 2003-2010 | New Delhi, India | English | Cohort study | To report the midterm results of children with TGA-IVS older than six weeks undergoing primary ASO. | Other | 109/109 | Primary ASO can be safely performed in children with regressed ventricle, irrespective of age with encouraging results. |
| Driessen 2014 (181) | 2014 |  | English | Cohort study | The current study investigates coronary obstruction and anatomic characteristics of the coronaries in patients after ASO compared to controls. | ASO for TGA | 58/58 | Coronary arteries in patients after ASO take off from the aortic root at a higher level and show small but significant differences in cross sectional areas compared to healthy controls. |
| Gil 2014 (182) | 1977-2014 | Madrid, Spain | English | Retrospective chart review | To assess the incidence and sensitivity of magnetic resonance imaging (MRI) in the diagnosis of the coronary obstruction after ASO | ASO for TGA | 157/157 | After arterial Switch operation coronary events are not rare. |
| Parezanovic 2014 (183) | 2005-2009 | Serbia | Other | Retrospective chart review | To estimate the success rate of ASO through retrospective analysis of mortality and late complications. | ASO for TGA | 57/57 | Arterial switch operation has been successfully performed at our institution, with acceptable perioperative mortality and excellent late outcome |
| Heinrichs 2014 (184) | 1986-1992 | Aachen, Germany | English | Observational/Cross sectional study | We studied brain structure abnormalities in adolescents and young adults who had undergone the neonatal arterial switch operation for transposition of the great arteries and related them to the neurologic and psycho-intellectual outcomes | ASO for TGA | 60/60 | Despite encouraging overall neurodevelopmental outcomes, a significant minority had performances below the expected level, emphasizing the need for ongoing surveillance. |
| Bozicnik 2014 (185) | 2000-2013 | Padua, Italy | English | Observational/Cross sectional study | To detect late complications of ASO. | ASO for TGA | 79 | Patients treated with ASO are asymptomatic despite the relative high incidence of coronary anomalies or pulmonary branches stenosis. |
| Praetere 2014 (186) | 1991-2009 |  | English | Retrospective chart review | To assess the evolution of supravalvular pulmonary stenosis following ASO for TGA over time | ASO for TGA | 133/133 | ASO shows excellent long-term results in simple TGA with a very low morbidity and mortality and is therefore the procedure of choice. Re-intervention rate is determined by supravalvular pulmonary stenosis. |
| Rodriguez 2014 (187) | 1985-2010 | Sevilla, Spain | English | Retrospective chart review | To determine the mid-term results of ASO for TGA | ASO for TGA | 155/155 | Mid-term survival of patients after arterial switch operation is excellent and their functional status is good. |
| Michalak 2015 (188) | 1991-2014 | Lodz, Poland | English | Retrospective chart review | To establish occurrence and the most frequent reasons for reoperations and reinterventions in patients with TGA after ASO | ASO for TGA | 690/690 | Frequency of reoperation and reintervention in patients with TGA after ASO remains low |
| Ng Benton 2015 (189) | 1990-2014 | Iowa, USA | English | Retrospective chart review | Our study analyzes longitudinal echocardiography data focused on the assessment of supravalvular pulmonary stenosis after ASO performed at a single institution. | ASO for TGA | 103/103 | The largest drop off in freedom from a peak gradient ≥60 mmHg occurs in the first year, with a slower continued progression over the next 10 years. |
| Van Wijk 2015 (190) | 1978-2013 | Utrecht, Netherlands | English | Retrospective chart review | To evaluate a possible relation between the surgical era and the need for future re-intervention | ASO for TGA | 285 | The risk of re-intervention after ASO is inversely related to the surgical era. |
| Nittur 2015 (191) | 2000-2015 | Wales, United Kingdom | English | Retrospective chart review | To describe 15 years experiences with ASO for TGA following incorporation of outflow tract views in antenatal scans. | ASO for TGA | 74/74 | Inclusion of outflow tract view has led to substantial improvement in antenatal detection of TGA. |
| Tsuda 2015 (192) | 1998-2013 | Philadelphia, USA | English | Retrospective chart review | To investigate coronary artery morphology in 28 patients with uneventful initial postoperative course | ASO for TGA | 28/28 | We advocate that all patients after ASO warrant routine coronary image studies during late childhood before participating in competitive sports. |
| Noris 2015 (193) | 1990-1998 | Mallorca, Spain | English | Retrospective chart review | To determine cardiac outcomes and prevalence of coronary stenosis in adults with TGA after ASO | ASO for TGA | 62/62 | Long term outcomes of patients with TGA who survive arterial switch repair are good in terms of mortality and functional class |
| Yildiz 2015 (194) | 2010-2014 | Istanbul, Turkey | English | Retrospective chart review | S to assess the surgical outcome of the primary ASO in children with TGA-IVS presenting beyond 3 weeks of age | ASO for TGA | 12 | The primary ASO for patients with TGA/IVS still can be tolerated beyond the third week of life. |
| Manso 2015 (195) | 1995-2014 | Ribeirao Preto Brazil | English | Retrospective chart review | To describe the outcomes in patients submitted to arterial switch operation and to analyze the predictors of in-hospital mortality and further need of re-operation at a single-center institution. | ASO for TGA | 128/128 | Arterial switch operation remains the procedure of choice in patients with transposition of great arteries. |
| Lo rito 2015 (196) | 1988-1998 | Birmingham, UK | English | Retrospective chart review | To evaluate long-term performance of the aortic valve after an arterial switch operation (ASO), in terms of regurgitation and reoperation. | ASO for TGA | 332/332 | Reoperation on the aortic valve is rarely necessary, even late after an ASO |
| Michalak 2015 (197) | 1991-2014 | Lodz, Poland | English | Retrospective chart review | The aim of this study was to establish occurrence of the coronary anomalies in patients with TGA after arterial switch operation and their impact on long term follow up including coronary complications | ASO for TGA | 690/690 | Coronary anomalies in children with TGA are common finding and occur in about 1/3 of patients/128 |
| Jenkinson 2015 (198) | 2008 | Perth, Australia | English | Cohort study | To the results of the Arterial Switch Operation undertaken in a small paediatric cardiac surgical unit, with all cases operated by a single surgeon. | ASO for TGA | 44/44 | The Arterial Switch Operation presents many challenges to the cardiac surgical team. |
| Gardner 2015 (199) | 2001-2014 | Liverpool UK | English | Retrospective chart review | To establish whether local improvement in prenatal diagnosis of TGA improved early survival rates. | ASO for TGA | 228/228 | Improving rates of prenatal diagnosis of ASO does not currently seem to be reflected in an increased rate of survival. |
| Sun 2015 (200) | 2004-2012 | Hangzhou, China | English | Cohort study | To evaluate the new method for the reconstruction of the pulmonary artery in arterial switch operation (ASO). | ASO for TGA | 108/108 | No pulmonary stenosis was detected with the simplified Bernoulli formula. |
| Tsuda 2015 (201) | 1998-2013 | Wilmington USA | English | Retrospective chart review | To determine the incidence of late coronary artery abnormalities after arterial switch operation (ASO) | ASO for TGA | 40/40 | Incidence of late coronary stenosis or occlusion was not infrequent after ASO (11.3%) and presented usually without preceding symptoms and often after negative non-invasive screening. |
| Michalak 2016 (202) | 1991-2015 | Lodz, Poland | English | Retrospective chart review | To establish the frequency of bicuspid native pulmonary valve in patients with TGA and correlate its presence with major postoperative complications | ASO for TGA | 716/716 | Bicuspid neoaortic valve did not increase the risk of postoperative complications |
| Villalba 2016 (203) | 2016 | Caba, Argentina | Portuguese | Retrospective chart review | To evaluate the mid- and long-term outcome of ASO for TGA. | ASO for TGA | 224/224 | The arterial switch operation has excellent long-term survival. |
| Baruteau 2016 (204) | 1982-1998 | Paris, France | English | Retrospective chart review | To evaluate long-term outcomes after the arterial switch operation (ASO) for complex transposition of the great arteries (TGA) |  | 220/220 | Despite a continual rate of reinterventions, long-term survival and cardiovascular outcome are excellent after ASO for complex TGA. |
| Gonzalez-Lopez 2016 (205) | 2006-2015 | Madrid, Spain | English | Retrospective chart review | To analyse the outcomes and morbi-mortality trends following ASO for TGA | ASO for TGA | 115 | Early outcomes following ASO have improved in recent years. |
| Fricke 2017 (206) | 1983-2014 | Melbourne, Australia | English | Retrospective chart review | To determine outcomes of patients who underwent arterial switch operation (ASO) with concomitant aortic arch obstruction repair as a single stage procedure. | Other | 35/666 | Patients who go undergo ASO with associated aortic arch obstruction as a single stage repair have good early outcomes. |
| Cleuziou 2016 (207) | 1983-2014 | Munich, Germany | English | Retrospective chart review | To analyse the long-term outcome of this subgroup of patients with aortic arch anomalies undergoing ASO for TGA. | ASO for TGA | 207/27 | Long-term outcome after the ASO for TGA-VSD are excellent, but major reoperations are necessary in about 11% of the patients. |
| Hauet 2016 (208) | 2008-2014 | Angers, France | English | Retrospective chart review | To evaluate the impact of preoperative management on in-hospital post-operative outcomes after an ASO for TGA | ASO for TGA | 59/59 | that younger age at surgery is associated with lower postoperative morbidity in newborns undergoing an ASO. |
| Fricke 2016 (209) | 1983-2009 | Melbourne, Australia | English | Retrospective chart review | To determine the outcome of adult survivors who underwent ASO for TGA in childhood. | ASO for TGA | 637/692 | Adult survivors of the ASO have good long-term outcomes. |
| Bokenkamp 2016 (210) | 1977-2015 | Leiden, Netherlands | English | Retrospective chart review | To identify predictors of reoperation for right ventricular outflow tract obstruction in patients who underwent ASO) and arch repair for TGA or Taussig-Bing anomaly with aortic arch obstruction | ASO for TGA | 45 | Taussig-Bing anomaly and smaller preoperative aortic annulus diameter (Z-score) were significant predictors of reoperation for RVOTO in patients after ASO for TGA or Taussig-Bing anomaly with aortic arch obstruction |
| Shim 2016 (211) | 1995-2014 | Seoul, Korea | English | Retrospective chart review | To review the ASO at a single institution with a small volume. | ASO for TGA | 139 | ASO can be performed with good early results and favorable long-term outcomes even in a small-volume center |
| Ma 2016 (212) | 2003-2013 | Beijing, China | English | Retrospective chart review | To evaluate aortic valve performance in TGA patients who had an ASO |  | 583/614 | After an arterial switch operation, we report a favorable incidence of neoaortic valve regurgitation and rare neoaortic valve replacement. |
| Erek 2016 (213) | 2010-2015 | Istanbul, Turkey | English | Observational/Cross sectional study | To compare the outcome of arterial switch operation for Taussig Bing anomaly versus transposition of the great arteries and ventricular septal defect | ASO for TGA | 22/58 | Although the incidence of aortic arch anomalies is higher in Taussig-Bing Anomaly group, early and intermediate term outcomes are similar with TGA+ VSD group |
| Hicks 2016 (214) | 2004-2010 | Edmonton Canada | English | Cohort study | To describe the early neurocognitive and language outcomes for children undergoing ASO in a tertiary center | ASO for TGA | 91/91 | Following ASO for TGA, children have cognitive and motor skills similar to population norms |
| Nellis 2016 (215) | 1990-2014 | Iowa, USA | English | Retrospective chart review | To review patients who have undergone ASO at a single institution, with a focus on patients requiring multiple reinterventions. | ASO for TGA | 29/103 | Supravalvar pulmonary stenosis after the arterial switch operation for dextro-transposition of the great arteries is common and more than one reintervention are required in this subset of patients. |
| Jonas 2017 (216) | 1977-2016 | Vilnius Lithuania | English | Retrospective chart review | We report our 39-year experience in ASO’s and follow-up results | ASO for TGA | 73/115 | It is possible to perform arterial switch operations safely and with good outcome in a low-volume center. |
| Tabib 2017 (217) | 2010-2013 | Tehran, Iran | English | Retrospective chart review | To present an analytical overview of the results of ASO | ASO for TGA | 100/100 | This study showed a relatively high mortality rate in the patients undergoing ASO, in comparison to similar evidence, while the morbidity rate in the surviving patients was acceptable. |
| Talwar 2017 (218) | 2005-2016 | New Delhi India | English | Retrospective chart review | To evaluate out the early results ASO in such children more than one month of age | ASO for TGA | 446/470 | Late presentation of TGA is not uncommon. However acceptable results can be obtained in these patients |
| Martins 2017 (219) | 2017 | Sao Paula, Brazil | English | Retrospective chart review | To describe clinical and functional characteristics of patients undergoing Jatene procedure during long-term outpatient follow-up | ASO for TGA | 113/113 | In the long-term follow-up after Jatene procedure for anatomical correction of TGA, the incidence of neopulmonary stenosis was lower in those patients who underwent LeCompte maneuver. |
| Vida 2017 (220) | 1987-2016 | Padua Italy | English | Retrospective chart review | To evaluate our long-term results with ASO for dextro-TGA during the last 30 years | ASO for TGA | 260/260 | The ASO for dextro TGA can performed with low early  and late mortality. |
| Mekkawy 2017 (221) | 2012-2015 | Assiut, Egypt | English | Retrospective chart review | To evaluate the preoperative demographic, morphological and operative variables that affect the early outcome of the arterial switch procedure | ASO for TGA | 85 | ASO can be performed without excess mortality. |
| Gerelli 2017 (222) | 1987-2010 | Paris, France | English | Retrospective chart review | To evaluate the mortality and the coronary artery stenosis risk at early and long term in neonatal ASO for TGA and single CA. | Other | 73/73 | a single coronary artery is not any more a risk factor for early and late mortality after ASO for TGA. |
| Antsygin 2017 (223) | 2010-2016 | St Petersburg, Russia | English | Retrospective chart review | To evaluate our long-term results with ASO for dextro-TGA | ASO for TGA | 126/126 | ASO can be performed with a low risk of early mortality and satisfactory long-term outcomes. |
| Trezzi 2017 (224) | 2000-2015 | Rome, Italy | English | Retrospective chart review | To determine differences in baseline characteristics and clinical outcomes in a consecutive series of patients undergoing arterial switch operation (ASO), | ASO for TGA | 283/283 | In the recent era, unusual coronary patterns do not affect survival following ASO, |
| Michalak 2017 (225) | 1991-2015 | Lodz, Poland | English | Retrospective chart review | To compare the frequency of the most common and severe postoperative adverse events in patients with isolated TGA in reference to the complex cases | ASO for TGA | 715/715 | Primary late ASO has led to similar results compared with early ASO |
| Talwar 2017 (226) | 2002-2013 | Newcastle-upon-Tyne United Kingdom | English | Retrospective chart review | To review experiences with arterial switch operation in patients with transposition and a left-sided aorta. | ASO for TGA | 20/20 | With appropriate technical modifications, patients with concordant atrioventricular and discordant ventriculo-arterial connections with a left-sided aorta can undergo successful anatomical repair. |
| Sheriff 2017 (227) | 2011-2016 | Chennai-India | English | Retrospective chart review | To evaluate the midterm outcomes in patients with ASO for TGA | ASO for TGA | 78/78 | ASO remains the primary procedure of choice for simple TGA and acceptable choice for complex TG |
| Loss 2017 (228) | 2012-2016 | Vila Velha, Brazil | English | Retrospective chart review | To evaluate the outcomes in patients with ASO for TGA | ASO for TGA | 28/28 | The observed survival rate was 71.5%, with significant improvement after the first 2 years |
| Basile 2017 (229) | 2004-2016 | Milan Italy | English | Retrospective chart review | To investigate results of ASO performed after 21 days of life compared to “gold standard” repair performed within the first 21 days of life | ASO for TGA | 137/137 | Primary late ASO has led to similar results compared with early ASO |
| Michalak 2017 (230) | 1991-2015 | Lodz, Poland | English | Retrospective chart review | To assess the frequency of reoperations and catheter interventions in patients with TGA after the ASO and to identify the potential risk factors. | ASO for TGA | 715/715 | The frequency of reoperations and percutaneous interventions in patients with TGA after the ASO remains low. |
| Fricke 2017 (231) | 1983-2014 | Melbourne, Australia | English | Retrospective chart review | To determine outcomes of patients with weight less than 2.5 kg at ASO at a single institution. | Other | 31/31 | Early mortality for children weighing less than 2.5 kg undergoing the ASO remains high |
| Atalay 2017 (232) | 2007-2016 | Adana, Turkey | English | Retrospective chart review | To assess the early and midterm outcomes of patients operated by ASO for TGA | ASO for TGA | 34/34 | ASO remains the procedure of choice for the treatment of various forms of TGA with acceptable early and midterm outcome |
| Xiao 2018 (233) | 2007-2013 | Wuhan China | English | Retrospective chart review | This study was aimed to analysis the early and mid-term results of patients receiving ASO for TGA and Taussig-Bing anomaly | ASO for TGA | 119/119 | The outcomes of the ASO using our reconstruction and reimplantation techniques were excellent and the reoperation rate was very low in the early and mid-term follow-up. |
| Puska 2018 (234) | 2004-2014 | Helsinki, Finland | English | Retrospective chart review | To evaluate long-term outcomes of the arterial switch operation (ASO) for transposition of great arteries (TGA) in Finnish nationwide follow-up | ASO for TGA | 148 | Long-term survival after ASO is excellent. The most common reason for reintervention after ASO is pulmonary artery stenosis. |
| Martins 2018 (235) | 1997-2015 | Belo Horizonte | English | Retrospective chart review | To determine whether aortic angle predicts neo-aortic root dilatation and regurgitation following arterial switch operation | ASO for TGA | 157/180 | Acute aortic angles predict more extensive neo-aortic root dilatation and higher incidence of regurgitation. |
| Salna 2018 (236) | 2005-2015 | New York, USA | English | Retrospective chart review | To assess outcomes of the Arterial Switch Operation in ≤2.5-kg Neonates. | ASO for TGA | 217/217 | The ASO can be performed safely in 2.0- to 2.5-kg neonates and yields results comparable with higher weight infants. |
| Protopapas 2018 (237) | 1997-2017 | Athens, Greece | English | Retrospective chart review | To review the long-term results of the ASO in our experience, with emphasis on the need for re-intervention | ASO for TGA | 60/60 | Following the ASO for TGA, compared to atrial level repairs, complications requiring intervention have shifted from the inflow to the outflow of the ventricles |
| Michalak 2018 (238) | 1991-2016 | Lodz, Poland | English | Retrospective chart review | To present the frequency of coronary abnormalities in asymptomatic patients with transposition of the great arteries (TGA) after an ASO | ASO for TGA | 50/50 | The complex coronary setups with unfavorable, high risk features are common in patients with TGA after an arterial switch operation |
| Stoll 2018 (239) | 1985-2018 | Birmingham UK | English | Cohort study | To determine the pregnancy outcomes of women with transposition of the great arteries after an arterial switch operation, as well as the outcomes of their offspring. | ASO for TGA | 55/50 | Pregnancy is well tolerated after arterial switch operation |
| Kasmi 2018 (240) | 2017 | Paris, France | English | Observational/Cross sectional study | The present study investigated neurocognitive outcomes and the prevalence of psychiatric disorders in adults with d-TGA corrected by ASO. | Other | 67/67 | Adults who have undergone a neonatal ASO to correct d-TGA have an increased risk of cognitive deficits and psychiatric disorders. |
| Fricke 2018 (241) | 2018 | Melbourne, Australia | English | Observational/Cross sectional study | We sought to determine the quality of life after the arterial switch operation (ASO) using the Short Form 36 questionnaire in adult survivors. | ASO for TGA | 107/107 | Young adult survivors of the ASO have similar outcomes to age-matched controls in quality of life measured by Short Form 6-Dimension. |
| Joon Chul Jung 2018 (242) | 2003-2016 | Seoul, Republic of Korea | English | Retrospective chart review | To investigate the indications for reoperation and the results of reoperation to suggest methods to improve our management protocol for coronary artery stenosis after an ASO. |  | 86/86 | A standardized follow-up protocol including CT angiography or coronary angiography after the ASO is required to address coronary artery stenosis. |
| Nield 2018 (243) | 2009-2012 | Toronto, Canada | English | Cohort study | To determine whether specific coronary Doppler patterns intra-operatively predicted adverse early myocardial events in patients who underwent ASO for TGA. | ASO for TGA | 40/40 | Intra-operative evaluation of coronary artery flow patterns should be considered for patients undergoing the ASO. |
| Delmo 2010 (244) | 2010 | Boston USA | English | Retrospective chart review | To evaluated long-term outcome of aortic valve after arterial switch operation | ASO for TGA | 372/372 | The incidence of trivial or mild AR after ASO is considerable and progression over time is evident. |

^n= Number with Jatene operation for ventricular septal defect; m=total sample; ASO=Arterial Switch operation; TGA=Transposition of the Great Arteries; IVS = Intact ventricular septum; VSD = Ventricular septal defect; AI = Aortic Insufficiency; RCT=Randomised Controlled Trial; MRI = Magnetic Resonance Imager; USA= United States of America; UK= United Kingdom^

**References:**

1. Quaegebeur JM, Rohmer J, Ottenkamp J, Buis T, Kirklin JW, Blackstone EH, et al. The arterial switch operation: An eight-year experience. J Thorac Cardiovasc Surg. 1986 Sep;92(3 I):361–84.

2. Sidi D, Planche C, Kachaner J, Bruniaux J, Villain E, le Bidois J, et al. Anatomic correction of simple transposition of the great arteries in 50 neonates. Circulation. 1987 Feb;75(2):429–35.

3. G. W, T.J. H, E.P. W, G.F. S, S.D. C, S.P. S, et al. Midterm results after the arterial switch operation for transposition of the great arteries with intact ventricular septum: Clinical, hemodyanmic, echocardiographic, and electrophysiologic data. Circulation. 1988 Jun;77(6):1333–44.

4. Di Donato RM, Wernovsky G, Walsh EP, Colan SD, Lang P, Wessel DL, et al. Results of the arterial switch operation for transposition of the great arteries with ventricular septal defect. Surgical considerations and midterm follow-up data. Circulation. 1989 Dec;80(6):1689–705.

5. Backer CL, Ilbawi MN, Ohtake S, DeLeon SY, Muster AJ, Paul MH, et al. Transposition of the great arteries: A comparison of results of the Mustard procedure versus the arterial switch. Ann Thorac Surg. 1989 Jul;48(1):10–4.

6. M. Y, Y. H, Y. I, H. K, H. Y, T. Y, et al. Early and midterm results of the arterial switch operation for transposition of the great arteries in Japan. J Thorac Cardiovasc Surg. 1990;100(2):261–9.

7. M. P, M. F, Pozzi M, Fuchs M, Urban A. Anatomical correction of transposition of great vessels. Considerations on the first 50 patients operated on and analysis of the surgical choice. G Ital Cardiol. 1990 Dec;20(12):1125–9.

8. G. W, A. L, M. H, G. A. Early results with the anatomical correction of transposition of the great arteries. Thorac Cardiovasc Surg. 1991;39 Suppl 2:176–9.

9. Arterial switch operation for simple and complex TGA--indication criterias and limitations relevant to surgery. Thorac Cardiovasc Surg. 1991;39 Suppl 2:151–4.

10. D.C. B, G. W, L.A. R, J.E. MJ, A.R. C, D.M. F, et al. Cognitive development of children following early repair of transposition of the great arteries using deep hypothermic circulatory arrest. Pediatrics. 1991;87(5 I):701–7.

11. A. K, H.H. K, J. Q, J. O, B. K, E. G. The arterial switch-operation: early and midterm (6 years) results with particular reference to technical problems. Thorac Cardiovasc Surg. 1991;39 Suppl 2:160–5.

12. Mendoza JC, Wilkerson SA, Reese AH. Follow-up of patients who underwent arterial switch repair for transposition of the great arteries. Am J Dis Child. 1991 Jan;145(1):40–3.

13. Jatene FB, Bosisio IB, Jatene MB, Souza LC, Barbero-Marcial M, Jatene AD. Late results (50 to 182 months) of the Jatene operation. Eur J Cardiothorac Surg. 1992;6(11):575–8.

14. H.H. K, S. R, A. K, O. K, J. O, B. K, et al. Intermediate-term clinical and hemodynamic results of the neonatal arterial switch operation for complete transposition of the great arteries. Int J Cardiol. 1992 Jul;36(1):13–22.

15. J.W. K, E.H. B, C.I. T. Clinical outcomes after the arterial switch operation for transposition: Patient, support, procedural, and institutional risk factors. Circulation. 1992;86(5):1501–15.

16. Tashiro T, Todo K, Haruta Y, Tanaka K, Nagata M, Toyoda O, et al. Arterial switch operation for transposition of the great arteries without use of prosthetic material. Nihon Kyobu Geka Gakkai Zasshi. 1992 Jan;40(1):66–70.

17. Day RW, Laks H, Drinkwater DC. The influence of coronary anatomy on the arterial switch operation in neonates. J Thorac Cardiovasc Surg. 1992 Sep;104(3):706–12.

18. Binet JP, Planche C. [Development of a technique for the complete correction of transposition of great vessels]. C R Seances Soc Biol Fil. 1992;186(4):401–4.

19. Surgical management of transposition of the great arteries. Acta Chir Austriaca. 1993;25(2):80–2.

20. A.M. D, J.L. W, T.R. K, Davis AM, Wilkinson JL, Karl TR, et al. Transposition of the great arteries with intact ventricular septum: Arterial switch repair in patients 21 days of age or older. J Thorac Cardiovasc Surg. 1993 Jul;106(1):111–5.

21. Serraf A, Lacour-Gayet F, Bruniaux J, Touchot A, Losay J, Comas J, et al. Anatomic correction of transposition of the great arteries in neonates. J Am Coll Cardiol. 1993 Jul;22(1):193–200.

22. Ortega JL, Neira F, Garcia-Perla JL, Gutierrez JM. [Anesthesia and perioperative complications of correcting transposition of the great arteries using the Jatene technique]. Anest y Complic peroperatorias la transposicion las Gd Arter corregida Median la Tec Jatene. 1994;41(4):231–6.

23. S.N. W, G. W, S.D. C, J.A. P, C. B, S.M. M, et al. Myocardial perfusion, function and exercise tolerance after the arterial switch operation. J Am Coll Cardiol. 1994;23(2):424–33.

24. Kado H, Asoh T, Imoto Y, Shiokawa Y, Yamasaki M, Yasui H. [Reoperation for transposition of the great arteries: mid-term results and reoperation after arterial switch operation]. Rinsho Kyobu Geka. 1994 Jun;14(3):192–7.

25. Elkins RC, Knott-Craig CJ, Ahn JH, Murray CK, Overholt ED, Ward KE, et al. Ventricular function after the arterial switch operation for transposition of the great arteries. Ann Thorac Surg. 1994 Apr;57(4):826–31.

26. J.A. GH, J. CE, M. BS, J.A. LL, J. GM, J. S de S, et al. Anatomic correction of transposition of great vessels with entire interventricular septum. Initial results. Rev Esp Cardiol. 1995;48(5):333–40.

27. G. W, J.E. MJ, R.A. J, F.L. H, E.H. B, J.W. K, et al. Factors influencing early and late outcome of the arterial switch operation for transposition of the great arteries. J Thorac Cardiovasc Surg. 1995 Feb;109(2):289–302.

28. S.R. S, P.A. H, H.J. van de W, J.F. H, E.J. M. Late re-interventions following arterial switch operations in transposition of the great arteries. Incidence and surgical treatment of postoperative pulmonary stenosis. Eur J Cardiothorac Surg. 1995;9(1).

29. Bellinger DC, Jonas RA, Rappaport LA, Wypij D, Wernovsky G, Kuban KC, et al. Developmental and neurologic status of children after heart surgery with hypothermic circulatory arrest or low-flow cardiopulmonary bypass. N Engl J Med. 1995 Mar;332(9):549–55.

30. A. S, D. R, F. L-G, A. T, J. B, M. S-U, et al. Reoperation after the arterial switch operation for transposition of the great arteries. J Thorac Cardiovasc Surg. 1995 Oct;110(4 I):892–9.

31. G.B. L, A.C. C. Surgical repair of transposition of the great arteries in neonates with persistent pulmonary hypertension. Ann Thorac Surg. 1996;61(3):800–5.

32. Bonnet D, Bonhoeffer P, Piechaud JF, Aggoun Y, Sidi D, Planche C, et al. Long-term fate of the coronary arteries after the arterial switch operation in newborns with transposition of the great arteries. Heart. 1996 Sep;76(3):274–9.

33. P. B, D. B, J.-F. P, O. S, Y. A, E. V, et al. Coronary artery obstruction after the arterial switch operation for transposition of the great arteries in newborns. J Am Coll Cardiol. 1997;29(1):202–6.

34. S. C, F. L-G, A. S, J. B, A. T. The arterial switch era: Standards and evolving concepts in the surgery of TGA. Cor Eur - Eur J Card Interv. 1997;6(1):27–31.

35. Hovels-Gurich HH, Seghaye MC, Dabritz S, Messmer BJ, von Bernuth G. Cardiological and general health status in preschool- and school-age children after neonatal arterial switch operation. Eur J Cardiothorac Surg. 1997 Oct;12(4):593–601.

36. M. T, Y. I, Y. T, T. H, J. O. Improving surgical results for cardiovascular anomalies in neonates. Nippon Geka Gakkai zasshi. 1997;98(12):996–1000.

37. D. T, R. O, P. P, P. M, D. L, D. S, et al. Neonatal arterial switch operation: Coronary artery patterns and coronary events. Eur J Cardio-thoracic Surg. 1997 May;11(5):810–7.

38. Hovels-Gurich HH, Seghaye MC, Dabritz S, Messmer BJ, von Bernuth G. Cognitive and motor development in preschool and school-aged children after neonatal arterial switch operation. J Thorac Cardiovasc Surg. 1997 Oct;114(4):578–85.

39. Foran JP, Sullivan ID, Elliott MJ, de Leval MR. Primary arterial switch operation for transposition of the great arteries with intact ventricular septum in infants older than 21 days. J Am Coll Cardiol. 1998 Mar;31(4):883–9.

40. S. N, B.W. M, C. B, W.G. W, R.M. F, Nogi S, et al. Fate of the neopulmonary valve after the arterial switch operation in neonates. J Thorac Cardiovasc Surg. 1998 Mar;115(3):557–62.

41. Aseervatham R, Pohlner P. A clinical comparison of arterial and atrial repairs for transposition of the great arteries: early and midterm survival and functional results. Aust N Z J Surg. 1998 Mar;68(3):206–8.

42. Helvind MH, McCarthy JF, Imamura M, Prieto L, Sarris GE, Drummond-Webb JJ, et al. Ventriculo-arterial discordance: Switching the morphologically left ventricle into the systemic circulation after 3 months of age. Eur J Cardio-thoracic Surg. 1998 Aug;14(2):173–8.

43. Rappaport LA, Wypij D, Bellinger DC, Helmers SL, Holmes GL, Barnes PD, et al. Relation of seizures after cardiac surgery in early infancy to neurodevelopmental outcome. Boston Circulatory Arrest Study Group. Circulation. 1998 Mar;97(8):773–9.

44. Haas F, Wottke M, Poppert H, Meisner H, F. H, M. W, et al. Long-term survival and functional follow-up in patients after the arterial switch operation. Ann Thorac Surg. 1999 Nov;68(5):1692–7.

45. R. P, Q. Y, M. F, D. S, P. V, Pretre R, et al. Recent experience with the arterial switch operation in transposition of the great arteries. Schweiz Med Wochenschr. 1999 Oct;129(40):1443–9.

46. von Bernuth G. 25 years after the first arterial switch procedure: Mid-term results. Thorac Cardiovasc Surg. 2000 Aug;48(4):228–32.

47. J. A, T.L. G, A.L. C, P.J. R, Armishaw J, Gentles TL, et al. Transposition of the great arteries: operative outcome in the current era. N Z Med J. 2000 Nov;113(1121):456–9.

48. Daebritz SH, Nollert G, Sachweh JS, Engelhardt W, von Bernuth G, Messmer BJ, et al. Anatomical risk factors for mortality and cardiac morbidity after arterial switch operation. Ann Thorac Surg. 2000 Jun;69(6):1880–6.

49. P.A. H, W.Y. C, D.C. N, J.F. H, G.B.W.E. B. Arterial switch operation for transposition of the great arteries; favourable results at the Wilhelmina Children’s Hospital in Utrecht, the Netherlands, during the period 1977-2000. Ned Tijdschr Geneeskd. 2001;145(52):2534–8.

50. Pretre R, Tamisier D, Bonhoeffer P, Mauriat P, Pouard P, Sidi D, et al. Results of the arterial switch operation in neonates with transposed great arteries. Lancet (London, England). 2001 Jun;357(9271):1826–30.

51. J. W, E. B, N. S, H.C. B, A.M. B. Transposition of the great arteries associated with ventricular septal defect: surgical results and long-term outcome. Eur J Cardiothorac Surg. 2001;20(4):816–23.

52. Hovels-Gurich HH, Seghaye MC, Sigler M, Kotlarek F, Bartl A, Neuser J, et al. Neurodevelopmental outcome related to cerebral risk factors in children after neonatal arterial switch operation. Ann Thorac Surg. 2001 Mar;71(3):881–8.

53. Dunbar-Masterson C, Wypij D, Bellinger DC, Rappaport LA, Baker AL, Jonas RA, et al. General health status of children with D-transposition of the great arteries after the arterial switch operation. Circulation. 2001 Sep;104(12 Suppl 1):I138-42.

54. Y. T, S.-I. H, T. I. Fate of the aortic root after arterial switch operation. Eur J Cardio-thoracic Surg. 2001;20(1):82–8.

55. Kuroczynski W, Kampmann C, Choi YH, Hilker M, Wippermann F, David M, et al. [Treatment of supravalvular pulmonary stenosis after arterial switch operations (ASO)]. Z Kardiol. 2001 Jul;90(7):498–502.

56. Brown JW, Park HJ, Turrentine MW. Arterial switch operation: factors impacting survival in the current era. Ann Thorac Surg. 2001 Jun;71(6):1978–84.

57. Losay J, Touchot A, Serraf A, Litvinova A, Lambert V, Piot JD, et al. Late outcome after arterial switch operation for transposition of the great arteries. Circulation. 2001 Sep;104(12 Suppl 1):I121-6.

58. Sharma R, Bhan A, Choudhary SK, Kumar RP, Juneja R, Kothari SS, et al. Ten-year experience with the arterial switch operation. Indian Heart J. 2002;54(6):681–6.

59. Al Qethamy HO, Aizaz K, Aboelnazar SAR, Hijab S, Al Faraidi Y, H.O. AQ, et al. Two-stage arterial switch operation: Is late ever too late? Asian Cardiovasc Thorac Ann. 2002 Sep;10(3):235–9.

60. R. S, S.K. C, A. B, R.P. K, R. J, S.S. K, et al. Late outcome after arterial switch operation for complete transposition of great arteries with left ventricular outflow tract obstruction. Ann Thorac Surg. 2002;74(6):1986–91.

61. Gandhi SK, Pigula FA, Siewers RD, S.K. G, F.A. P. Successful late reintervention after the arterial switch procedure. Ann Thorac Surg. 2002 Jan;73(1):88–95.

62. H.H. H-G, M.-C. S, R. S, M. W, W. H, R. M, et al. Long-term neurodevelopmental outcomes in school-aged children after neonatal arterial switch operation. J Thorac Cardiovasc Surg. 2002;124(3):448–58.

63. Scheule AM, Zurakowski D, Blume ED, Stamm C, del Nido PJ, Mayer JEJ, et al. Arterial switch operation with a single coronary artery. J Thorac Cardiovasc Surg. 2002 Jun;123(6):1164–72.

64. H.H. H-G, K. K, M. W, R. M, B. H-D, B.J. M. Long term behavioural outcome after neonatal arterial switch operation for transposition of the great arteries. Arch Dis Child. 2002;87(6):506–10.

65. Prifti E, Crucean A, Bonacchi M, Bernabei M, Murzi B, Luisi SV, et al. Early and long term outcome of the arterial switch operation for transposition of the great arteries: predictors and functional evaluation. Eur J Cardiothorac Surg. 2002 Dec;22(6):864–73.

66. Hutter PA, Kreb DL, Mantel SF, Hitchcock JF, Meijboom EJ, Bennink GBWE. Twenty-five years’ experience with the arterial switch operation. J Thorac Cardiovasc Surg. 2002 Oct;124(4):790–7.

67. Tomizawa Y, Endo M, Nishida H, Koyanagi H. [Surgical repair of left ventricular aneurysm; long-term results]. Kyobu Geka. 2003;56(7):528–31.

68. A. L, J. L, A. T-K, A. S, E. B, J.-D. P, et al. Prevalence and diagnosis of coronary lesions after arterial switch. Arch Mal Coeur Vaiss. 2003;96(5):485–8.

69. Bellinger DC, Wypij D, duPlessis AJ, Rappaport LA, Jonas RA, Wernovsky G, et al. Neurodevelopmental status at eight years in children with dextro-transposition of the great arteries: The Boston Circulatory Arrest Trial. J Thorac Cardiovasc Surg. 2003 Nov;126(5):1385–96.

70. Williams WG, McCrindle BW, Ashburn DA, Jonas RA, Mavroudis C, Blackstone EH. Outcomes of 829 neonates with complete transposition of the great arteries 12-17 years after repair. Eur J Cardiothorac Surg. 2003 Jul;24(1):1–10.

71. Rehnstrom P, Gilljam T, Sudow G, Berggren H. Excellent survival and low complication rate in medium-term follow-up after arterial switch operation for complete transposition. Scand Cardiovasc J. 2003 May;37(2):104–6.

72. Hovels-Gurich HH, Seghaye M-C, Ma Q, Miskova M, Minkenberg R, Messmer BJ, et al. Long-term results of cardiac and general health status in children after neonatal arterial switch operation. Ann Thorac Surg. 2003 Mar;75(3):935–43.

73. Legendre A, Losay J, Touchot-Kone A, Serraf A, Belli E, Piot JD, et al. Coronary events after arterial switch operation for transposition of the great arteries. Circulation. 2003 Sep;108 Suppl:II186-90.

74. Formigari R, Toscano A, Giardini A, Gargiulo G, Di Donato R, Picchio FM, et al. Prevalence and predictors of neoaortic regurgitation after arterial switch operation for transposition of the great arteries. J Thorac Cardiovasc Surg. 2003 Dec;126(6):1753–9.

75. Wu Q, Shen X, Yang X, Li S, Yan J, Guo Y, et al. [Arterial switch operation in older infants with severe pulmonary hypertension]. Zhonghua Yi Xue Za Zhi. 2003 Mar;83(6):478–81.

76. JM B, Wypij D, DC B, LA R, LJ H, RA J, et al. Effect of prenatal diagnosis on outcomes in D-transposition of the great arteries. Pediatrics. 2004 Apr;113:e335-40.

77. K. M, J.A. MJA, A. S. Long term results of the arterial switch operation for transposition of the great arteries. Pol Prz Kardiol. 2004;6(2):195–202.

78. K.S. M, R. C, S. K, B. N. Arterial switch operation with in situ coronary reallocation for transposition of great arteries with single coronary artery. Eur J Cardio-thoracic Surg. 2004;25(2):246–9.

79. Xu Z, Ding W, Su Z, Chen L, Shi Z, Zhu D, et al. Arterial switch operation for transposition of the great arteries and double outlet of right ventricle with subpulmonary ventricular septum defect. Zhonghua Wai Ke Za Zhi. 2004 Apr;42(8):451–4.

80. Wu K-L, Lin M-T, Wu E-T, Lu FL, Chang C-I, Chiu I-S, et al. Arterial switch operation for transposition of the great arteries: Experience from 2000-2002 in Taiwan. Acta Paediatr Taiwanica. 2004;45(1):19–22.

81. D.J. D, A.E. A, W.K. V, E.D. M, C.D. FJ, I.L. K, et al. Current Expectations for Newborns Undergoing the Arterial Switch Operation. Ann Surg. 2004;239(5):588–98.

82. F. A, L. C, K. H, C. L, F. O, H. M. Anatomic repair of transposition of the great areries or arterial switch operation. Tunisie Medicale. 2004;82(1 SUPPL.):94–100.

83. Murthy KS, Coelho R, Kulkarni S, Ninan B, Cherian KM, K.S. M, et al. Arterial switch operation without coronary translocation: Mid-term results. Asian Cardiovasc Thorac Ann. 2004 Mar;12(1):38–40.

84. Lafuente M V, González F, Lara S, Salgado G, Suárez J, Laura JP, et al. Switch arterial: seguimiento a mediano plazo, 11 años de experiencia TT - Arterial switch: mid term follow up: eleven years of experience. Rev Argent Cardiol. 2005;73(2):107–11.

85. B. J, Z.-G. C. Surgical outcome of the arterial switch operation for transposition of the great arteries associated with ventricular septal defect. Fudan Univ J Med Sci. 2005;32(1):89–91.

86. Pocar M, Villa E, Degandt A, Mauriat P, Pouard P, Vouhe PR, et al. Long-term results after primary one-stage repair of transposition of the great arteries and aortic arch obstruction. J Am Coll Cardiol. 2005 Oct;46(7):1331–8.

87. D.H. F, C.M.T. R, R.S. S, A.R. J, I.M. R, D.B. R, et al. Intermediate-term outcomes of the arterial switch operation for transposition of great arteries in neonates: Alive but well? J Thorac Cardiovasc Surg. 2006 Oct;132(4):845.

88. Hwang HY, Kim W-H, Kwak JG, Lee JR, Kim YJ, Rho JR, et al. Mid-term follow-up of neoaortic regurgitation after the arterial switch operation for transposition of the great arteries. Eur J Cardiothorac Surg. 2006 Feb;29(2):162–7.

89. Park IS, Yoon SY, Min JY, Kim YH, Ko JK, Kim KS, et al. Metabolic alterations and neurodevelopmental outcome of infants with transposition of the great arteries. Pediatr Cardiol. 2006;27(5):569–76.

90. Marino BS, Wernovsky G, McElhinney DB, Jawad A, Kreb DL, Mantel SF, et al. Neo-aortic valvar function after the arterial switch. Cardiol Young. 2006 Oct;16(5):481–9.

91. C. P, A. H, E. L, R. M, E. S-D. Early and mid-term outcome of the arterial switch operation in 114 consecutive patients: A single centre experience. Clin Res Cardiol. 2007;96(10):723–9.

92. R. R, E. B, J. B, S. D, A. T, C. P. Surgery for Transposition of the Great Arteries in Neonates Weighing Less Than 2,000 Grams: A Consecutive Series of 25 Patients. Ann Thorac Surg. 2007;83(1):173–8.

93. Raisky O, Bergoend E, Agnoletti G, Ou P, Bonnet D, Sidi D, et al. Late coronary artery lesions after neonatal arterial switch operation: results of surgical coronary revascularization. Eur J Cardiothorac Surg. 2007 May;31(5):894–8.

94. Qamar ZA, Goldberg CS, Devaney EJ, Bove EL, Ohye RG. Current risk factors and outcomes for the arterial switch operation. Ann Thorac Surg. 2007 Sep;84(3):871–9.

95. M. A, T. F, H. N. Arterial switch operation: late results and a future perspective. Kyobu Geka. 2008;61(4):268–73.

96. T. N, H. K, K. H, A. S, M. K, O. A. Long-term results of arterial switch operation. Kyobu Geka. 2008;61(4):262–7.

97. E. A, O. R, D. B, D. S. Late reoperations after neonatal arterial switch operation for transposition of the great arteries. Eur J Cardio-thoracic Surg. 2008;34(1):32–6.

98. A. Y, N. Y, T. S, K. I, Y. I, G. M. Long-term outcomes and social independence level after arterial switch operation. Eur J Cardio-thoracic Surg. 2008;33(2):239–43.

99. Wong SH, Finucane K, Kerr AR, O’Donnell C, West T, Gentles TL, et al. Cardiac Outcome up to 15 Years After the Arterial Switch Operation. Hear Lung Circ. 2008 Feb;17(1):48–53.

100. S.M. K, A.B. S, Z.R. A-B. Neoaortic Bicuspid Valve in Arterial Switch Operation: Mid-Term Follow-Up. Ann Thorac Surg. 2008;85(1):179–84.

101. M.B. J, I.B. J, P.M. DO, R.A. M, L.C.B. DS, V. F, et al. Prevalence and surgical approach of supravalvular pulmonary stenosis after jatene operation for transposition of great arteries. Arq Bras Cardiol. 2008;91(1):18–24.

102. Bove T, De Meulder F, Vandenplas G, De Groote K, Panzer J, Suys B, et al. Midterm assessment of the reconstructed arteries after the arterial switch operation. Ann Thorac Surg. 2008 Mar;85(3):823–30.

103. W.B. DK, M. VO-G, A.D.J.T. H, R.T. VD, A.W. S, E.M.W.J. U, et al. Follow-up outcomes 10 years after arterial switch operation for transposition of the great arteries: Comparison of cardiological health status and health-related quality of life to those of the a normal reference population. Eur J Pediatr. 2008;167(9):995–1004.

104. T. N, Nishino T, Harada Y. Results of arterial switch operation for transposition of great arteries with regard to coronary pattern. Kyobu Geka. 2008 Apr;61(4):282–6.

105. A.J. R, T. W, N.A. A, I. D, M.A. B, F.W. M, et al. Moderate versus deep hypothermia for the arterial switch operation - experience with 100 consecutive patients. Eur J Cardio-thoracic Surg. 2008;33(4):619–25.

106. S. S, T. Y, K. K, Shiraishi S, Yagihara T, Kagisaki K, et al. Long-term result of arterial switch operation for corrected transposition of the great arteries or double inlet left ventricle. Kyobu Geka. 2008 Apr;61(4):311–5.

107. Lange R, Cleuziou J, Horer J, Holper K, Vogt M, Tassani-Prell P, et al. Risk factors for aortic insufficiency and aortic valve replacement after the arterial switch operation. Eur J Cardiothorac Surg. 2008 Oct;34(4):711–7.

108. Neufeld RE, Clark BG, Robertson CMT, Moddemann DM, Dinu IA, Joffe AR, et al. Five-year neurocognitive and health outcomes after the neonatal arterial switch operation. J Thorac Cardiovasc Surg. 2008 Dec;136(6):1413–21, 1421.e1-1421.e2.

109. J.J. M, K.W. M, J.A. M, K. M, T. M, M. M, et al. 18 years experience with Arterial Switch Operation (ASO) for Transposition of the Great Arteries (TGA). Interact Cardiovasc Thorac Surg. 2009;8(SUPPL. 1):S42.

110. K.D.H.M. V, N.A. B, S. L, D.R. K, M.E.B. R, Vandekerckhove KDHM, et al. Long-term follow-up of arterial switch operation with an emphasis on function and dimensions of left ventricle and aorta. Eur J Cardio-thoracic Surg. 2009 Apr;35(4):582–8.

111. J.G. C, M.G. E, P.J. B, M.A. F, P.C. F, M.E. M. Long-term outcomes of the neoaorta after arterial switch operation as palliation for Transposition of the Great Arteries: The Milwaukee experience. J Am Coll Cardiol. 2009;53(10):A358.

112. O. M, D. C, S. G, P. P, D. B, D. S, et al. Intramural coronary arteries and outcome of arterial switch operation for transposition of the great arteries. Interact Cardiovasc Thorac Surg. 2009;9(SUPPL. 2):S102.

113. E. A, R. F, C.P. N, G. O, F. P. Long-term coronary artery outcome after arterial switch operation for transposition of the great arteries. Interact Cardiovasc Thorac Surg. 2009;9(SUPPL. 2):S63.

114. Horer J, Schreiber C, Cleuziou J, Vogt M, Prodan Z, Busch R, et al. Improvement in long-term survival after hospital discharge but not in freedom from reoperation after the change from atrial to arterial switch for transposition of the great arteries. J Thorac Cardiovasc Surg. 2009 Feb;137(2):347–54.

115. L.Y. B, O.M. R, I.O. A, O.D. B, I.G. L. Arterial switch operation - Recent experience of 188 cases from a single institution. Cardiol Young. 2010;20(SUPPL. 1):180.

116. EM DW, Huebler M, Alexi-Meshkishvili V, Sill B, Berger F, Hetzer R. Fate of the aortic valve following the arterial switch operation. J Card Surg. 2010 Nov;25(6):730–6.

117. B.H.S. F, S.S. H, P.M.V.P. M, M.V.L. M, L.M. L, S.A.B. C, et al. Early outcome of the arterial switch (Jatene) operation in 173 consecutive patients. A single centre experience in a development country. Cardiol Young. 2010;20(SUPPL. 1):192.

118. W.-K. J, J.-J. P, T.-J. Y, Y.-H. K, J.-K. K, I.-S. P. Neo-aortic valve function after arterial switch operation for transposition of great arteries. Cardiol Young. 2010;20(SUPPL. 1):201.

119. S.G.G. R, M. K, A. A, N. O, B. M, I. S, et al. Mid-term follow-up of arterial switch operation for dtransposition with intact ventricular septum and left ventricular outflow tract obstruction. Interact Cardiovasc Thorac Surg. 2010;11(SUPPL. 2):S74.

120. H. G, D. B, M. O, A. T, E. L, M. W, et al. Long-term morbidity and quality of life after surgical repair of transposition of the great arteries: Atrial versus arterial switch operation. Interact Cardiovasc Thorac Surg. 2010;11(SUPPL. 2):S74–5.

121. S.R. I, M.S. K, H.K. N, R.M. A, M. E. Early outcome of primary arterial switch operation beyond 3 weeks of age. J Saudi Hear Assoc. 2010;22(2):91–2.

122. D. T, S.M. F, R.M. W, M. L, O. S, S.C. S, et al. Pregnancy outcomes in women with transposition of the great arteries and arterial switch operation. Am J Cardiol. 2010 Aug;106(3):417–20.

123. Park CS, Seo D-M, Park J-J, Kim YH, Park I-S, C.S. P, et al. The significance of pulmonary annulus size in the surgical management of transposition of the great arteries with ventricular septal defect and pulmonary stenosis. J Thorac Cardiovasc Surg. 2010 Jan;139(1):135–8.

124. Y.-L. L, S.-S. H, X.-D. S, S.-J. L, X. WW, J. Y, et al. Midterm results of arterial switch operation in older patients with severe pulmonary hypertension. Ann Thorac Surg. 2010 Sep;90(3):848–55.

125. B.S. C, B.S. K, G.B. K, E.J. B, C.I. N, J.Y. C, et al. Long-term outcomes after an arterial switch operation for simple complete transposition of the great arteries. Korean Circ J. 2010 Jan;40(1):23–30.

126. L. Y-L, H. S-S, S. X-D, L. S-J, W. XX, Y. J, et al. Safety and efficacy of arterial switch operation in previously inoperable patients. J Card Surg. 2010 Jul;25(4):400–5.

127. D. T, W.G. W, A. J, G.S. VA, B.W. M, M. G, et al. Cardiac Outcomes in Young Adult Survivors of the Arterial Switch Operation for Transposition of the Great Arteries. J Am Coll Cardiol. 2010 Jun;56(1):58–64.

128. Ismail SR, Kabbani MS, Najm HK, Abusuliman RM, Elbarbary M, S.R. I, et al. Early outcome for the primary arterial switch operation beyond the age of 3 weeks. Pediatr Cardiol. 2010 Jul;31(5):663–7.

129. El-Segaier M, Lundin A, Hochbergs P, Jogi P, Pesonen E. Late coronary complications after arterial switch operation and their treatment. Catheter Cardiovasc Interv. 2010 Dec;76(7):1027–32.

130. Metton O, Calvaruso D, Gaudin R, Mussa S, Raisky O, Bonnet D, et al. Intramural coronary arteries and outcome of neonatal arterial switch operation. Eur J Cardio-thoracic Surg. 2010 Jun;37(6):1246–53.

131. I.-S. C, S.-C. H, Y.-S. C, C.-I. C, M.-L. L, S.-J. C, et al. Restoring the spiral flow of nature in transposed great arteries. Eur J Cardio-thoracic Surg. 2010;37(6):1239–45.

132. K.W. M, J.A. M, K. M, M. K, A. S. Neoaortic valve function 10-18 years after arterial switch operation (ASO) for transposition of the great arteries (TGA). One centre experience. Cardiol Young. 2010;20(SUPPL. 1):30–1.

133. S. O, T. N, J. S. Twenty-eight years’ experience of arterial switch operation for transposition of the great arteries in a single institution. Interact Cardiovasc Thorac Surg. 2011;13(SUPPL. 2):S120.

134. M. K, A. K, V. H, M. N, J. M. Long-term neo-aortic growth in arterial switch operation versus Ross procedure. Is there a difference? Cardiol Young. 2011;21(SUPPL. 1):S46.

135. K.W. M, J.A. M, K. M, M. M, T. M, M. K. Neoaortic root in children with transposition of the great arteries (TGA) after arterial switch operation (ASO). Cardiol Young. 2011;21(SUPPL. 1):S3–4.

136. O. A, C. A, U. Y, K. T, O. S, R. T, et al. Follow-up of our patients with transposition of the great arteries and arterial switch operation; comparison of simple and complex transposition cases. Anadolu Kardiyol Derg. 2011 Dec;11(8):726–31.

137. Raja SG, Kostolny M, Oswal N, Afifi A, Mimic B, Sullivan ID, et al. Midterm follow-up of arterial switch operation for transposition of the great arteries with intact ventricular septum and left-ventricular outflow tract obstruction. Eur J Cardio-thoracic Surg. 2011 Oct;40(4):994–9.

138. J. C, N. A, S. M, M.-H. P, I. J. Impact of prenatal diagnosis of transposition of the great arteries on neurocognitive outcomes in preschool children. Congenit Heart Dis. 2011;6(5):530.

139. S. M, J. S, D.J. B, T.J. J. Does presence of ventricular septal defect influence survival after arterial switch operation for transposition of the great arteries? Cardiol Young. 2011;21(SUPPL. 1):S35–6.

140. Sterrett LE, Schamberger MS, Ebenroth ES, Siddiqui AR, Hurwitz RA. Myocardial perfusion and exercise capacity 12 years after arterial switch surgery for D-transposition of the great arteries. Pediatr Cardiol. 2011 Aug;32(6):785–91.

141. A. A, L. HL, A. S, F. C, B. C. A twenty year experience of arterial switch repair by direct anastomosis retaining a normal position of the pulmonary bifurcation. Cardiol Young. 2011;21(SUPPL. 1):S157.

142. H. G, M. O, A. T, E. L, M. W-B, A. H, et al. Long-term morbidity and quality of life after surgical repair of transposition of the great arteries: Atrial versus arterial switch operation. Interact Cardiovasc Thorac Surg. 2011;12(4):569–74.

143. Edwin F, Kinsley RH, Brink J, Martin G, Mamorare H, Colsen P. Late primary arterial switch for transposition of the great arteries with intact ventricular septum in an african population. World J Pediatr Congenit Heart Surg. 2011 Apr;2(2):237–42.

144. Jacobs JP, Jacobs ML, Mavroudis C, Chai PJ, Tchervenkov CI, Lacour-Gayet FG, et al. Transposition of the great arteries: lessons learned about patterns of practice and outcomes from the congenital heart surgery database of the society of thoracic surgeons. World J Pediatr Congenit Heart Surg. 2011 Jan;2(1):19–31.

145. Kim H, Sung SC, Kim S-H, Chang YH, Ahn HY, Lee HD. Arterial Switch Operation in Patients with Intramural Coronary Artery: Early and Mid-term Results. Korean J Thorac Cardiovasc Surg. 2011 Apr;44(2):115–22.

146. Leon-Wyss J, Lo Rito M, Barnoya J, Castaneda AR. Persistent institutional difficulties in surgery for transposition of the great arteries in guatemala: analysis with the aristotle basic and comprehensive scores. World J Pediatr Congenit Heart Surg. 2011 Jul;2(3):346–50.

147. Delmo Walter EM, Miera O, Nasseri B, Huebler M, Alexi-Meskishvili V, Berger F, et al. Onset of pulmonary stenosis after arterial switch operation for transposition of great arteries with intact ventricular septum. HSR Proc Intensive Care Cardiovasc Anesth. 2011;3(3):177–87.

148. Rudra HS, Mavroudis C, Backer CL, Kaushal S, Russell H, Stewart RD, et al. The arterial switch operation: 25-year experience with 258 patients. Ann Thorac Surg. 2011 Nov;92(5):1742–6.

149. Moll JJ, Michalak KW, Mludzik K, Moszura T, Kopala M, Moll M, et al. Long-term outcome of direct neopulmonary artery reconstruction during the arterial switch procedure. Ann Thorac Surg. 2012;93(1):177–84.

150. J. C, S. H, J. H, J. T. Anatomic mitral valve anomalies in D-transposition of the great arteries: Echo findings and impact on arterial switch repair technique and outcome. J Am Coll Cardiol. 2012;59(13 SUPPL. 1):E804.

151. K.W. M, J.A. M, K. M, M. M, T. M, P. D, et al. Long term outcome of arterial switch operation performed in neonates with the transposition of the great arteries. Cardiol Young. 2012;22(SUPPL. 1):S35.

152. A.-E. B, V. L, A. C, D. P, B. S, L. H, et al. The arterial switch operation for complex transposition of the great arteries: 20 years after. Circulation. 2012;126(21 SUPPL. 1).

153. S. L. Twelve years’ experience of arterial switch operation for transposition of the great arteries. S. H, editor. Cardiol. 2012;123(SUPPL. 1).

154. S. S, E. C, D. C, M. M, E. DC, D. I. Morbidity of the arterial switch operation. Ann Thorac Surg. 2012;93(6):1977–83.

155. J.-B. H, Y.-T. C, J. L. Quality of life after children undergo a radical arterial switch operation at an older age. Heart Surg Forum. 2012;15(2):E103–7.

156. T.A. F, Y. D, M. R, C. T, M. D, J.M. R, et al. Outcomes of the arterial switch operation for transposition of the great arteries: 25 years of experience. Ann Thorac Surg. 2012;94(1):139–45.

157. A.F. P, M. G, K.O. C, G.G. H, J. H, T. T, et al. Midterm results after arterial switch operation for transposition of the great arteries: A single centre experience. Artif Organs. 2012;36(5):A14.

158. Andropoulos DB, Easley RB, Brady K, McKenzie ED, Heinle JS, Dickerson HA, et al. Changing expectations for neurological outcomes after the neonatal arterial switch operation. Ann Thorac Surg. 2012 Oct;94(4):1250–6.

159. S. O, T. N, J. S, N. F, S. I. Twenty-eight years’ experience of arterial switch operation for transposition of the great arteries in a single institution. Eur J Cardio-thoracic Surg. 2012;42(4):674–9.

160. K. M. Arterial switch operation for transposition of the great arteries, unrestrictive ventricular septal defect, and pulmonary artieral hypertension. S. H, editor. Cardiol. 2012;123(SUPPL. 1).

161. Angeli E, Gerelli S, Beyler C, Lamerain M, Rochas B, Bonnet D, et al. Bicuspid pulmonary valve in transposition of the great arteries: Impact on outcome. Eur J Cardio-thoracic Surg. 2012 Feb;41(2):248–55.

162. Becker P, Delgado M, Frangini P, González R, Urcelay G, Clavería C, et al. Resultados inmediatos y alejados del switch arterial en pacientes condextrotransposición de grandes arterias: experiencia de 20 años TT - Early and late results of the switch operation for d-transposition of the great vessels. clinical experience in 108 . Rev chil cardiol. 2013;32(3):204–13.

163. T.P.E. R, A.E. van der B, J.A.A.E. C, M. W, W.A. H, A.J.J.C. B, et al. Long-term outcome and quality of life after arterial switch operation: A prospective study with a historical comparison. Congenit Heart Dis. 2013;8(3):203–10.

164. M. B, K. H, F. O, H. M. Long term outcome of arterial switch for transposition of great arteries in tunisian children. First experience in an emergent country. J Am Coll Cardiol. 2013;62(18 SUPPL. 2):C181.

165. T. VDB, R.L.F. VDP, B.J. B, S.L. VV, H.W. V, T.C. K, et al. Persistentneo-aortic growth during adulthood in patients after an arterial switch operation. Heart. 2014 Sep;100(17):1360–5.

166. A. N, L. G. Cardiac outcomes in adults post arterial switch operation for transposition of the great arteries. Hear Lung Circ. 2013;22(SUPPL. 1):S243.

167. M.M.P. D, J.M.P.J. B, R.R.J. VK, G.T.J. S, F.J. M. Coronary artery obstruction after the arterial switch operation: Is there really a long-term problem? J Am Coll Cardiol. 2013;61(10 SUPPL. 1):E538.

168. L. A, M. M, V. P, M. C, M. B, R. M, et al. 13-year experience with arterial switch operation in transposition of great arteries: Confirmations and new perspectives. G Ital Cardiol. 2013;14(10 SUPPL. 1):15S.

169. P. K, M. C, S.M. F, E.D. B, A.J. P, J.W. N, et al. Cardiovascular outcomes after the arterial switch operation for D-transposition of the great arteries. Circulation. 2013;127(3):331–9.

170. S. H, editor. Children with 230 cases of arterial switch operation surgical Methods and Result analysis. Cardiol. 2013;126(SUPPL. 1):146–7.

171. H.-G. L, W.-H. K, J.R. L, Lim H-G, Kim W-H, Lee JR, et al. Long-term results of the arterial switch operation for ventriculo-arterial discordance. Eur J Cardio-thoracic Surg. 2013 Feb;43(2):325–34.

172. D.M. K, V. L, A.-E. B, B. S, L. H, E. G, et al. Arterial switch for transposition with left outflow tract obstruction: Outcomes and risk analysis. Ann Thorac Surg. 2013;95(6):2097–103.

173. K.E. W, D.A. H, J.M. Q, E. B. Long term results of patients with d-transposition of the great arteries and native bicuspid pulmonary valves after the arterial switch operation. Circulation. 2013;128(22 SUPPL. 1).

174. L.D. S, I. S. Pulmonary artery reintervention following the arterial switch operation for transposition of the great arteries: A single centre experience. Cardiol Young. 2013;23(SUPPL. 1):S89–90.

175. Michalak KW, Moll JJA, Moll M, Dryzek P, Moszura T, Kopala M, et al. The neoaortic root in children with transposition of the great arteries after an arterial switch operation. Eur J Cardio-thoracic Surg. 2013 Jun;43(6):1101–8.

176. C.-H. L, J.-W. S, Z.-Q. L, X.-M. F, Y. C, Y. H, et al. Comparative analysis of early and middle outcomes of the arterial switch operation in children with complete transposition of the great arteries with ventricular septal defect and severe pulmonary artery hypertension. Chin Med J (Engl). 2013;126(11):2074–8.

177. H. A, S. S, A.A. A, S. C, M. B, G. A. Evaluation of cardiac and valvular function afer arterial switch operation: A midterm follow-up. Int Cardiovasc Res J. 2013;7(3).

178. M.T. C, Y. C, N.S. G, P.M. S, K. T, M.E. M, et al. Transposition of the Great Arteries-Outcomes and Time Interval of Early Neonatal Repair. World J Pediatr Congenit Hear Surg. 2014;5(2):241–7.

179. K.W. M, J.A. M, K. S-B, M. M, K. M, T. M, et al. Supravalvular pulmonary stenosis (SVPS) in patients with transposition of the great arteries (TGA) after arterial switch operation (ASO). Cardiol Young. 2014;24(Supplement 1):S112.

180. Bisoi AK, Ahmed T, Malankar DP, Chauhan S, Das S, Sharma P, et al. Midterm Outcome of Primary Arterial Switch Operation Beyond Six Weeks of Life in Children With Transposition of Great Arteries and Intact Ventricular Septum. World J Pediatr Congenit Hear Surg. 2014 Apr;5(2):219–25.

181. M.M.P. D, K.J. S-N, J.M.P.J. B, W.H.S. VW, N.J.G. J, P.H. S, et al. Coronary anatomy and lumen area in patients after the arterial switch operation: A long term follow-up study. Cardiol Young. 2014;24(Supplement 1):S25–6.

182. N. G, C. M, A. R, A. L, F. S, J.L. Z, et al. Long-term monitoring by MRI of coronary arteries after arterial switch operation in D transposition of the great arteries. Cardiol Young. 2014;24(Supplement 1):S101.

183. Parezanovic V, Mrdjen M, Illic S, Vulicevic I, Djukic M, Jovanovic I, et al. Mid-term results after complete surgical correction of transposition of the great arteries. Srp Arh Celok Lek. 2014;142(5–6):306–13.

184. A.K.M. H, A. H, T. K, B.J. M, R. S, R. M, et al. Neurologic and psycho-intellectual outcome related to structural brain imaging in adolescents and young adults after neonatal arterial switch operation for transposition of the great arteries. J Thorac Cardiovasc Surg. 2014 Nov;148(5):2190–9.

185. S. B, R. B, B. C, N. M, E. R, A. C, et al. Angiographic long-term follow-up after arterial switch operation: Experience of our institution. Cardiol Young. 2014;24(Supplement 1):S134–5.

186. De Praetere H, Vandesande J, Rega F, Daenen W, Marc G, Eyskens B, et al. 20 years of arterial switch operation for simple TGA. Acta Chir Belg. 2014;114(2):92–8.

187. Rodriguez Puras MJ, Cabeza-Letran L, Romero-Vazquianez M, Santos de Soto J, Hosseinpour R, Gil Fournier M, et al. Mid-term morbidity and mortality of patients after arterial switch operation in infancy for transposition of the great arteries. Rev Esp Cardiol (Engl Ed). 2014 Mar;67(3):181–8.

188. K.W. M, J.A. M, K. S-B, M. M, T. M, P. D, et al. Reinterventions and reoperations in patients with transposition of the great arteries after arterial switch operation (ASO). Cardiol Young. 2015;25(Supplement 1):S161–2.

189. B. N, J. N. Longitudinal echocardiographic follow up for right ventricular outflow obstruction after arterial switch. J Am Soc Echocardiogr. 2015;28(6):B110.

190. W.H.S. VW, M.M.P. D, P.H. S, N.J.G. J, F.J.M. M. Re-interventions after the arterial switch operation. Cardiol Young. 2015;25(Supplement 1):S6.

191. S. N, A. W, M. S. Transposition of great arteries, a 15 year experience of 74 patients in wales: Incorporation of outflow tract view in the antenatal scans leading to better antenatal detection and clinical outcomes. Arch Dis Child. 2015;100(SUPPL. 3):A69.

192. T. T, B. R, M. B, J. B. Unexpected late coronary artery abnormalities after arterial switch operation for transposition of the great arteries. J Am Coll Cardiol. 2015;65(10 SUPPL. 1):A553.

193. M. NM, A. PD, M.T. SD, Q. FM, S. PR, R. A, et al. Cardiac outcomes and prevalence of coronary stenosis in adult patients after arterial switch operation for transposition of the great arteries. Eur Heart J. 2015;36(SUPPL. 1):625.

194. O. Y, S. H, E. O, I.S. O, I.C. T, Y. E. Late primary arterial switch operation in patients with d-transposition and intact ventricular septum. Cardiol Young. 2015;25(Supplement 1):S157.

195. P.H. M, F.T.V. A, T.J.S. J, M.C. J, J. H, W.V.A. V, et al. Outcomes of Patients After Arterial Switch Operation: 18 Years of Experience in a Single Medium-Volume Center. Pediatr Cardiol. 2015;36(8):1657–61.

196. M. LR, M. F, R. H, T.J. J, N. K, P. C, et al. Long-term fate of the aortic valve after an arterial switch operation. J Thorac Cardiovasc Surg. 2015 Apr;149(4):1089–94.

197. K.W. M, J.A. M, K. S-B, M. M, T. M, P. D, et al. Coronary anomalies in children with transposition of the great arteries (TGA) and their impact on long term follow up after arterial switch operation. Cardiol Young. 2015;25(Supplement 1):S161.

198. C. J, K. H. Results of a small centre arterial switch programme - The Perth experience. Hear Lung Circ. 2015;24(Supplement 1):e67.

199. D.C. G, J.L. H, C.B. J. Does prenatal diagnosis of transposition of the great arteries improve survival? Cardiol Young. 2015;25(Supplement 1):S7–8.

200. Sun B-P, Fang S, Zhang Z-W, Chen F-X, Li J-H, Lin R, et al. Reconstruction of a new pulmonary artery in arterial switch operation. World J Pediatr. 2015 May;11(2):177–80.

201. Tsuda T, Bhat AM, Robinson BW, Baffa JM, Radtke W. Coronary artery problems late after arterial switch operation for transposition of the great arteries. Circ J. 2015;79(11):2372–9.

202. K.W. M, J.A. M, K. S-B, M. M, P. D, T. M, et al. Bicuspid neoaortic valve in patients with transposition of the great arteries after arterial switch operation: 25 years’ experience. Cardiol Young. 2016;26(Supplement 1):S37.

203. C.N. V, M.V. L, M. M, A. V, M.D. S, L. A, et al. Arterial switch operation: Long-term outcome. Rev Argent Cardiol. 2016;84(5):418–25.

204. A.-E. B, M. V, D. K, J.-G. D, M. L, A. C, et al. Long-term outcomes of the arterial switch operation for transposition of the great arteries and ventricular septal defect and/or aortic arch obstruction. Interact Cardiovasc Thorac Surg. 2016;23(2):240–6.

205. M.T. G-L, J.M. G-J, A.M. P-F, R. P-C-M, T. A-M. A 10-year experience with arterial switch operation in a single medium-volume institution: Improving outcomes over the years. Cardiol Young. 2016;26(Supplement 1):S164–5.

206. T. F, B. L, P. N, T. R, A. B, Y. D, et al. Outcomes of the arterial switch operation in patients with concomitant aortic arch obstruction as a single stage repair. Hear Lung Circ. 2017;26(Supplement 3):S396.

207. J. C, K. V, M. S, J. PVO, M. O, E. B, et al. Influence of aortic arch anomalies on long-term outcome of patients with TGA-VSD after the arterial switch operation. Cardiol Young. 2016;26(Supplement 1):S20.

208. Q. H, L.L. G, N. B, B. R, M. L, N. J, et al. Neonatal arterial switch operation: The sooner the better! Arch Cardiovasc Dis Suppl. 2016;8(1 SUPPL. 1):103–4.

209. T. F, P. N, A. B, T. R, Y. D, C. B. Outcomes of the arterial switch operation in adult survivors. Hear Lung Circ. 2016;25(8):e100.

210. Bokenkamp R, Aguilar E, van der Palen RLF, Sojak V, Bruggemans EF, Hruda J, et al. Reoperation for right ventricular outflow tract obstruction after arterial switch operation for transposition of the great arteries and aortic arch obstruction. Eur J Cardiothorac Surg. 2016 May;49(5):e91-6.

211. Shim M, Jun T-G, Yang J-H, Park PW, Kang IS, Huh J, et al. Current expectations of the arterial switch operation in a small volume center: a 20-year, single-center experience. J Cardiothorac Surg. 2016 Feb;11:34.

212. K. M, S. L, S. H, Z. H, K. Y, J. Y, et al. Neoaortic Valve Regurgitation After Arterial Switch: Ten Years Outcomes From A Single Center. Ann Thorac Surg. 2016 Aug;102(2):636–42.

213. E. E, D. S, O. Y, S. A, B. K, O.I. K, et al. Outcome of arterial switch operation for Taussig Bing anomaly versus transposition of the great arteries and ventricular septal defect. Cardiol Young. 2016;26(Supplement 1):S40–1.

214. Hicks MS, Sauve RS, Robertson CMT, Joffe AR, Alton G, Creighton D, et al. Early childhood language outcomes after arterial switch operation: a prospective cohort study. Springerplus. 2016;5(1):1681.

215. Nellis JR, Turek JW, Aldoss OT, Atkins DL, Ng BY. Intervention for Supravalvar Pulmonary Stenosis After the Arterial Switch Operation. Ann Thorac Surg. 2016 Jul;102(1):154–62.

216. K. J, V. J, R. S, V. L. 39 Years of experience of treating transposition of the great arteries in a single low volume center. Cardiol Young. 2017;27(4):S513–4.

217. A. T, R. B-T, A. J, M. M. Demographic, anatomic, and outcome survey in patients with arterial switch operation. Iran Hear J. 2017;18(3):42–51.

218. S. T, I. A, S. C. Early outcomes of the arterial switch operation in children of more than 1 month of age. Cardiol Young. 2017;27(4):S506.

219. C.L. M, N. J, D. M, M. W, E. F, R. S, et al. Long term outcomes of jatene procedure. Cardiol Young. 2017;27(4):S584.

220. V. V, L. Z, P. V, C. T, M. P, B. C, et al. Arterial switch operation for patients with transposition of the great arteries a single centre twenty eight year experience. Cardiol Young. 2017;27(4):S11–2.

221. A. M, A. G, O. E-H, J. P. Predictors of early outcome of arterial switch operation in patients with D-TGA. J Egypt Soc Cardio-Thoracic Surg. 2017;25(1):52–7.

222. Gerelli S, Pontailler M, Rochas B, Angeli E, Van Steenberghe M, Bonnet D, et al. Single coronary artery and neonatal arterial switch operation: Early and long-term outcomes. Eur J Cardio-thoracic Surg. 2017 Jul;52(1):90–5.

223. N. A, R. M, A. S, A. T, E. M. D transposition of the great arteries single center experience with arterial switch operation in the current era. Cardiol Young. 2017;27(4):S596.

224. Trezzi M, Polito A, Albano A, Albanese SB, Cetrano E, Carotti A. Intraoperative coronary revision but not coronary pattern is associated with mortality after arterial switch operation. Eur J Cardiothorac Surg. 2017 Jul;52(1):83–9.

225. K. M, M. M, K. S-B, M. K, T. M, P. D. Isolated vs complex transposition of the great arteries-is there still a difference in postoperative prognosis. Cardiol Young. 2017;27(4):S61–2.

226. Talwar S, Anderson RH, Ramakrishnan P, Bhoje A, Gupta S, Choudhary SK, et al. Arterial switch operation in patients with transposition and a left-sided aorta. Cardiol Young. 2017 Nov;27(9):1771–7.

227. Mid term outcomes of arterial switch operation a single centre experience. Cardiol Young. 2017;27(4):S540.

228. K.L. L, J. P, D.R. L, E.V. R, R.A. M, I. S, et al. Arterial switch surgery a 4 year experience of a small cardiac center in Brazil. Cardiol Young. 2017;27(4):S494–5.

229. D.P. B, M.L. R, A. M, J.F. S, A. S, G. G, et al. Arterial switch operation for transposition of great arteries with intact ventricular septum results of late primary repair. Cardiol Young. 2017;27(4):S62.

230. Michalak KW, Moll JA, Sobczak-Budlewska K, Moll M, Dryzek P, Moszura T, et al. Reoperations and catheter interventions in patients with transposition of the great arteries after the arterial switch operation. Eur J Cardiothorac Surg. 2017 Jan;51(1):34–42.

231. Fricke TA, Bulstra AE, Loyer BR, Weintraub RG, d’Udekem Y, Brizard CP, et al. Outcomes of the Arterial Switch Operation in Children Less Than 2.5 Kilograms. Ann Thorac Surg. 2017 Mar;103(3):840–4.

232. Atalay A, Gocen U. Early and Midterm Results of the Arterial Switch Operation: A 9-Year, Single-Center Experience. Heart Surg Forum. 2017 Feb;20(1):E015–8.

233. Y. X, P. Z, W. S, Xiao Y, Zhang P, Su W, et al. Early and mid-term follow-up of patients receiving arterial switch operation: A single-center experience. J Thorac Dis. 2018 Feb;10(2):732–9.

234. V. P, M. K, T. O, O. R, I. M. Long-term outcomes of the arterial switch operation in a population-based follow-up. Cardiol Young. 2018;28(Supplement 1):S155.

235. D. M, A. L, N. B, O. R, D. B. Aortic angle predicts neo-aortic root dilatation and regurgitation following arterial switch operation. Cardiol Young. 2018;28(Supplement 1):S59.

236. M. S, P.J. C, D. K, Y. N, G. K, J.M. Q, et al. Outcomes of the Arterial Switch Operation in <=2.5-kg Neonates. Semin Thorac Cardiovasc Surg. 2018 Apr;

237. E. P, P. Z, N. H, P. Z, C. A. Long term results after arterial switch operation (ASO) for transposition of the great arteries: Complications necessitating re-intervention. Cardiol Young. 2018;28(Supplement 1):S147.

238. K.W. M, M. M, K. S-B, J.A. M, K. S, P. D, et al. Can we predict the unfavorable coronary features in patients with transposition of the great arteries long-term after an arterial switch operation? Cardiol Young. 2018;28(Supplement 1):S13.

239. Stoll VM, Drury NE, Thorne S, Selman T, Clift P, Chong H, et al. Pregnancy Outcomes in Women With Transposition of the Great Arteries After an Arterial Switch Operation. JAMA Cardiol. 2018;

240. L. K, J. C, M. M, N. G, V. L, E. B, et al. Neurocognitive and Psychological Outcomes in Adults With Dextro-Transposition of the Great Arteries Corrected by the Arterial Switch Operation. Ann Thorac Surg. 2018 Mar;105(3):830–6.

241. Fricke TA, Loyer BR, Huang L, Griffiths S, Yaftian N, Dalziel KM, et al. Long-term quality of life in adult survivors after the arterial switch operation. Eur J Cardiothorac Surg. 2018 May;

242. Jung JC, Kwak JG, Kim ER, Bang JH, Min J, Lim JH, et al. Reoperation for coronary artery stenosis after arterial switch operation. Interact Cardiovasc Thorac Surg. 2018 Mar;

243. Nield LE, Dragulescu A, MacColl C, Manlhiot C, Brun H, McCrindle BW, et al. Coronary artery Doppler patterns are associated with clinical outcomes post-arterial switch operation for transposition of the great arteries. Eur Heart J Cardiovasc Imaging. 2018 Apr;19(4):461–8.

244. E.M. DW, B. S, M. H, V. A-M, F. B. Outcome of aortic valve after arterial switch operation. Thorac Cardiovasc Surg. 58(SUPPL. 1) -V137, DOI: 10.1055/s-0029-1246876.
