# Supplementary material for: Outcomes after corrective surgery for congenital dextro-transposition of the arteries using the arterial switch technique: a scoping systematic review
Source: Syst Rev. 2020 Oct 7;9:231. doi: 10.1186/s13643-020-01487-3 (PMC7542944; doi:10.1186/s13643-020-01487-3)
Supplement: Supplementary file 3 — Additional file 3. Appendix 3 Characteristics of excluded studies with reasons [file 13643_2020_1487_MOESM3_ESM.docx]

**Appendix 3: Characteristics of excluded studies with reasons**

| Article | Reason for exclusion |
| --- | --- |
| Bernuth et al 1990 (1) | No outcome of interest reported |
| Sano et al 2001 (2) | General overview article with no outcome of interest reported |
| Pasquali et al 2002 (3) | This was a meta-analysis of coronary artery pattern and outcome of ASO. No additional studies could be taken from this review |
| Rosti et al 2002 (4) | No outcome of interest reported. They report on growth of children post ASO |
| Kramer et al 2003 | No outcome of interest reported in this study |
| Mludzik et al 2003 (5) | No outcome of interest reported. Assesses risk factors for somatic growth and neurodevelopmental disturbance |
| Hovels-Gurich et al 2003 (6) | No outcome of interest reported. They report on exercise tolerance after ASO for TGA |
| Fernandes Fontes Pedra 2004 (7) | No outcome of interest reported |
| McGrath et al 2004 (8) | No outcome of interest reported. This article reports prediction of IQ from neurodevelopmental status in children with TGA |
| Qing-Yu Wu et al 2004 (9) | Wrong study population. it includes patients with the double outlet right ventricle |
| Hui et al 2005 (10) | No outcome of interest reported. They report on left ventricular function post ASO |
| Raja et al 2005 (11) | Review article. |
| Massin et al 2006 (12) | No outcome of interest reported. Measures physical activity threshold in children following ASO |
| Yamagishi 2008 (13) | No outcome of interest reported. Describes a new technique to prevent coronary complications during ASO |
| Giardini et al 2009 (14) | No outcome of interest reported. Lone outcome reported is exercise tolerance |
| Fredriksen et al 2009 (15) | No outcome of interest reported. Lone outcome reported is exercise tolerance using the treadmill test |
| Bellinger et al 2009 (16) | No outcome of interest reported. Report on behaviour of children 4 to 8 years post ASO for TGA as assessed by parents and teachers |
| Li et al 2009 (17) | No outcome of interest reported |
| Kaldararova et al 2010 (18) | No outcome of interest reported |
| Van Beek et al 2010 (19) | No outcome of interest reported. They report on exercise performance and activity level |
| Pasquali et al 2010 (20) | No outcome of interest reported. They compare obese children with ASO against normal controls |
| Weyand et al 2010 (21) | Comprised of a mixed group of patients with Rastelli Operation, Reparation a l'etage and ASO. Outcomes not reported specifically for ASO but for the whole group |
| Gorenflo et al 2011 (22) | No outcome of interest. Report on exercise tolerance post TGA |
| Xie et al 2011(23) | Insufficient information in the abstract to be exploited |
| Muller et al 2011 (24) | Inadequate amount of information in the abstract to assess reported quality of life measure |
| Squarcia et al 2011 (25) | This is a review article |
| Vouhe 2010 (26) | This is an editorial comment |
| Mueller et al 2011 (27) | No outcome of interest reported. Report on exercise performance |
| Akdeniz et al 2011 (28) | No outcome of interest reported |
| Rickers et al 2011 (29) | No ASO done in this study |
| Vargo et al 2011 (30) | This is a review article. |
| Horne et al 2012 (31) | No outcome of interest reported |
| Oner et al 2012 (32) | No outcome of interest reported. |
| Levy et al 2013 (33) | No outcome of interest reported |
| Junge et al 2013 (34) | No outcome of interest reported |
| Heinle et al 2013 (35) | Wrong study population. Included patients with single ventricle anatomy |
| Rickers et al 2014 (36) | No outcome of interest reported |
| Ahlstrom et al 2014 (37) | No outcome of interest reported |
| Schidlow et al 2014 (38) | No outcome of interest reported |
| Bokenkamp et al 2014 (39) | No outcome of interest reported |
| Doksoz et al 2014 (40) | No outcome of interest reported |
| Pizzi et al 2014 (41) | No outcome of interest reported |
| Marinov et al 2014 (42) | No outcome of interest reported |
| Pigula et al 2014 (43) | This is a commentary on a previous article with no study actually done |
| Karamlou 2014 (44) | No outcome of interest reported |
| Wiggins et al 2015 (45) | Wrong study population. patients who underwent RVOT reconstruction after ASO |
| Jenkinson et al 2015 (46) | This is meta-analysis |
| Kuebler et al 2015 (47) | No outcome of interest. Report on exercise tolerance post ASO |
| Cifra et al 2015 (48) | No outcome of interest. Report on systolic and diastolic BP post ASO as well as exercise tolerance |
| Grotenhuis et al 2015 (49) | No outcome of interest reported. Assesses systolic LV function post ASO |
| Abid et al 2015 (50) | No ASO done in this study. Reports outcomes of patients with TGA who were not operated |
| Hazekamp et al 2015 (51) | This is an opinion article. |
| Mostefa Kara et al 2015 (52) | Wrong study population, with a focus on patients with coronary lesions post ASO for TGA |
| Chawki et al 2015 (53) | Not a study but a review of surgical techniques for ASO hence wrong study design |
| Nicola Uricchio 2015 (54) | No ASO done in this study |
| Ruhaiyem et al 2016 (55) | No outcome of interest. Report on ventilation time |
| Maeda et al 2016 (56) | Wrong patient population as these patients had initially undergone a Senning/Mustard procedure |
| Michalak et al 2016 (57) | No outcome of interest. Report on coronary artery morphology before surgery |
| Samos et al 2016 (58) | No outcome of interest. Reports on exercise tolerance |
| Klaver et al 2016 (59) | No ASO done in this study |
| Cassidy et al 2016 (60) | No outcome of interest. Report on executive function organization |
| Shepard et al 2016 (61) | No outcome of interest reported |
| Andrade et al 2017 (62) | Not enough information in the abstract for abstraction |
| Heusch et al 2017 (63) | Wrong study population. Includes all cases with congenital heart disease |
| Kiener et al 2017 (64) | No outcome of interest reported. They only reported on transplant free survival post ASO for TGA |
| Manso et al 2017 (65) | No outcome of interest reported. They report on exercise tolerance post TGA |
| van Wijk et al 2017 (66) | This is a systematic review. |
| Lwin et al 2017 (67) | No outcome of interest reported. They report the Comprehensive Aristotle Complexity Scores |
| Tsuda et al 2017 (68) | No outcome of interest reported |
| Fricke et al 2017 (69) | Inadequate study population patients who underwent arterial switch operation (ASO) with concomitant aortic arch obstruction |
| Schidlow et al 2017 (70) | No outcome of interest reported |
| Kasmi et al 2017 (71) | Systematic review. |
| Lipczynska et al 2017 (72) | Wrong procedure. Studies women who received atrial switch operation |
| Willemijn et al 2017 (73) | Wrong study population, focuses on children with pulmonary arterial hypertension post ASO for TGA |
| Tabitha et al 2018 (74) | This is a review article. |
| Kiener et al 2018 (75) | No outcome of interest reported. Report on transplant free survival |
| Cleuziou et al 2018 (76) | No outcome of interest. Report on right ventricular outflow obstruction |
| Shiraishi et al 2018 (77) | No outcome of interest reported. Report on pulse pressure in the ascending aorta |
| Van Wijk et al 2018 (78) | This is a systematic review of left ventricular function and exercise capacity after ASO |
| Yildiz et al 2018 (79) | No outcome of interest reported |
| Hacker et al 2018 (80) | No outcome of interest reported. Reported on functional outcomes post ASO |
| Grotenhuis et al 2018 (81) | No outcome of interest reported. They report on left ventricular remodeling |
| Fricke et al 2018 (82) | Broadly discusses ASO operative approaches and potential outcomes |
| Cleuziou et al 2010 (83) | They report on re-intervention for right ventricular outflow tract obstruction, and no other outcome |
| Pedra et al 2004 (84) | No outcome of interest reported. They evaluated left ventricular function after Jatene operation |
| Torres et al 2014 (85) | Inadequate study population d-TGA, status post ASO, who underwent heart catheterization for pulmonary artery obstruction |
| Turon-Vinas et al 2014 (86) | No outcome of interest reported |
| Castro-Monsalve 2017 (87) | Wrong study population. It included patients undergoing left ventricular retraining post ASO for TGA |
| Genoni et al 1996 (88) | No ASO done in this study |
| Okuda 1985 (89) | No outcome of interest reported |
| Mee 1991 (90) | No ASO done in this study |
| Hazekamp et al 1991 (91) | No outcome of interest reported |
| Bertschmann et al 1991 (92) | No outcome of interest reported |
| Imai et al 1994 (93) | No ASO done in this study |
| Kaku, H et al 1997 (94) | No ASO done in this study |
| Bellinger 1997 (95) | No outcome of interest reported |
| Crepaz et al 1997 (96) | No outcome of interest reported |
| Masuda et al 1998 (97) | No ASO done in this study |
| Massin et at 1998 (98) | No outcome of interest reported |
| Blume et al 1998 (99) | No ASO done in this study |
| Eiriksson et al 1998 (100) | No outcome of interest reported |
| Massin 1999 (101) | No ASO done in this study |
| Derrick Cullen 2000 (102) | This is a general overview on transposition of the great arteries |
| Atik 2000 (103) | No ASO done in this study |
| Reybrouck et al 2001 (104) | No outcome of interest reported |

^IQ= Intelligence quotient; ASO=Arterial switch operation; TGA=Transposition of the Great Arteries; RVOT= Right ventricular outflow tract; BP= Blood pressure^

References

1. G. VB, M. D. The limits of early mobilization after cardiac surgery from the viewpoint of the pediatric cardiologist. Z Kardiol [Internet]. 1990;79(SUPPL. 4):145–52. Available from: http://ovidsp.ovid.com/ovidweb.cgi?T=JS&PAGE=reference&D=emed4&NEWS=N&AN=21033698

2. S. S, M. K. Surgical treatment of transposition of the great arteries: the arterial switch operation. Nippon Geka Gakkai zasshi [Internet]. 2001;102(8):584–9. Available from: http://ovidsp.ovid.com/ovidweb.cgi?T=JS&PAGE=reference&D=emed7&NEWS=N&AN=33519642

3. Pasquali SK, Hasselblad V, Li JS, Kong DF, Sanders SP, S.K. P, et al. Coronary artery pattern and outcome of arterial switch operation for transposition of the great arteries: A meta-analysis. Circulation [Internet]. 2002 Nov;106(20):2575–80. Available from: http://ovidsp.ovid.com/ovidweb.cgi?T=JS&PAGE=reference&D=emed7&NEWS=N&AN=35340694

4. Rosti L, Frigiola A, Bini RM, Giamberti A, Pome G, Chessa M, et al. Growth after neonatal arterial switch operation for D-transposition of the great arteries. Pediatr Cardiol. 2002;23(1):32–5.

5. H.-H. K, J. S, G. F, A. U, P. H, F. S. Long term follow-up of left ventricular performance and size of the great arteries before and after one- and two-stage arterial switch operation of simple transposition. Eur J Cardio-thoracic Surg [Internet]. 2003;24(6):898–905. Available from: http://ovidsp.ovid.com/ovidweb.cgi?T=JS&PAGE=reference&D=emed8&NEWS=N&AN=37476364

6. H.H. H-G, D. K, M.-C. S, M. M, B.J. M, Hovels-Gurich HH, et al. Results of exercise testing at a mean age of 10 years after neonatal arterial switch operation. Acta Paediatr Int J Paediatr [Internet]. 2003;92(2):190–6. Available from: http://ovidsp.ovid.com/ovidweb.cgi?T=JS&PAGE=reference&D=emed8&NEWS=N&AN=36390424

7. Pedra SRFF. Estudo da função ventricular esquerda da circulação coronária em pacientes com transposição das grandes artérias corrigida pela técnica de Jatene: resultados tardios TT - Lef ventricular function and coronary artery evaluation in the late follow-up after [Internet]. 2004. p. [147]-[147]. Available from: http://search.bvsalud.org/portal/resource/en/lil-397875

8. McGrath E, Wypij D, Rappaport LA, Newburger JW, Bellinger DC. Prediction of IQ and achievement at age 8 years from neurodevelopmental status at age 1 year in children with D-transposition of the great arteries. Pediatrics. 2004 Nov;114(5):e572-6.

9. Wu Q, Chu J. [Surgical treatment for double outlet of the right ventricle]. Zhonghua Wai Ke Za Zhi. 2004 Jan;42(2):65–7.

10. Hui L, Chau AKT, Leung MP, Chiu CSW, Cheung YF, L. H, et al. Assessment of left ventricular function long term after arterial switch operation for transposition of the great arteries by dobutamine stress echocardiography. Heart [Internet]. 2005 Jan;91(1):68–72. Available from: http://ovidsp.ovid.com/ovidweb.cgi?T=JS&PAGE=reference&D=emed9&NEWS=N&AN=39664997

11. S.G. R, A. S. Outcomes after arterial switch operation for simple transposition. Asian Cardiovasc Thorac Ann [Internet]. 2005;13(2):190–8. Available from: http://ovidsp.ovid.com/ovidweb.cgi?T=JS&PAGE=reference&D=emed9&NEWS=N&AN=40852200

12. M.M. M, H.H. H-G, P. G. Physical activity patterns of children after neonatal arterial switch operation. Ann Thorac Surg [Internet]. 2006;81(2):665–70. Available from: http://ovidsp.ovid.com/ovidweb.cgi?T=JS&PAGE=reference&D=emed9&NEWS=N&AN=43120575

13. Yamagishi M. [Technical modification and late results of arterial switch operation]. Kyobu Geka. 2008 Apr;61(4):297–302.

14. A. G, S. K, N. R, G. R, C.P. N, N. M, et al. Determinants of Exercise Capacity After Arterial Switch Operation for Transposition of the Great Arteries. Am J Cardiol [Internet]. 2009;104(7):1007–12. Available from: http://ovidsp.ovid.com/ovidweb.cgi?T=JS&PAGE=reference&D=emed11&NEWS=N&AN=355257699

15. Fredriksen PM, Pettersen E, Thaulow E, P.M. F, E. P. Declining aerobic capacity of patients with arterial and atrial switch procedures. Pediatr Cardiol [Internet]. 2009;30(2):166–71. Available from: http://ovidsp.ovid.com/ovidweb.cgi?T=JS&PAGE=reference&D=emed11&NEWS=N&AN=50245117

16. D.C. B, J.W. N, D. W, K.C.K. K, A.J. duPlesssis, Bellinger DC, et al. Behaviour at eight years in children with surgically corrected transposition: The Boston Circulatory Arrest Trial. Cardiol Young [Internet]. 2009 Feb;19(1):86–97. Available from: http://ovidsp.ovid.com/ovidweb.cgi?T=JS&PAGE=reference&D=emed11&NEWS=N&AN=354609616

17. Y.Q. L, S.J. L, P. L, X.B. P, Y.J. Z. Echocardiographic middle term follow-up of the arterial switch operation for transposition of the great arteries. S. H, editor. Cardiology [Internet]. 2009;114(SUPPL. 1):98. Available from: http://ovidsp.ovid.com/ovidweb.cgi?T=JS&PAGE=reference&D=emed11&NEWS=N&AN=70352996

18. M. K, I. S, P. T. Long-term fate of the neo-aorta after anatomic correction of d-transposition of the great arteries. Eur J Echocardiogr [Internet]. 2010;11(SUPPL. 2). Available from: http://ovidsp.ovid.com/ovidweb.cgi?T=JS&PAGE=reference&D=emed11&NEWS=N&AN=70316585

19. van Beek E, Binkhorst M, de Hoog M, de Groot P, van Dijk A, Schokking M, et al. Exercise performance and activity level in children with transposition of the great arteries treated by the arterial switch operation. Am J Cardiol. 2010 Feb;105(3):398–403.

20. Pasquali SK, Marino BS, Powell DJ, McBride MG, Paridon SM, Meyers KE, et al. Following the arterial switch operation, obese children have risk factors for early cardiovascular disease. Congenit Heart Dis. 2010;5(1):16–24.

21. Weyand K, Haun C, Blaschczok H, Goetz-Toussaint N, Photiadis J, Sinzobahamvya N, et al. Surgical treatment of transposition of great arteries with ventricular septal defect and left ventricular outflow tract obstruction: midterm results. World J Pediatr Congenit Heart Surg. 2010 Jul;1(2):163–9.

22. M. G, W. B, T. R. Exercise performance in complete and congenitally corrected transposition of the great arteries. Acta Cardiol [Internet]. 2011;66(1):128–9. Available from: https://secure.peeters-leuven.be/POJ/purchaseform.php?id=2064976sid=

23. F. X, H.-W. C. Primary arterial switch operation for D-transposition of the great arteries with intact ventricular septum in children older than 3 weeks. J Shanghai Jiaotong Univ (Medical Sci [Internet]. 2011;31(9):1250–3. Available from: http://xuebao.shsmu.edu.cn/EN/article/downloadArticleFile.do?attachType=PDF&id=9238

24. J. M, J. HH, J. HH, Muller J, Hess J, Horer J, et al. Persistent superior exercise performance and quality of life long-term after arterial switch operation compared to that after atrial redirection. Int J Cardiol [Internet]. 2013 Jun;166(2):381–4. Available from: http://ovidsp.ovid.com/ovidweb.cgi?T=JS&PAGE=reference&D=emed14&NEWS=N&AN=51722134

25. Squarcia U, Macchi C. Transposition of the great arteries. Curr Opin Pediatr. 2011 Oct;23(5):518–22.

26. Vouhe P. Editorial comment. Outcomes and re-interventions after one-stage repair of transposition of the great arteries and aortic arch obstruction. Eur J Cardio-thoracic Surg [Internet]. 2011;39(2):220–1. Available from: http://ovidsp.ovid.com/ovidweb.cgi?T=JS&PAGE=reference&D=emed12&NEWS=N&AN=51013626

27. J. M, J. H, J. H. Long-term follow-up of patients with transposition of the great arteries superior exercise performance and quality of life after arterial switch operation in comparison to atrial redirection. Eur Heart J [Internet]. 2011;32(SUPPL. 1):1033. Available from: http://ovidsp.ovid.com/ovidweb.cgi?T=JS&PAGE=reference&D=emed12&NEWS=N&AN=70536933

28. O. A, C. A, U. Y, K. T, O. S, R. T, et al. Follow-up of our patients with transposition of the great arteries and arterial switch operation; comparison of simple and complex transposition cases. Anadolu Kardiyol Derg [Internet]. 2011 Dec;11(8):726–31. Available from: http://www.anakarder.com/eng/sayilar/76/726-731.pdf

29. C. R, I. V, M. J-H, C. H, J. M, J. S. Premature aortic stiffening in patients with transposition of the great arteries after arterial switch operation. Circulation [Internet]. 2011;124(21 SUPPL. 1). Available from: http://ovidsp.ovid.com/ovidweb.cgi?T=JS&PAGE=reference&D=emed12&NEWS=N&AN=70618827

30. Vargo P, Mavroudis C, Stewart RD, Backer CL. Late complications following the arterial switch operation. World J Pediatr Congenit Heart Surg. 2011 Jan;2(1):37–42.

31. D. H, R. S, I. B, J. L, B. H. Supra-valvular pulmonary stenosis after arterial switch operation: Early post-operative echocardiographic predictors for re-intervention. Can J Cardiol [Internet]. 2012;28(5 SUPPL. 1):S105. Available from: http://ovidsp.ovid.com/ovidweb.cgi?T=JS&PAGE=reference&D=emed13&NEWS=N&AN=70943961

32. T. O, V. T, M.B. A, T. M, M.M. Y, B. G, et al. Short-to midterm assessment of the myocardial functions in patients with transposition of the great arteries after arterial switch. Heart Surg Forum [Internet]. 2012;15(SUPPL. 1):S84–5. Available from: http://www.metapress.com/content/378875533160m528/fulltext.pdf

33. M. L, R. C, D. C, P. V. Pulmonary arterial hypertension complicating arterial switch surgery for simple transposition of the great arteries-prevalence and outcomes. Arch Cardiovasc Dis Suppl [Internet]. 2013;5(1):98. Available from: http://ovidsp.ovid.com/ovidweb.cgi?T=JS&PAGE=reference&D=emed14&NEWS=N&AN=72045443

34. C. J, S. S, M. W-B, R. B, G. Z, S. G. Long-term follow-up of arterial and at rial switch operation for transposition of the great arteries. J Am Coll Cardiol [Internet]. 2013;61(10 SUPPL. 1):E539. Available from: http://ovidsp.ovid.com/ovidweb.cgi?T=JS&PAGE=reference&D=emed14&NEWS=N&AN=71019902

35. J.S. H, K.E. C, E.D. M, A. L, P.A. K. Outcomes after the palliative arterial switch operation in neonates with single-ventricle anatomy. Ann Thorac Surg [Internet]. 2013;95(1):212–9. Available from: http://ovidsp.ovid.com/ovidweb.cgi?T=JS&PAGE=reference&D=emed14&NEWS=N&AN=368039017

36. C. R, K. A, A. F, P. W, D. G, C. H, et al. Fluid dynamics and flow profiles in the great arteries in patients with transposition of the great arteries (TGA) after arterial switch operation with or without lecompte maneuver on long-term follow-up. Circulation [Internet]. 2014;130(SUPPL. 2). Available from: http://ovidsp.ovid.com/ovidweb.cgi?T=JS&PAGE=reference&D=emed15&NEWS=N&AN=71711117

37. L. A, S. J, T. M. Surgical age and morbidity after arterial switch of transposition of the great arteries. Cardiol Young [Internet]. 2014;24(Supplement 1):S151–2. Available from: http://ovidsp.ovid.com/ovidweb.cgi?T=JS&PAGE=reference&D=emed15&NEWS=N&AN=612966313

38. D.N. S, K.J. J, K. G, U.A. C, D.T.C. G, N.F. S, et al. Surgery and outcomes for transposition of the great arteries among developing world congenital heart surgery programs: A report from the international quality improvement collaborative. Circulation [Internet]. 2014;130(SUPPL. 2). Available from: http://ovidsp.ovid.com/ovidweb.cgi?T=JS&PAGE=reference&D=emed15&NEWS=N&AN=71711183

39. R. B, E. A, V. S, E. B, M. R, J. H, et al. Reoperation for right ventricular outflow tract obstruction after arterial switch operation for TGA and aortic arch obstruction. Cardiol Young [Internet]. 2014;24(Supplement 1):S31. Available from: http://ovidsp.ovid.com/ovidweb.cgi?T=JS&PAGE=reference&D=emed15&NEWS=N&AN=612966175

40. O. D, T. O, B. G, U. K, R. O, Y. Y, et al. Mid-term assessment of cardiac autonomic functions in children with transposition of the great arteries after arterial switch operation. Am J Cardiol [Internet]. 2014;113(7 SUPPL. 1):S22. Available from: http://ovidsp.ovid.com/ovidweb.cgi?T=JS&PAGE=reference&D=emed15&NEWS=N&AN=71390953

41. M.N. P, E. F, S. A-B, B. M, J. C-CC, G. C-B, et al. Long-term follow-up assessment after the arterial switch operation for correction of dextro-transposition of the great arteries by means of exercise myocardial perfusion-gated SPECT. Pediatr Cardiol [Internet]. 2014 Feb;35(2):197–207. Available from: http://ovidsp.ovid.com/ovidweb.cgi?T=JS&PAGE=reference&D=emed15&NEWS=N&AN=52676726

42. R. M, D. P, K. H, S. G, A. K. Global and regional myocardial function in patients after arterial switch operation-speckle tracking study. Cardiol Young [Internet]. 2014;24(Supplement 1):S128. Available from: http://ovidsp.ovid.com/ovidweb.cgi?T=JS&PAGE=reference&D=emed15&NEWS=N&AN=612966195

43. Pigula FA. Leaving the nest: facing adulthood after the arterial switch operation. Vol. 148, The Journal of thoracic and cardiovascular surgery. United States; 2014. p. 2199–200.

44. Karamlou T, Jacobs ML, Pasquali S, He X, Hill K, O’Brien S, et al. Surgeon and center volume influence on outcomes after arterial switch operation: analysis of the STS Congenital Heart Surgery Database. Ann Thorac Surg. 2014 Sep;98(3):904–11.

45. Wiggins LM, Kumar SR, Starnes VA, Wells WJ, L.M. W, S.R. K, et al. Arterioplasty for Right Ventricular Outflow Tract Obstruction After Arterial Switch Is a Durable Procedure. Ann Thorac Surg [Internet]. 2015 Jul;100(1):122–8. Available from: http://www.elsevier.com/locate/athoracsur

46. C. J, K. H. Twenty-five years of arterial switch operations-A metaanalysis. Hear Lung Circ [Internet]. 2015;24(Supplement 1):e9. Available from: http://ovidsp.ovid.com/ovidweb.cgi?T=JS&PAGE=reference&D=emed16&NEWS=N&AN=619390640

47. J.D. K, M.H. C. Longitudinal exercise performance in patients with d-loop transposition of the great arteries after arterial switch operation. J Am Coll Cardiol [Internet]. 2015;65(10 SUPPL. 1):A560. Available from: http://ovidsp.ovid.com/ovidweb.cgi?T=JS&PAGE=reference&D=emed16&NEWS=N&AN=71833617

48. B. C, C. S, M.K. F, L. M. Systolic and diastolic reserve in children after arterial switch operation. J Am Soc Echocardiogr [Internet]. 2015;28(6):B28. Available from: http://ovidsp.ovid.com/ovidweb.cgi?T=JS&PAGE=reference&D=emed16&NEWS=N&AN=71948651

49. H.B. G, L. M, B. C, E. R, C. M. Long-term survivors with transposition of the great arteries after arterial switch operation show no signs of adverse myocardial remodeling. Circulation [Internet]. 2015;132(SUPPL. 3). Available from: http://ovidsp.ovid.com/ovidweb.cgi?T=JS&PAGE=reference&D=emed16&NEWS=N&AN=72181229

50. D. A, A. L, A. BT, L. A, T. S, A. B, et al. Outcomes of transposition of great arteries in a country without possibility of surgical correction. Cardiol Young [Internet]. 2015;25(Supplement 1):S119. Available from: http://ovidsp.ovid.com/ovidweb.cgi?T=JS&PAGE=reference&D=emed16&NEWS=N&AN=612898291

51. Hazekamp MG. Long-term follow-up after the arterial switch operation: Not as perfect as we would have hoped? J Thorac Cardiovasc Surg [Internet]. 2015 Apr;149(4):968. Available from: http://www.elsevier.com/inca/publications/store/6/2/3/1/5/1/index.htt

52. M. MK, F. R, D. K, P. V, Y. B, O. R. Outcomes of coronary artery lesions after neonatal arterial switch operation. Cardiol Young [Internet]. 2015;25(Supplement 1):S115–6. Available from: http://ovidsp.ovid.com/ovidweb.cgi?T=JS&PAGE=reference&D=emed16&NEWS=N&AN=612898175

53. El-Zein C, Subramanian S, Ilbawi M. Evolution of the surgical approach to congenitally corrected transposition of the great arteries. Semin Thorac Cardiovasc Surg Pediatr Card Surg Annu. 2015;18(1):25–33.

54. Uricchio N, Ghiselli S, Marianeschi SM. [Transposition of the great arteries]. G Ital Cardiol (Rome). 2015 Feb;16(2):92–9.

55. M. R, N. A, R. S, R. AS. The outcome of arterial switch operation for d-TGA in relation to comprehensive aristotle score. Arch Dis Child [Internet]. 2016;101(Supplement 1):A82. Available from: http://ovidsp.ovid.com/ovidweb.cgi?T=JS&PAGE=reference&D=emed17&NEWS=N&AN=612211613

56. T. M, T. S, M. N, T. H. Long-Term Outcome of Arterial Switch Operation Conversion After Failed Senning/Mustard Procedure. Ann Thorac Surg [Internet]. 2016;102(5):1573–9. Available from: http://www.elsevier.com/locate/athoracsur

57. K.W. M, M. M, K. S-B, P. D, T. M, K. S, et al. Coronary artery anomalies and their impact on the outcome of the arterial switch operation for the transposition of the great arteries: 25 years’ experience. Cardiol Young [Internet]. 2016;26(Supplement 1):S162. Available from: http://ovidsp.ovid.com/ovidweb.cgi?T=JS&PAGE=reference&D=emed17&NEWS=N&AN=613004004

58. Samos F, Fuenmayor G, Hossri C, Elias P, Ponce L, Souza R, et al. Exercise Capacity Long-Term after Arterial Switch Operation for Transposition of the Great Arteries. Congenit Heart Dis [Internet]. 2016;11(2):155–9. Available from: http://onlinelibrary.wiley.com/journal/10.1111/(ISSN)1747-0803

59. P. K, W. K, K. W, Klaver P, Knirsch W, Wurmitzer K, et al. Children and Adolescents Show Altered Visual Working Memory Related Brain Activity More Than One Decade After Arterial Switch Operation for D-Transposition of the Great Arteries. Dev Neuropsychol [Internet]. 2016;41(4):261–7. Available from: http://ovidsp.ovid.com/ovidweb.cgi?T=JS&PAGE=reference&D=emed17&NEWS=N&AN=617574991

60. Cassidy AR, White MT, DeMaso DR, Newburger JW, Bellinger DC. Processing speed, executive function, and academic achievement in children with dextro-transposition of the great arteries: Testing a longitudinal developmental cascade model. Neuropsychology. 2016 Oct;30(7):874–85.

61. Shepard CW, Germanakis I, White MT, Powell AJ, Co-Vu J, Geva T. Cardiovascular Magnetic Resonance Findings Late After the Arterial Switch Operation. Circ Cardiovasc Imaging. 2016 Sep;9(9).

62. Andrade J, Lapa P, Tiago J, Marinho A, Ramalho R. 18-YEAR EXPERIENCE IN MANAGEMENT OF COMPLETE TRANSPOSITION OF THE GREAT ARTERIES IN NEWBORN: A SINGLE-CENTRE’S EXPERIENCE...XIII World Congress of Perinatal Medicine Belgrade, Serbia. October 26-29, 2017. J Perinat Med [Internet]. 2017 Oct 2;45:48. Available from: http://search.ebscohost.com/login.aspx?direct=true&db=cin20&AN=125873306&site=ehost-live

63. A. H, H.J. K, K.O. H, http://orcid.org/0000-0003-2805-1670 CGAO-HA. O. Health-related quality of life in paediatric patients with congenital heart defects: association with the type of heart defect and the surgical technique. Qual Life Res [Internet]. 2017;26(11):3111–7. Available from: http://www.wkap.nl/journalhome.htm/0962-9343

64. A. K, M. K, L. K. Long-term transplant-free survival for arterial switch versus atrial switch in treatment of transposition of the great arteries: A study from the pediatric cardiac care consortium. J Am Coll Cardiol [Internet]. 2017;69(11 Supplement 1):661. Available from: http://ovidsp.ovid.com/ovidweb.cgi?T=JS&PAGE=reference&D=emed18&NEWS=N&AN=617288847

65. P. M, M. F, T. S, L. Z, H. T, J. C. Anatomical and functional assessment of coronary arteries after arterial switch operation. Cardiol Young [Internet]. 2017;27(4):S155. Available from: http://ovidsp.ovid.com/ovidweb.cgi?T=JS&PAGE=reference&D=emexa&NEWS=N&AN=620076326

66. S.W.H. van W, F. van der S, H. ter H, P.H. S, P.A.F.M. D, F.J. M, et al. Sudden Death Due to Coronary Artery Lesions Long-term After the Arterial Switch Operation: A Systematic Review. Can J Cardiol [Internet]. 2017 Sep;33(9):1180–7. Available from: http://www.sciencedirect.com/science/journal/0828282X

67. N. L, K. E, S. J, H. H, N. A. Early functional health outcomes for infants and their families who have undergone arterial switch or Norwood procedures. Cardiol Young [Internet]. 2017;27(4):S145. Available from: http://ovidsp.ovid.com/ovidweb.cgi?T=JS&PAGE=reference&D=emexa&NEWS=N&AN=620077139

68. T. T, J. B, B. R, R. K, A. B. Acquired long-term cardiac rhythm and conduction abnormalities after arterial switch operation. J Am Coll Cardiol [Internet]. 2017;69(11 Supplement 1):601. Available from: http://ovidsp.ovid.com/ovidweb.cgi?T=JS&PAGE=reference&D=emed18&NEWS=N&AN=617289564

69. T. F, B. L, P. N, T. R, A. B, Y. D, et al. Outcomes of the arterial switch operation in patients with concomitant aortic arch obstruction as a single stage repair. Hear Lung Circ [Internet]. 2017;26(Supplement 3):S396. Available from: http://ovidsp.ovid.com/ovidweb.cgi?T=JS&PAGE=reference&D=emed18&NEWS=N&AN=619586940

70. D.N. S, K.J. J, K. G, U.A. C, D.T.C. G, R.K. K, et al. Transposition of the Great Arteries in the Developing World: Surgery and Outcomes. J Am Coll Cardiol [Internet]. 2017 Jan;69(1):43–51. Available from: http://www.elsevier.com/locate/jacc

71. Kasmi L, Bonnet D, Montreuil M, Kalfa D, Geronikola N, Bellinger DC, et al. Neuropsychological and Psychiatric Outcomes in Dextro-Transposition of the Great Arteries across the Lifespan: A State-of-the-Art Review. Front Pediatr. 2017;5:59.

72. M. L, P. S, O. T, L. T-P, B. P, A. K, et al. Pregnancy in women with complete transposition of the great arteries following the atrial switch procedure. A study from three of the largest Adult Congenital Heart Disease centers in Poland. J Matern Neonatal Med [Internet]. 2017;30(5):563–7. Available from: http://ovidsp.ovid.com/ovidweb.cgi?T=JS&PAGE=reference&D=emed18&NEWS=N&AN=610259633

73. Zijlstra WM, Elmasry O, Pepplinkhuizen S, Ivy DD, Bonnet D, Luijendijk P, et al. Pulmonary arterial hypertension in children after neonatal arterial switch operation. Heart. 2017 Aug;103(16):1244–9.

74. Moe TG, Bardo DME. Long-Term Outcomes of the Arterial Switch Operation for D-Transposition of the Great Arteries. Prog Cardiovasc Dis [Internet]. 2018; Available from: http://ovidsp.ovid.com/ovidweb.cgi?T=JS&PAGE=reference&D=medp&NEWS=N&AN=30227186

75. Kiener A, Kelleman M, McCracken C, Kochilas L, St Louis JD, Oster ME. Long-term Survival following Arterial vs. Atrial Switch in d-Transposition of the Great Arteries. Ann Thorac Surg [Internet]. 2018; Available from: http://ovidsp.ovid.com/ovidweb.cgi?T=JS&PAGE=reference&D=medp&NEWS=N&AN=30172857

76. J. C, K. V, J. P von O, M. O, D. T, M. B. Incidence and Risk Factors for Right Ventricular Outflow Tract Obstruction after the Arterial Switch Operation. Thorac Cardiovasc Surg [Internet]. 2018; Available from: http://www.thieme-connect.com/ejournals/toc/thoracic

77. M. S, A. T. Attenuation of pulse pressure amplification in elementary school-aged patients with complete transposition of great arteries after arterial switch operation. J Hypertens [Internet]. 2018;36(Supplement 1):e201. Available from: http://ovidsp.ovid.com/ovidweb.cgi?T=JS&PAGE=reference&D=emexa&NEWS=N&AN=623096499

78. S.W. VW, M.M. D, F.J. M, P.A. D, P.H. S, H.M. B, et al. Left ventricular function and exercise capacity after arterial switch operation for transposition of the great arteries: A systematic review and meta-Analysis. Cardiol Young [Internet]. 2018 Jul;28(7):895–902. Available from: http://www.journals.cambridge.org/action/displayJournal?jid=CTY

79. O. Y, E. O, S. O, C. T, Y. E, A. G. Arterial switch operation for transposition of the great arteries with intact ventricular septum in infants older than 21 days. Cardiol Young [Internet]. 2018;28(Supplement 1):S55. Available from: http://ovidsp.ovid.com/ovidweb.cgi?T=JS&PAGE=reference&D=emexa&NEWS=N&AN=622761594

80. A.-L. H, B. R, A. H, R. O, P. E. Functional outcomes in children with transposition of the great arteries after arterial switch operation. Cardiol Young [Internet]. 2018;28(Supplement 1):S27. Available from: http://ovidsp.ovid.com/ovidweb.cgi?T=JS&PAGE=reference&D=emexa&NEWS=N&AN=622761407

81. Grotenhuis HB, Cifra B, Mertens LL, Riessenkampff E, Manlhiot C, Seed M, et al. Left ventricular remodelling in long-term survivors after the arterial switch operation for transposition of the great arteries. Eur Heart J Cardiovasc Imaging. 2018 May;

82. Fricke TA, Konstantinov IE. Arterial Switch Operation: Operative Approach and Outcomes. Ann Thorac Surg. 2018 Jul;

83. J. C, C. S, J. H, Z. P, M. V. Right ventricular outflow tract obstruction after the arterial switch operation - Can we prevent it? Cardiol Young [Internet]. 2010;20(SUPPL. 1):185–6. Available from: http://ovidsp.ovid.com/ovidweb.cgi?T=JS&PAGE=reference&D=emed11&NEWS=N&AN=70235205

84. Pedra SRFF, Barretto RMB, Santana MVT, Pontes Jr SC, Pedra CAC, Gimenes VML, et al. Estudo da função ventricular esquerda de pacientes com transposição das grandes artérias corrigidas pela técnica de Jatene: resultados tardios TT - Left ventricular function assessment late after the arterial switch operation for transposition of the gre. Rev bras ecocardiogr [Internet]. 2004;17(4):47–56. Available from: http://search.bvsalud.org/portal/resource/en/lil-397762

85. A. T, B. A, M. T, M. C. Heart catheterization for residual pulmonary artery obstructive lesions in d-TGA following arterial switch operation. Catheter Cardiovasc Interv [Internet]. 2014;83(7):1193–4. Available from: http://ovidsp.ovid.com/ovidweb.cgi?T=JS&PAGE=reference&D=emed15&NEWS=N&AN=71498446

86. A. T-V, A. R-DV, J. M-GM-H, J. B-C, F.H. P-M, J. M-GM-H, et al. Characteristics and outcomes of transposition of great arteries in the neonatal period. Rev Esp Cardiol [Internet]. 2014 Feb;67(2):114–9. Available from: http://ovidsp.ovid.com/ovidweb.cgi?T=JS&PAGE=reference&D=emed15&NEWS=N&AN=52882199

87. J.M. C-M, C.X. F-R, D.H. M-B, J.C. G-N, A. B, S.E. M, et al. Left ventricle retrainning of D transposition of great arteries in the third world. The bucaramanga experience. Cardiol Young [Internet]. 2017;27(4):S65–6. Available from: http://ovidsp.ovid.com/ovidweb.cgi?T=JS&PAGE=reference&D=emexa&NEWS=N&AN=620075384

88. M. G, L. VS, M. W, U. A. 32 Years surgical correction of transposition of the great vessels. Swiss Surg [Internet]. 1996;2(SUPPL. 1):32–5. Available from: http://ovidsp.ovid.com/ovidweb.cgi?T=JS&PAGE=reference&D=emed6&NEWS=N&AN=26124969

89. H. O, M. N. Comparison of ventricular function after senning and Jatene procedures for complete transposition of the great arteries. Am J Cardiol [Internet]. 1985;55(5):530–4. Available from: http://ovidsp.ovid.com/ovidweb.cgi?T=JS&PAGE=reference&D=emed3&NEWS=N&AN=15118600

90. Mee RB. Results of the arterial switch procedure for complete transposition with an intact ventricular septum. Cardiol Young [Internet]. 1991;1(1):97–8. Available from: http://ovidsp.ovid.com/ovidweb.cgi?T=JS&PAGE=reference&D=prem&NEWS=N&AN=21114888

91. Hazekamp MG, Ottenkamp J, Quaegebeur JM, Hardjowijono R, Boot CA, Rohmer J, et al. Follow-up of arterial switch operation. Thorac Cardiovasc Surg. 1991 Dec;39 Suppl 2:166–9.

92. W. B. Arterial switch operation in great vessels transposition and double outlet right ventricle. Helv Chir Acta [Internet]. 1990;57(4):545–9. Available from: http://ovidsp.ovid.com/ovidweb.cgi?T=JS&PAGE=reference&D=emed4&NEWS=N&AN=21112002

93. Imai Y, Sawatari K, Hoshino S, Ishihara K, Nakazawa M, Momma K. Ventricular function after anatomic repair in patients with atrioventricular discordance. J Thorac Cardiovasc Surg. 1994 May;107(5):1272–83.

94. H. K, T. A, Y. S, K. F, Y. M, M. S, et al. Follow-up results of Jatene surgery of total transposition of great vessels. Nihon Kyobu Geka Gakkai Zasshi [Internet]. 1997;45(3):325–7. Available from: http://ovidsp.ovid.com/ovidweb.cgi?T=JS&PAGE=reference&D=emed6&NEWS=N&AN=127269831

95. D.C. B, L.A. R, D. W, G. W. Patterns of developmental dysfunction after surgery during infancy to correct transposition of the great arteries. J Dev Behav Pediatr [Internet]. 1997;18(2):75–83. Available from: http://ovidsp.ovid.com/ovidweb.cgi?T=JS&PAGE=reference&D=emed6&NEWS=N&AN=127255239

96. R. C, S. S, G. S, O. M, W. P, L. G, et al. Echocardiographic evaluation of systolic and diastolic left ventricular function following arterial switch operation in the neonatal period for transposition of the great arteries. Midterm results. G Ital Cardiol [Internet]. 1997 Mar;27(3):224–30. Available from: http://ovidsp.ovid.com/ovidweb.cgi?T=JS&PAGE=reference&D=emed6&NEWS=N&AN=127278103

97. Masuda M, Yasui H. [Reoperation after repair of complete transposition of the great arteries]. Nihon Geka Gakkai Zasshi. 1998 Feb;99(2):78–83.

98. M. M, H. H-G, S. D, B. M. Results of the Bruce treadmill test in children after arterial switch operation for simple transposition of the great arteries. Am J Cardiol [Internet]. 1998;81(1):56–60. Available from: http://ovidsp.ovid.com/ovidweb.cgi?T=JS&PAGE=reference&D=emed6&NEWS=N&AN=28046843

99. Blume ED, Wernovsky G. Long-term results of arterial switch repair of transposition of the great vessels. Semin Thorac Cardiovasc Surg Pediatr Card Surg Annu. 1998;1:129–38.

100. Eiriksson H, Helgason H. [Transposition of the great arteries in Iceland over a 26 year period from 1971 to 1996.]. Laeknabladid. 1998 Jul;84(7):533–40.

101. Massin MM. Midterm results of the neonatal arterial switch operation. A review. J Cardiovasc Surg (Torino) [Internet]. 1999 Aug;40(4):517–22. Available from: http://ovidsp.ovid.com/ovidweb.cgi?T=JS&PAGE=reference&D=emed6&NEWS=N&AN=29465447

102. Derrick, Cullen. Transposition of the Great Arteries. Curr Treat Options Cardiovasc Med [Internet]. 2000;2(6):499–506. Available from: http://ovidsp.ovid.com/ovidweb.cgi?T=JS&PAGE=reference&D=prem&NEWS=N&AN=11096553

103. Atik E. Transposiçäo das Grandes artérias. Avaliaçäo dos resultados e a conduta atual TT - Transposition of the great arteries. Evaluation of the results and current management. Arq Bras Cardiol [Internet]. 2000;75(2):91–6. Available from: http://www.scielo.br/scielo.php?script=sci_arttext&pid=S0066-782X2000000800001&lng=en&nrm=iso&tlng=en

104. Reybrouck T, Eyskens B, Mertens L, Defoor J, Daenen W, Gewillig M, et al. Cardiorespiratory exercise function after the arterial switch operation for transposition of the great arteries. Eur Heart J [Internet]. 2001 Jun;22(12):1052–9. Available from: http://ovidsp.ovid.com/ovidweb.cgi?T=JS&PAGE=reference&D=emed7&NEWS=N&AN=32743141
