# Supplementary material for: Outcomes after corrective surgery for congenital dextro-transposition of the arteries using the arterial switch technique: a scoping systematic review
Source: Syst Rev. 2020 Oct 7;9:231. doi: 10.1186/s13643-020-01487-3 (PMC7542944; doi:10.1186/s13643-020-01487-3)
Supplement: Supplementary file 4 — Additional file 4. Appendix 4 Summary of findings table: Arterial switch operation for transposition of the great arteries. [file 13643_2020_1487_MOESM4_ESM.docx]

**Appendix 4: Summary of findings table: Arterial switch operation for transposition of the great arteries**

| **ASO for TGA in children with TGA** | | | | | | | | | | |
| --- | --- | --- | --- | --- | --- | --- | --- | --- | --- | --- |
| **Certainty assessment** | | | | | | | **Summary of findings** | | | |
| **№ of participants (studies) Follow-up** | **Risk of bias** | **Inconsistency** | **Indirectness** | **Imprecision** | **Other considerations** | **Overall certainty of evidence** | **Study event rates (%)** | | **Impact** |  |
|  |  |  |  |  |  |  | **With [comparison]** | **With ASO for TGA** |  |  |
| **1. Short term survival (assessed with: Proportion of ASO patients surviving between 1 day up to 1 year)** | | | | | | | | | | |
| (151 observational studies) | not serious | not serious | not serious | not serious | Large effect ^a^ | ⨁⨁⨁◯ MODERATE | The pooled prevalence for short term survival was 92.0% (95% CI 91.0 – 93.0%, I^2^ =85.8%) | | | |
| **2. Medium term survival (assessed with: Proportion of ASO patients surviving between 1 to 20 years)** | | | | | | | | | | |
| (133 observational studies) | not serious | not serious | not serious | not serious | Large effect ^a^ | ⨁⨁⨁◯ MODERATE | The pooled prevalence of medium term survival was 90.0% (95% CI 89.0 – 91.0%, I^2^=84.3%) | | | |
| **3. Long term survival (assessed with: Proportion of ASO patients surviving 20 years and above)** | | | | | | | | | | |
| (4 observational studies) | not serious | serious ^b^ | not serious | serious ^c^ | Large effect ^d^ | ⨁◯◯◯ VERY LOW | The pooled prevalence of long term survival was 87.0% (95% CI 80.0 – 92.0%, I^2^=84.5%) | | | |
| **4. Short term freedom from re-operation (assessed with: Proportion of ASO patients free from cardiac re-operations up to age of 1 year)** | | | | | | | | | | |
| (43 observational studies) | not serious | not serious | not serious | not serious | Large effect ^a^ | ⨁⨁⨁◯ MODERATE | The pooled prevalence for the short term freedom from re-operation was 93.0% (95% CI 91.0-95.0%, I^2^=92.3%) | | | |
| **5. Medium term freedom from re-operation New outcome (assessed with: Proportion of ASO patients free from re-operations between 1 to 20 years)** | | | | | | | | | | |
| (110 observational studies) | not serious | not serious | not serious | not serious | Large effect ^a^ | ⨁⨁⨁◯ MODERATE | The pooled prevalence for medium term freedom from re-operation was 81.0% (95% CI 78.0 – 84.0%, I^2^=95.6%) | | | |
| **6. Long term freedom from re-operation (assessed with: Proportion of ASO patients free from re-operation after 20 years)** | | | | | | | | | | |
| (6 observational studies) | not serious | serious ^b^ | not serious | serious ^c^ | Large effect ^d^ | ⨁◯◯◯ VERY LOW | The pooled prevalence for long term freedom from re-operation was 78.0% (95% CI 69.0 – 86.0%, I^2^=95.5) | | | |
| **7. Short term occurrence of Aortic insufficiency (assessed with: Proportion of ASO patients developing aortic insufficiency with 1 year)** | | | | | | | | | | |
| (19 observational studies) | not serious | not serious | not serious | not serious | publication bias strongly suspected strong association ^e^ | ⨁⨁◯◯ LOW | The pooled prevalence of short term occurrence of aortic insufficiency was 4.0% (95% CI 2.0 – 7.0%, I^2^=96.2%) | | | |
| **8. Medium term occurrence of aortic insufficiency (assessed with: Proportion of ASO patients developing aortic insufficiency between 1 to 20 years)** | | | | | | | | | | |
| (65 observational studies) | not serious | not serious | not serious | not serious | Large effect ^a^ | ⨁⨁⨁◯ MODERATE | The pooled prevalence of medium term occurrence of aortic insufficiency was 22.0% (95% CI 17.0 – 26.0 %, I^2^=96.9) | | | |
| **9 Long term occurrence of aortic insufficiency (assessed with: Proportion of ASO patients developing aortic insufficiency after 20 years)** | | | | | | | | | | |
| (2 observational studies) | not serious | not serious | not serious | serious ^c^ | publication bias strongly suspected ^f^ | ⨁◯◯◯ VERY LOW | The overall prevalence of long term occurrence of aortic insufficiency was 29.0% (95% CI 25.0 - 33.0%, I^2^=0.0) | | | |
| **10 Short term occurrence of pulmonary stenosis (assessed with: Proportion of ASO patients developing pulmonary stenosis within 1 year)** | | | | | | | | | | |
| (9 observational studies) | not serious | not serious | not serious | not serious | Large effect | ⨁⨁⨁◯ MODERATE | The pooled prevalence of short term occurrence of pulmonary stenosis was 5.0% (95% CI 2.0 – 9.0 %, I^2^=85.5%) | | | |
| **11 Medium term occurrence of pulmonary stenosis (assessed with: Proportion of ASO patients developing pulmonary stenosis between 1 to 20 years)** | | | | | | | | | | |
| (54 observational studies) | not serious | not serious | not serious | not serious | Large effect | ⨁⨁⨁◯ MODERATE | The pooled prevalence for medium term occurrence of pulmonary stenosis is 12.0% (10.0 – 15.0%, I^2^=92.8%) | | | |
| **12 Long term occurrence of pulmonary stenosis (assessed with: Proportion of ASO patients developing pulmonary stenosis after 20 years)** | | | | | | | | | | |
| (1 observational study) | not serious | not serious | not serious | very serious ^g^ | publication bias strongly suspected strong association ^f^ | ⨁◯◯◯ VERY LOW | The overall prevalence of long term occurrence of pulmonary stenosis was 82.0% (95% CI 77.0-86.0, I^2^ N/A) | | | |
| **13 Short term occurrence of any coronary anomaly (assessed with: Proportion of ASO patients developing coronary anomaly within 1 years)** | | | | | | | | | | |
| (5 observational studies) | not serious | not serious | not serious | not serious ^c^ | publication bias strongly suspected strong association ^f^ | ⨁⨁◯◯ LOW | The pooled prevalence of short term occurrence of coronary anomaly is 1.0 % (95% CI 0.0 – 4.0 %, I^2^=59.7%) | | | |
| **14 Medium term occurrence of coronary anomaly (assessed with: Proportion of ASO patients developing any coronary anomaly within 1 to 20 years)** | | | | | | | | | | |
| (37 observational studies) | not serious | not serious | not serious | not serious | publication bias strongly suspected strong association ^e^ | ⨁⨁◯◯ LOW | The pooled prevalence for the medium term occurrence of any coronary anomaly was 8.0% (95% CI 5.0 – 11.0%, I^2^=93.3) | | | |
| **15. Long term occurrence of coronary anomaly (assessed with: Proportion of ASO patients developing any coronary anomaly after 20 years)** | | | | | | | | | | |
| (2 observational studies) | not serious | not serious | not serious | serious ^c^ | none | ⨁◯◯◯ VERY LOW | The pooled prevalence of long term occurrence of any coronary anomaly was 23.0 % (95% CI 16.0 – 31.0%, I^2^=N/A) | | | |
| **16. Neuropsychiatric outcome: Mental development index (assessed with: The Z score for mental development index across studies)** | | | | | | | | | | |
| (17 observational studies) | not serious | not serious | not serious | not serious | strong association | ⨁⨁⨁◯ MODERATE | The overall pooled weighted Z score for mental development index of ASO patients was -0.10 (95% CI -0.8 - 0.6, I^2^=0.0%) | | | |
| **17. Neuropsychiatric outcome: Mental development index (assessed with: the Z score for physical development index across studies)** | | | | | | | | | | |
| (17 observational studies) | not serious | not serious | not serious | not serious | strong association | ⨁⨁⨁◯ MODERATE | The overall pooled weighted Z score for physical development index of ASO patients was -0.3 (95% CI -1.9 – 1.2, I^2^=0.0%) | | | |
| **18. Quality of life outcomes (assessed with: across different studies with different scales)** | | | | | | | | | | |
| (7 observational studies) | not serious | serious ^h^ | not serious | not serious | none | ⨁◯◯◯ VERY LOW | Three studies found no difference in Health Related Quality of Life (HRQoL) of ASO patients relative to the general population; two studies found that ASO patients had better HRQoL, while two others found they had lower HRQoL. | | | |

**CI:** Confidence interval; TGA: Transposition of the great Arteries; ASO: Arterial Switch Operation; N/A: Not applicable

**Explanations**

a. There is some evidence of publication bias for smaller studies with lower survival rates but these were unlikely to change the pooled estimates

b. Point estimates vary widely with little or no overlap of the confidence intervals and a substantial heterogeneity (I^2^ 84.31%).

c. The number of participants for whom this outcome is reported is relatively few

d. Some evidence of publication bias yet few studies have actually followed up ASO patients for over 20 years

e. Evidence of publication bias for studies likely to change the overall pooled estimate

f. Few studies reported on this outcome suggestive of selective outcome reporting or publication bias

g. This is an estimate from a single study and we cannot be sure how precise it is

h. The results are quite inconsistent, and differ across the different studies included in the narrative synthesis
