# Supplementary material for: Outcomes after corrective surgery for congenital dextro-transposition of the arteries using the arterial switch technique: a scoping systematic review
Source: Syst Rev. 2020 Oct 7;9:231. doi: 10.1186/s13643-020-01487-3 (PMC7542944; doi:10.1186/s13643-020-01487-3)
Supplement: Supplementary file 5 — Additional file 5. Appendix 5 Sensitivity analysis for the main and secondary outcomes . [file 13643_2020_1487_MOESM5_ESM.docx]

**Appendix 5: Sensitivity analysis for the main and secondary outcomes**

|  | ***Follow up*** | | | | | |
| --- | --- | --- | --- | --- | --- | --- |
| ***Outcome*** | ***Short-term (0-1year)*** | | ***Medium-term (1-20 years)*** | | ***Long-term (>20 years)*** | |
|  | ***% (95% CI)*** | ***Studies (I^2^)*** | ***% (95% CI)*** | ***Studies (I^2^)*** | ***% (95% CI)*** | ***Studies (I^2^)*** |
| ***Survival*** | *92.0 (91.0-92.0)* | *145 (86.9%)* | *89.9 (88.9 -90.8)* | *128 (86.2)* | *87.3^a^ (80.8 - 93.8)* | *4 (87.8)* |
| *Survival in the first era* | *90.0 (89.0 – 91.0)* | *60 (83.8)* | *88.0 (87.0 – 89.0)* | *59 (81.1)* | *89.1^a^ (82.0 – 97.0)* | *3 (N/A)* |
| *Survival in the second era* | *93.0 (92.0 – 94.0)* | *85 (86.4)* | *92.0 (0.91 – 0.93)* | *69 (87.8)* | *81.0 (76.0 – 86.0)* | *1 (N/A)* |
| ***Freedom from reoperation*** | *91.6 (90.0-93.3)* | *38 (90.8)* | *79.0 (76.6-81.5)* | *107 (96.9)* | *77.5 (69.4-85.6)* | *6 (95.5)* |
| *Freedom from reoperation in the first era* | *94.0 (92.0 – 96.0)* | *18 (87.9)* | *81.0 (78.0 – 83.0)* | *60 (96.6)* | *75.0 (63.0 – 86.0)* | *4 (95.5)* |
| *Freedom from reoperation in the second era* | *89.0 (85.0 – 92.0)* | *20 (91.1)* | *77.0 (73.0 – 82.0)* | *47 (97.2)* | *85.0 (83.0 – 87.0)* | *2 (N/A)* |
| ***Aortic insufficiency*** | *9.3 (6.4-12.2) ^a^* | *12 (94. 9)* | *25.0 (21.0 -29.0) ^a^* | *62 (97.5)* | *27.0 (23.0-31.0)* | *2 (N/A)* |
| *Aortic insufficiency in the first era* | *9.0 (1.0-17.0) ^a^* | *4 (95.8)* | *22.0 (17.0 – 28.0) ^a^* | *28 (98.2)* | *41.0 (35.0 – 48.0)* | *1 (N/A)* |
| *Aortic insufficiency in the second era* | *10.0 (4.0 – 15.0) ^a^* | *8 (95.2)* | *27.0 (22.0 – 33.0) ^a^* | *34 (96.3)* | *19.0 (14.0 – 24.0)* | *1 (N/A)* |
| ***Pulmonary stenosis*** | *5.0 (2.0 – 8.0)* | *8 (75.1)* | *14.0 (11.0-16.0) ^a^* | *52 (92.5)* | *82.0 (77.0 – 86.0)* | *1 (N/A)* |
| *Pulmonary stenosis in the first era* | *5.0 (0.0 – 10.0)* | *2 (N/A)* | *13.0 (10.0 – 16.0) ^a^* | *27 (92.8)* | *N/A* | *N/A* |
| *Pulmonary stenosis in the second era* | *5.0 (2.0 – 7.0)* | *6 (85.8)* | *14.0 (11.0 – 18.0) ^a^* | *25 (92.1)* | *82.0 (77.0 – 86.0)* | *1 (N/A)* |
| ***Coronary anomaly*** | *1.0 (0.0 – 3.0)* | *4 (45.8)* | *8.0 (7.0 -10.0)* | *33 (92.4)* | *23.0 (16.0 - 30.0)* | *2 (N/A)* |
| *Coronary anomaly in the first era* | *1.0 (0.0 – 3.0)* | *1 (N/A)* | *4.0 (3.0 – 6.0)* | *15 (89.3)* | *Not assessed ^b^* | *Not assessed ^b^* |
| *Coronary anomaly in the second era* | *2.0 (0.0 – 5.0) ^a^* | *3 (N/A)* | *14.0 (10.0 – 18.0) ^a^* | *19 (93.1)* | *23.0 (16.0 – 30.0)* | *2 (N/A)* |
| ***Neuropsychological outcomes ^c^*** | *N/A* | *N/A* | *N/A* | *N/A* | *N/A* | *N/A* |
| ***Quality of life ^d^*** | *N/A* | *N/A* | *N/A* | *N/A* | *N/A* | *N/A* |

***N/A: Not applicable (in these cases, the outcome was not computed by the statistical software)***

***^a^ Value higher than in main analysis.***

***^b^ Main outcome reported by 1 study hence a sensitivity analysis was not possible.***

***^c^ For Neuropsychological outcomes, the main outcome evaluated as a Z score and pooled directly. A sensitivity analysis was not possible.***

***^d^ For quality of life outcomes, the main outcome was summarized as a narrative synthesis hence a sensitivity analysis was not possible.***
